# Synthesis of Phenyl 2-Acetamidoselenogalactoside Mimetics and Interaction with Amyloid β_1–42_

**DOI:** 10.3390/ph19060836

**Published:** 2026-05-27

**Authors:** João Barros, Nicolas Dreyfus, Gary Sharman, David Evans, Beining Chen, Cleide S. Souza, Gonçalo C. Justino, Maria C. Oliveira, Amélia P. Rauter

**Affiliations:** 1 Centro de Química Estrutural, Institute of Molecular Sciences, Faculdade de Ciências, Universidade de Lisboa, Ed C8, Piso 5, 1749-016 Lisboa, Portugal; jmvbarros@fc.ul.pt; 2 Independent Researcher, Chemin de Joulens, 1, 1110 Morges, Switzerland; ndreyfus@yahoo.com; 3 Independent Researcher, Basingstoke RG25, UK; garysh26@googlemail.com; 4 Independent Researcher, Ascot SL5, UK; dae22@cantab.net; 5 Department of Chemistry, University of Sheffield, Brookhill, Shefield S3 7HF, UK; b.chen@sheffield.ac.uk (B.C.); c.souza@sheffield.ac.uk (C.S.S.); 6 Mass Spectrometry Facility, Centro de Química Estruttural, Instituto Superior Técnico, Av. Rovisco Pais, 1049-001 Lisboa, Portugal; goncalo.justino@tecnico.ulisboa.pt (G.C.J.); conceicao.oliveira@tecnico.ulisboa.pt (M.C.O.)

**Keywords:** phenyl 2-acetamidogalactoside mimetics, selenogalactosides, synthesis, carbohydrate–amyloid β interaction, amyloid disease

## Abstract

**Background/Objectives:** Protein–carbohydrate interactions are implicated in amyloid aggregation pathways associated with Alzheimer’s disease (AD). Designing glycomimetics that modulate amyloid assembly represents a promising strategy. In addition, the interaction of Aβ_1–42_ oligomers (Aβo) with prion protein (PrP^C^) activates Fyn kinase and leads to Tau hyperphosphorylation, another process characterizing AD. Thus, we generated a library of phenyl 2-acetamidoselenogalactoside mimetics to evaluate their interactions with Aβo and disruption of Aβo–PrP^C^ binding, and consequently their potential to inhibit Fyn kinase activation. **Methods:** The synthetic approach comprised azidophenylselenylation, a modified one-pot Staudinger reduction–acylation, a selective α-glycosylation, and deacetylation. Structural diversity was achieved mainly via acylation or ureation. The compounds were screened for binding to Aβo using STD-NMR, ^19^F-NMR, and rapid equilibrium dialysis (RED). ADME properties were assessed through microsomal metabolism and solubility assays, while cytotoxicity was evaluated by MTT assays in human embryonic kidney (HEK) cells. **Results:** Several compounds bound Aβo in STD-NMR experiments, mainly through aromatic and anomeric protons, and phenyl 2-deoxy-2-phenylureido-1-seleno-α-d-galactopyranoside (**34**) showed the most consistent response, with >50% increase in relative binding signal in competition assays, demonstrating also some inhibition of Aβo–PrP^C^ interactions (12%). Selenium at the anomeric position enhanced binding compared to sulphur and oxygen analogs. RED experiments confirmed weak binding interactions, consistent with STD-NMR results. ADME revealed that acetylated compounds undergo microsomal metabolism, whereas deacetylated derivatives displayed high aqueous solubility (>100 μM) and showed no cytotoxicity. **Conclusions:** Phenyl 2-acetamidoselenogalactosides are a novel class of amyloid-binding glycomimetics. Among them, **34** emerges as the most promising compound, combining favorable solubility, metabolic stability, low toxicity, and measurable interference with Aβo and Aβo–PrP^C^ interactions, thus supporting further developments toward therapeutic applications in AD.

## 1. Introduction

Alzheimer’s disease (AD), a multifactorial disorder affecting over 40 million people worldwide, is associated with the formation of Aβ_1–42_ toxic small oligomers, which lead to membrane damage and neuronal death [1]. Although amyloid plaques have long been considered central to AD pathology, current evidence shows that soluble Aβ oligomers (Aβo) are the most neurotoxic species. Their high concentration in AD brains, together with their ability to propagate pathology and inhibit synaptic function, highlights their central role in disease progression. Aβ_1–42_ aggregation follows a nucleation-dependent pathway, progressing from monomers to oligomers, protofibrils and fibrils. However, in addition to Aβ plaques, AD brain contains diffuse deposits of neurofibrillary tangles, composed of hyperphosphorylated Tau. One of the pathways leading to Tau hyperphosphorylation begins with Aβo binding to Prion protein (PrP^C^) at two positively charged *N*-terminal segments, an interaction consistently detected in AD but absent in healthy controls. This binding recruits mGluR5 and activates Fyn kinase, which phosphorylates NMDA receptor subunits and contributes to Tau hyperphosphorylation, dendritic spine loss and synaptic destabilization—mechanisms strongly associated with cognitive decline in AD [2,3].

The search for molecular entities capable of intervening in both processes, either by preventing toxic oligomer formation/Aβo–PrP^C^ interactions or by inducing aggregate disruption, is encouraged for disease prevention or control. It has been reported that structures bearing functional groups which are able to establish π-π interactions and hydrogen bonds are, in most cases, mandatory for Aβ binding [1].

Regarding small carbohydrate entities, it was demonstrated that monosaccharides such as *N*-acetylgalactosamine (GalNAc), *N*-acetylglucosamine, and mannose *O*-glycosylated attached to Ser/Thr side chain of a prion peptide via an α-glycosidic linkage promote the inhibition of amyloidogenesis [4]. However, the same event does not take place with galactose. This suggests that the *N*-acetylamino group in the equatorial position of C2 is important in the interaction between sugar and peptide, leading to the inhibition of amyloid formation. As reported in the literature, the *N*-acetyl sugar moiety linked to Ser135 interacts with the adjacent residue, Arg136, to form two structurally distinguishable conformational populations. Since structural flexibility of Arg136 is important to the nucleation step, a subpopulation that does not favor the ‘amyloid-precursor state’ may retard nucleation [4].

Regarding these insights, GalNAc mimetics represent promising scaffolds for modulating Aβo interactions, particularly when incorporating functional groups capable of enhancing π–π interactions and hydrogen bonding, key contributors to Aβo interactions. In addition, selenogalactosides are molecular entities used in protein interaction studies [5], in which synthetic approaches have been explored [6], and are also found in nature [7]. More recently, new selenosugar conjugates were designed and synthesized, to predict by theoretical calculations their ability to inhibit Aβ oligomerization [8].

In this work, we describe the synthesis of a structurally diverse small library of phenyl 2-acetamidoselenogalactoside mimetics, designed to exploit relevant intermolecular interactions. These compounds were evaluated for their interactions with Aβo and with Aβo–PrP^C^ complexes, aiming to identify glycomimetic structures with potential for modulating toxic amyloid assemblies in AD.

In this context, the present study reports the synthesis of phenyl 2-acetamidoselenogalactoside mimetics and the evaluation of their interactions with Aβ_1–42_ oligomers and the Aβo–PrP^C^ complex. By combining structural modifications at C2 with the anomeric phenylselenyl group, we aimed to explore how amide/urea-type diversity influence amyloid recognition. Our results have identified several mimetics capable of interacting with Aβo, with compound **34** emerging as the most promising hit due to its ADME results and measurable interaction with Aβo and Aβo–PrP^C^ binding, therefore revealing a new class of amyloid-binding compounds with potential to modulate pathogenic Aβ assemblies.

## 2. Results and Discussion

### 2.1. Synthesis of Phenyl 2-Acetamidoselenogalactoside Mimetics

**Scheme 1:**
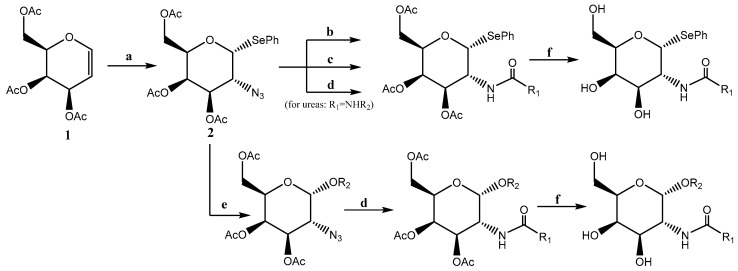
Reagents and conditions: (**a1**) PhSeSePh, NaN_3_, (diacetoxyiodo)benzene, N_2_, r.t., 48 h, 60% yield (after recrystallization) or (**a2**) PhSeSePh, TMSN_3_, (diacetoxyiodo)benzene, N_2_, −30 to −10 °C, 4 h, 92% yield or (**a3**) PhSeSePh, TMSN_3_, (diacetoxyiodo)benzene, N_2_, r.t., 1 h, 81% yield (after recrystallization with propan-2-ol); (**b**) R_1_COCl, Ph_3_P, DCM, r.t., overnight, 62–69% yield; (**c**) (*i*) propane-1,3-dithiol, Et_3_N, DCM, r.t., overnight; (*ii*) (F_3_CCO)_2_O, r.t., 90 min, 90% yield; (**d**) (*i*) Et_3_P (1 M in THF), THF/H_2_O (4:1), r.t., 30 min; (*ii*) R_1_COCl or R_2_NCO, Et_3_N, 4 Å molecular sieves, DCM, r.t., 30–60 min, 40–83% yield (**e**) R_2_OH, I_2_/DDQ, dioxane/toluene (3:1), r.t., overnight (100:9 α:β) 61–92% yield; (**f**) NaOMe (1.2 M in MeOH), MeOH, r.t., 10–30 min, 87–99% yield.

The synthetic strategy employed is summarized in Scheme 1, outlining a unified and modular approach for the preparation of phenyl 2-acetamidoselenogalactoside mimetics. The synthesis started with the anti-Markovnikov azidophenylselenylation (APS) of tri-*O*-acetyl-d-galactal (1,5-anhydro-2-deoxy-d-*ribo*-hex-1-enitol) (**1**) (Scheme 1**a**), involving a C2 azidation and affording phenyl 3,4,6-tri-*O*-acetyl-2-azido-2-deoxy-1-seleno-α-d-galactopyranoside (**2**), the precursor for GalNAc C2-mimetics. The anti-Markovnikov APS of tri-*O*-acetyl-d-galactal was initially performed under heterogeneous NaN_3_-based conditions (Scheme 1(**a1**)) as originally described by Czernecki and co-workers [9], and later under homogeneous TMSN_3_-based conditions following Mironov’s protocol (Scheme 1(**a2**)) [10].

As summarized in Scheme 1**a**, direct comparison of these approaches with the optimized protocol developed in this work (**a3**), which affords the same azidoselenogalactoside intermediate, reveals a marked reduction in reaction time while maintaining comparable isolated yields, establishing **2** as a steadfast and easily accessed precursor for further functionalization.

C2 functionalization was achieved through reduction in the azide followed by in situ acylation or ureation (Scheme 1**b**–**d**). The procedure reported by Maunier and coworkers [11] for anomeric azide acylation, which applied a modified Staudinger reaction, was successfully applied to C2 azide acylation (Scheme 1**b**). In this one-pot process, the azide was reduced with triphenylphosphane and subsequently amidated with the corresponding acyl chloride. Another successful one-pot approach involved reduction in the azide with propane-1,3-dithiol followed by acylation with anhydrides [12,13] (Scheme 1**c**). In addition, we developed the preferred one-pot Staudinger protocol using triethylphosphane (Scheme 1**d**), as the byproduct triethylphosphane oxide could be readily removed by aqueous extraction, simplifying work-up and reducing also the overall reaction time, from overnight to 90 min, while maintaining yield efficiency. This novel one-pot strategy (Scheme 1**d**) provided a more effective and operationally straightforward route to a new series of C2 amide and urea derivatives incorporating aliphatic, aromatic and heteroaromatic substituents (Table 1). These modifications introduced additional hydrogen-bonding capacity and π interactions associated with the additional nitrogen atom, opening perspectives for potentially active analogs.

After purification, deprotection by Zemplén conditions (Scheme 1**f**) afforded the corresponding deacetylated derivatives (Table 1) in high purity, frequently without the need for further purification.

Representative α-*S*-glycosides were prepared, using alternative methods from those described in the literature [14,15], allowing direct comparison of the influence of the anomeric heteroatom (Se, S or O) on amyloid interactions.

**Scheme 2:**
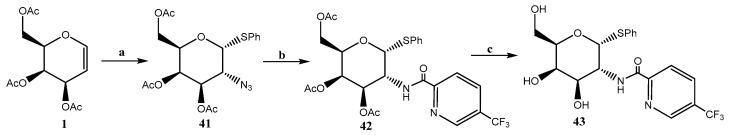
(**a**) PhSSPh, TMSN_3_, (diacetoxyiodo)benzene, N_2_, r.t., 48 h, 9% yield; (**b**) (*i*) Et_3_P (1 M in THF), THF/H_2_O (4:1), r.t., 30 min; (*ii*) 6-(trifluoromethyl)nicotinoyl chloride, Et_3_N, 4Å molecular sieves, DCM, r.t., 30–60 min, 34% yield; (**c**) NaOMe (1.2 M in MeOH), MeOH, r.t., 10–30 min, 97% yield.

The first attempt to synthesize α-thioglycoside mimetics was performed by reacting phenyl 1-selenosugar (**2**) with thiophenol under the same conditions presented at Scheme 1**e**. However, this approach failed to provide the expected product (**41**). Considering the chemical similarity of selenium and sulphur, we investigated the methodology previously applied for phenyl selenogalactosides by reacting d-galactal (**1**) with diphenyldisulfide and TMSN_3_ (Scheme 2). This procedure gave the expected α anomer of phenyl 2-azido thiogalactoside (**41**), albeit in low yield (Scheme 2**a**), indicating a marked influence of the anomeric heteroatom on the efficiency of the azidation step. Amidation and deacetylation were performed as described previously (Scheme 2**b**,**c**), to give compounds **42** and **43**, respectively.

**Scheme 3:**
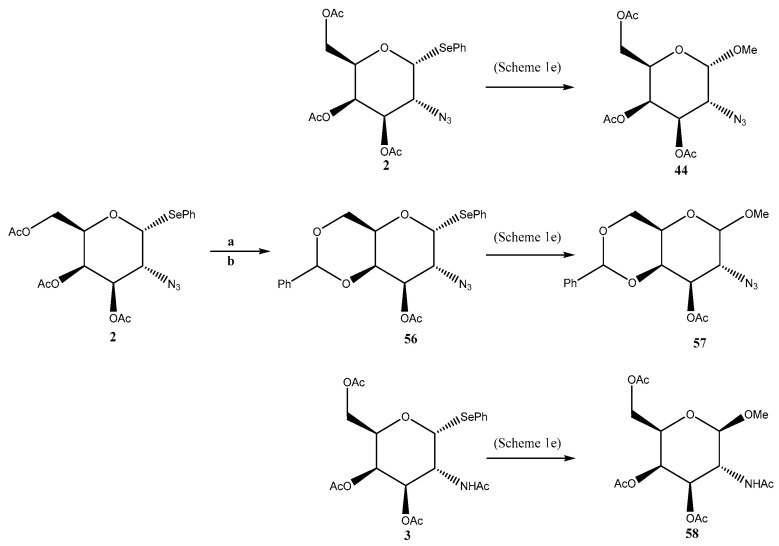
α-O-glycosylation of phenyl selenogalactosides investigation. Reagents and conditions: (**a**) NaOMe (1.2 M in MeOH), MeOH, r.t., 20 min, 92% yield (**55**); (**b**) (*i*) dimethoxymethylbenzene, TsOH·H_2_O, acetonitrile, 80 °C, 25 min; (*ii*) dry DCM, Et_3_N, AcCl, 4 Å molecular sieves, r.t., 20 min, 73% overall yield (two steps); Scheme 1**e** conditions: **44**, 92% yield; **57**, 53% yield (α + β); **58**, 44% yield.

In parallel, α-*O*-glycosylation from selenogalactosides was investigated (Scheme 3) under iodine-mediated conditions (Scheme 1**e**), enabling a direct comparison of anomeric selectivity under identical reaction conditions. When applied to **2**, the reaction afforded predominantly the α-anomer (**44**, Scheme 3), in agreement with the reported iodine-mediated glycosylation methodology [16]. In order to investigate the influence of donor structure on the stereochemical outcome, the same glycosylation conditions were applied to structurally related starting materials differing in protecting-group patterns (**56**, Scheme 3) and substitution at C2 (**3**, Scheme 3).

^1^H NMR analysis revealed different α/β ratios outcomes for each donor **2**, 100/8; **56**, 50/100; **3**, β only), highlighting clear differences in anomeric selectivity, despite unchanged reaction conditions. The thermodynamically most stable α-anomer was formed as the major product (**44**) due to the anomeric effect and solvent influence [17], while the neighboring effect induced the formation of the β-anomer, as the only compound (**58**) [18]. Interestingly, protection of the positions 4 and 6 with benzylidene caused a loss of stereoselectivity, with the presence of an α:β anomeric ratio = 1:2 (**57**). Taken together, the data presented demonstrate that anomeric selectivity in this α-*O*-glycosylation protocol is governed not only by the reaction conditions but also by intrinsic donor structural effects, including protecting-group pattern and neighboring-group participation. This tunability is particularly relevant for controlling the stereochemical reaction outcome and, consequently, the functional groups in the resulting glycomimetics.

α-*O*-galactosides were accessed from **2** via iodine-mediated glycosylation (Scheme 1**e**), affording the desired products in good yields. Subsequently, the same C2 functionalization and deprotection sequence was applied.

Phenyl α-*O*-glycosides (**51**, **52**) differ from the corresponding thiogalactosides (**42**, **43**) and selenoglycosides (**19**, **20**) analogs only in the exo-anomeric atom.

This unified synthetic approach enabled the preparation of a structurally diverse library of phenyl 2-acetamidoselenogalactoside mimetics (Table 1 and Table 2), suitable for systematic evaluation of structure–activity relationships toward amyloid species.

### 2.2. Molecular Interactions with Amyloid Species

#### 2.2.1. Affinity Selection Mass Spectrometry (ASMS) Experiments

ASMS assays are simple binding experiments in which a mixture of small molecules is incubated with a target protein, allowing potential interactions to occur. The resulting mixture is then analyzed by LC–MS to identify bound ligands. As a positive control, carbonic anhydrase was incubated with ethoxzolamide, a well-known inhibitor of this enzyme [19]. LC–MS analysis of the filtrates revealed a 35% relative response for ethoxzolamide (values > 5% are considered indicative of binding).

**Figure 1:**
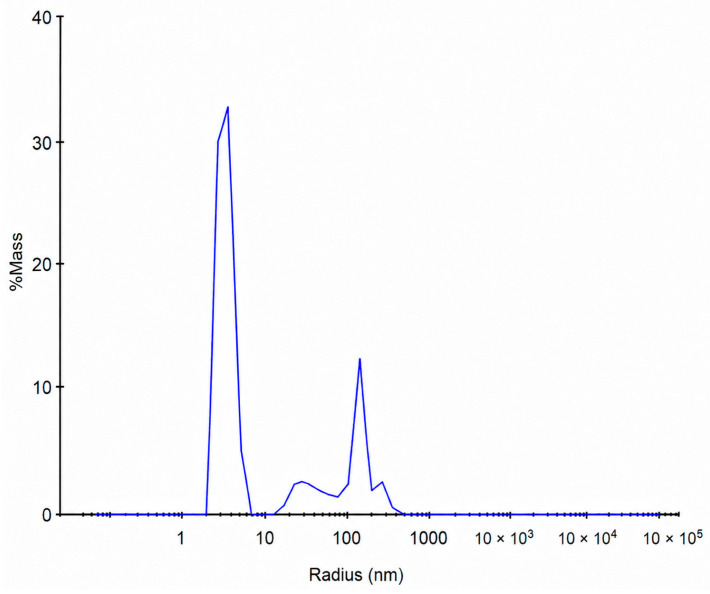
DLS confirmation of the radii of freshly prepared Aβo used in molecular interaction screening.

This methodology was next applied to freshly prepared Aβ_1–42_ oligomers (Aβo), whose size distribution was confirmed by dynamic light scattering (DLS). The majority of aggregates exhibited radii between 1 and 10 nm (Figure 1).

However, when the control experiment was performed with bexarotene, a reported Aβo ligand [20], the compound failed to cross the centrifugal filter, even in the absence of protein. Application of this protocol to our compounds resulted in non-reproducible data. Possible explanations include interactions that are too weak to be detected under these conditions, or compound-dependent retention during filtration. Given these limitations, we turned to alternative methodologies, in particular STD-NMR.

#### 2.2.2. STD-NMR and ^19^F-NMR Experiments

**Figure 2:**
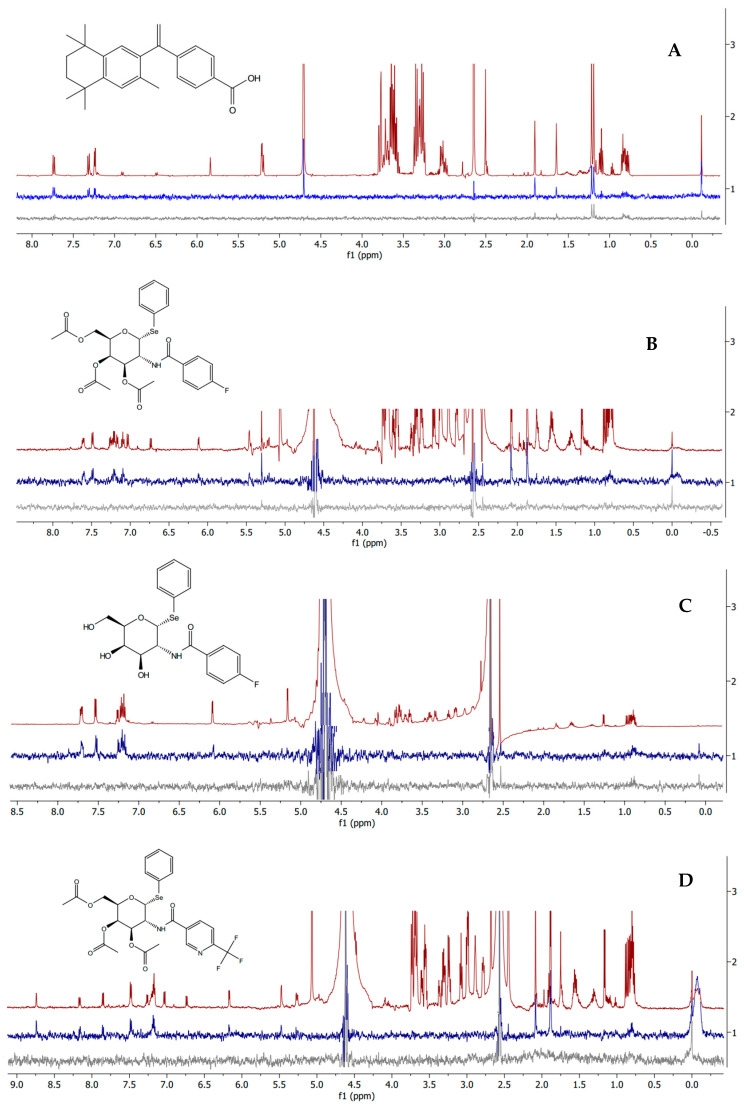

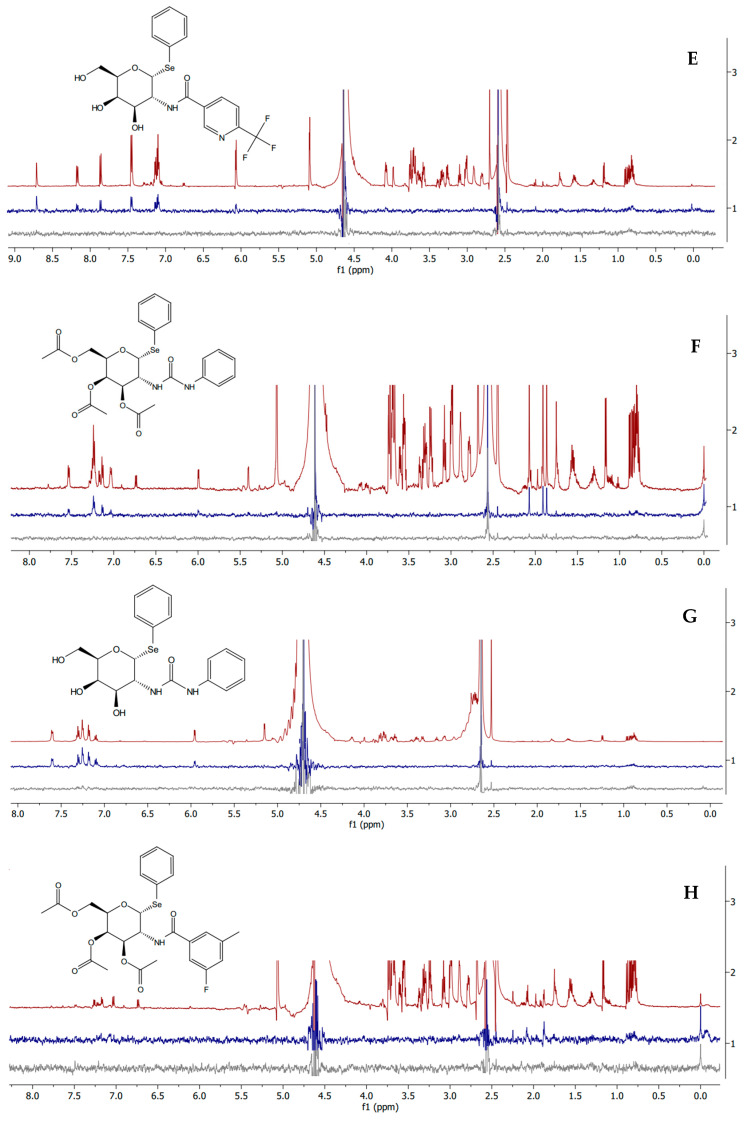

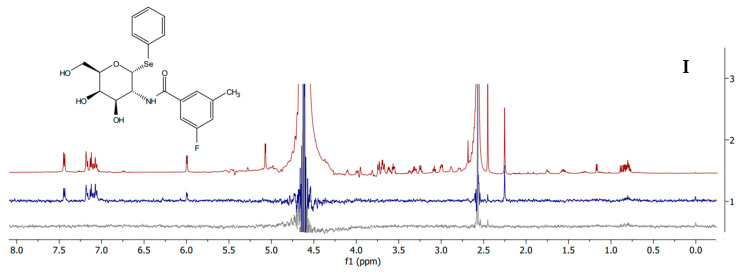
STD-NMR experiments to evaluate the binding affinity of selenogalactosides against Aβo. Reference (REF, red) and difference (DIFF, blue) spectra were recorded in DMSO with freshly prepared Aβo (2 μM) in a deuterated phosphate buffer (pH 7.4) and neurobasal medium. Final compound concentration: 200 μM (1:100 molar ratio). Control (CTRL, gray spectrum) experiments were performed without Aβo. (**A**) Bexarotene, (**B**) 11; (**C**) 12; (**D**) 19; (**E**) 20; (**F**) 33; (**G**) 34; (**H**) 15; (**I**) 16.

Control STD-NMR experiments were first optimized using bovine serum albumin (BSA) with ibuprofen as a positive and glucose as a negative control [21]. As a positive control test against Aβo, bexarotene was employed, consistent with its reported high-affinity binding to Aβo and its ability to block primary and secondary nucleation in amyloid aggregation [22], thereby validating the methodology (Figure 2A). In STD-NMR experiments with our compounds, both acetylated and deacetylated forms showed binding to Aβo, primarily through aromatic and anomeric protons (**11**, **12**, **19**, **20**, **33**, **34**, **16**). No significant interaction was observed for **15**, except for signals from acetyl protons, as observed for most acetylated derivatives (Figure 2).

**Figure 3:**
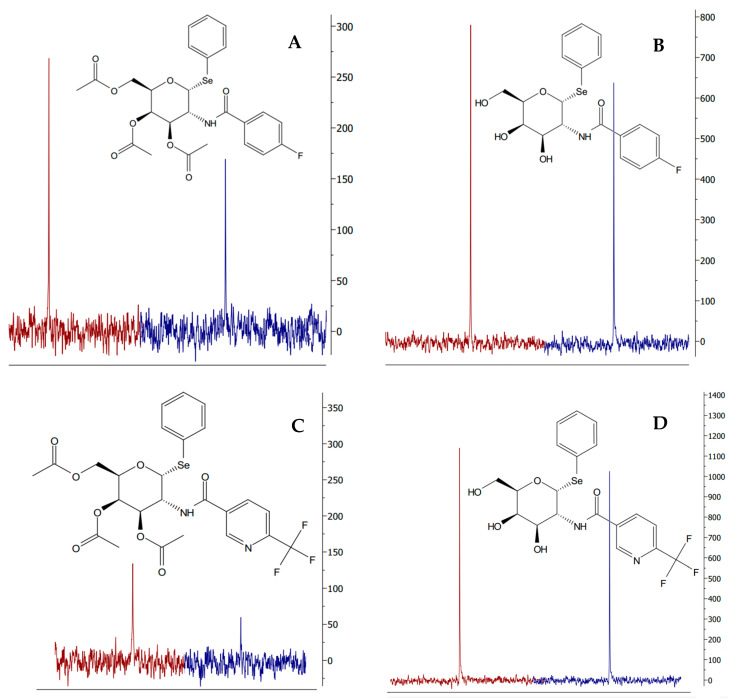
^19^F-NMR CPMG experiments confirming STD-NMR results for phenylselenogalactosides against Aβo. For reference, without Aβo (red) and with Aβo (blue), in the same conditions as previous STD-NMR experiments. (**A**) 11; (**B**) 12; (**C**) 19; (**D**) 20.

To further confirm these interactions, ^19^F-NMR Carr-Purcell-Meiboom-Gill (CPMG) experiments were performed under the same conditions. Compounds were considered positive binders when the decrease in signal intensity in the presence of Aβo was, at least, twice the noise level. These experiments confirmed the STD-NMR results for fluorinated derivatives (Figure 3).

In parallel, metabolic and ADME solubility experiments were performed (Section 2.3). Based on these results, STD-NMR screening was restricted to deacetylated phenylselenogalactoside C2 derivatives, as summarized in Table 3. Binding was assessed qualitatively based on STD-NMR difference spectra (e.g., Figure 2). Compounds were classified as positive (+) when STD signals were observed above the noise level and matched ligand resonances in the reference spectrum. Compounds showing no detectable STD signals under identical conditions were classified as non-binders (−). While this classification provides a qualitative assessment of interaction, relative STD intensities were used to compare interaction levels among positive compounds.

**Figure 4:**
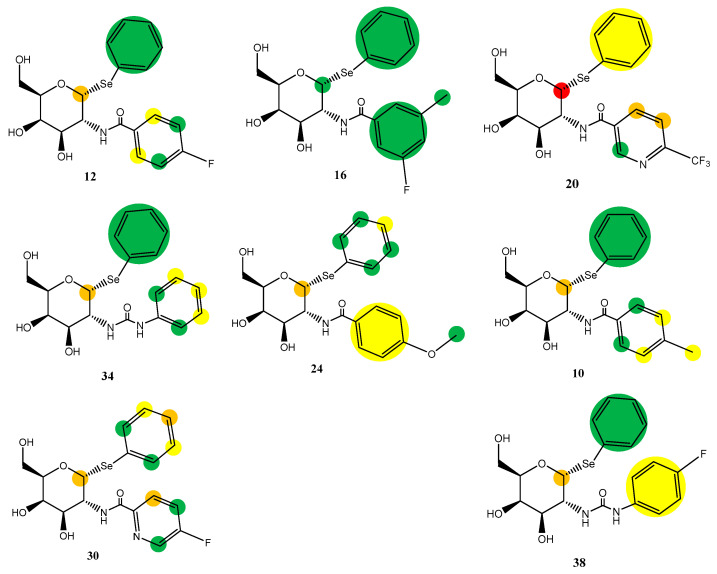
Compounds showing ≥5% binding to Aβo in STD-NMR assays (proton mapping). Relative percentages of spin saturation: green (100–75), yellow (74–55), orange (54–25), red (24–1).

Proton mapping was performed for positive hits by comparing the reference peak area (P_R_) with the difference peak area (P_D_), from STD-NMR results, using the equation (P_D_/P_R_) × 100. Based on these results, we are able to compare the relative binding strength among the different positive compounds. Considering the interaction levels observed for bexarotene (5–15%), a threshold of ≥5% was established as significant. Based on this condition, eight compounds were identified as preferable positive binders (Figure 4).

Although these interactions fall within the weak-binding regime detected by STD-NMR, they are consistent with transient and multivalent contacts expected for amyloid oligomer recognition, as described for dynamic amyloid assemblies [23,24].

**Figure 5:**
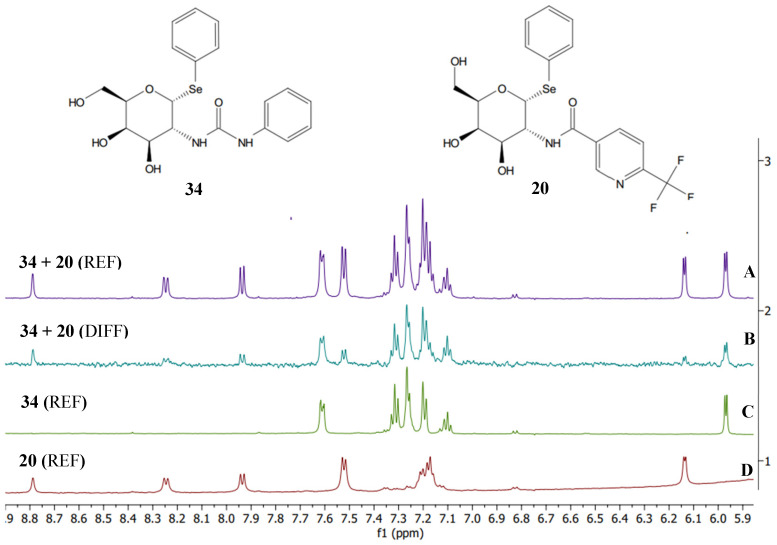
STD-NMR competition assay example, between compound **34** and **20** towards Aβo. (**A**) Mixture reference (REF) between compound **34** and **20**, both at 200 μM; (**B**) mixture difference (DIFF) between compound **34** and **20**, both at 200 μM; (**C**) compound **34** (REF) at 200 μM; (**D**) compound **20** at 200 μM. For reference (REF) and difference (DIFF) spectra, compounds were added in DMSO to a 2 μM solution of freshly prepared Aβo in a mixture of deuterated phosphate buffer (pH 7.4) and neurobasal medium. Control REF experiments were carried out in the same conditions, with 4% DMSO, but in the absence of Aβo.

Based on these results, STD-NMR competition experiments were undertaken between these binders against Aβo (Figure 5). Binding variation in each compound (B_comp_) in the competition experiment was calculated considering the initial binding percentage (B_i_) as [(B_comp_ − B_i_)/B_i_] × 100, with ±50% considered significant. Only non-overlapping proton signals between paired compounds were analyzed.

The most prominent hit was **34**, which showed >50% increase in binding percentage in all competition assays. In most cases, competitor binding decreased, except for **10** and **24**, which also increased, though less markedly. These results may suggest possible cooperative or non-exclusive binding modes, potentially involving protein conformational changes that expose additional binding sites.

In contrast, **20** showed the largest reduction in binding percentage, possibly related to its higher electronegative atom density at the C2 moiety, when compared to **34**, indicating that either a lack or an excess of electronegative substituents may disrupt optimal interactions.

**Figure 6:**
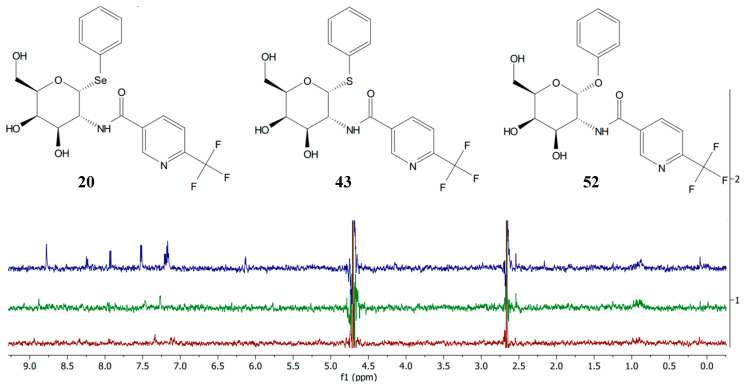
STD-NMR experiments to compare the binding affinity of *Se*-, *S*- and *O*-galactosides against Aβo. Difference spectra: **20** (blue), **43** (green), **52** (red).

Finally, to investigate the relevance of the selenium atom at the anomeric position, STD-NMR assays were performed with *Se*-, *S*-, and *O*-glycosides (**20**, **43**, and **52**, respectively). The results confirmed that selenium at the anomeric center enhances the binding affinity for Aβo (Figure 6).

#### 2.2.3. Rapid Equilibrium Dialysis (RED)

Rapid equilibrium dialysis (RED) is a solution-based method used to evaluate the binding strength of small molecules to proteins [25]. The system consists of a two-chamber vial separated by a semipermeable membrane that permits only small molecules to diffuse between compartments. Because Aβo are large aggregates (≈50 kDa, 5–10 nm), they cannot cross the membrane. In practice, one chamber contains the protein with test compounds, while the other contains only buffer. After shaking for 4 h, the buffer chamber is analyzed by LC–MS for the presence of small molecules, and results are compared to a control vial without protein. Typically, only strong binders (Kd < 10 nM) are detected with this method.

Since STD-NMR primarily detects weak binders, we hypothesized that compounds **43** and **52** might represent strong binders undetected by STD-NMR. To investigate this, RED experiments were performed with these compounds and with **20**, for comparison. Each assay was performed in triplicate, and the results are presented in Table 4.

The results were consistent with the previous STD-NMR data (Figure 6), showing that none of the compounds behave as strong binders. Among them, the selenium derivative **20** exhibited comparatively higher affinity, although the large uncertainties associated with these values avert firm conclusions. These findings suggest that the interactions of these compounds with Aβo occur in a weak-binding regime and cannot be reliably quantified under these RED conditions, thereby confirming STD-NMR as the most suitable tool for detecting such low-affinity interactions. The consistently enhanced interaction profile observed for the selenium-containing derivatives suggests that this behavior may be related to the intrinsic properties of selenium. Compared to sulphur and oxygen, selenium is more polarizable, which can enhance weak intermolecular interactions and favor transient contacts with the dynamic surface of Aβo. Moreover, the longer C–Se bond and the softer electronic character at the anomeric position may subtly influence the conformation of the sugar scaffold, potentially facilitating more effective multivalent interactions with amyloid assemblies.

A more detailed analysis of the structure–activity relationship (SAR) within this compound series reveals that, besides selenium atom at the anomeric position, the nature of the substituent at the C2 position is essential for interaction with Aβo. Simple aliphatic amido groups such as acetamido or trifluoroacetamido (**4** and **6**) do not promote detectable interaction, indicating that a certain aromatic structure is required. However, the presence of the aromatic moiety alone is not sufficient to guarantee binding. While several aryl-substituted derivatives (e.g., **10**, **12**, **16** and **18**) display positive STD-NMR responses, others bearing the unsubstituted aromatic group (**8**), as well as naphthyl (**22**), do not, suggesting that both steric and electronic factors contribute to modulating the interaction ability.

Heteroaromatic substituents are also tolerated, although their behavior is variable, with some derivatives exhibiting interaction (e.g., **20** and **36**), while others do not (e.g., **28** and **32**), indicating that subtle differences in electronic distribution and geometry influence the binding process.

In addition, the presence of the amido or ureido group also plays an important role. Both functionalities are compatible with binding. However, ureido-containing derivatives (e.g., **34**, **36** and **38**) tend to display consistent STD-NMR responses, which may be related to their increased hydrogen-bonding capacity and conformational restriction compared to simple amides. This effect is further highlighted when comparing compound **34** with compound **8**, which bears an aromatic substituent directly attached to the sugar but does not exhibit detectable interaction, indicating that ureido group presence plays an important role in effective recognition.

Finally, deacetylation leads to a change in the interaction pattern rather than a loss of binding. While acetylated derivatives predominantly show STD effects associated with acetyl methyl groups, deacetylated analogs exhibit the pronounced interactions involving aromatic and anomeric protons. This shift likely reflects differences in the regions of the molecule engaged in the interaction, rather than a significant change in overall binding affinity.

### 2.3. Metabolic Experiments

#### 2.3.1. Microsomal Metabolism and ADME Solubility

Microsomal metabolism experiments demonstrated that all acetylated compounds were metabolized by liver microsomes from different species, whereas deacetylated compounds were not (Table 5). These results indicate that microsomes enzymes efficiently promote deacetylation of the tested compounds but do not promote further metabolic degradation. Analysis of ADME solubility revealed that deacetylated derivatives displayed markedly higher solubility in aqueous buffer (pH 7.4), with concentrations exceeding 100 μM. This enhanced solubility can be attributed to the presence of hydroxyl groups at C3, C4, and C6, which increase molecular polarity and may favor bioavailability.

To complement the experimental ADME evaluation, physicochemical descriptors were calculated for representative acetylated and deacetylated derivatives using SwissADME (Table 6) [26]. Analyzing these results, a clear distinction between acetylated and deacetylated compounds is observed, being consistent with the SAR analysis described above. Acetylated compounds (**19**, **11**, **15** and **33**) display higher molecular weights and increased lipophilicity (logP ~1.1–1.9), consistent with their reduced aqueous solubility and extensive microsomal metabolism. In contrast, deacetylated analogs (**20**, **12**, **16** and **34**) exhibit lower molecular weights, reduced logP values (~0.0–0.8), and increased polarity, in agreement with their improved water solubility (>100 μM). Deacetylated derivatives show lower TPSA values than acetylated compounds. This behavior reflects that solubility is more strongly governed by hydrogen-bonding ability and overall polarity balance than by TPSA alone. Notably, all deacetylated compounds comply with Lipinski’s rule of five, whereas acetylated derivatives generally present one violation, mainly due to their higher molecular weight. Within this series, compound **34** stands out by combining an optimal physicochemical balance with high aqueous solubility and a favorable interaction profile, supporting its identification as a promising lead for further optimization.

#### 2.3.2. Toxicity Experiments and Aβo–PrP^C^ Interaction Disruption

**Figure 7:**
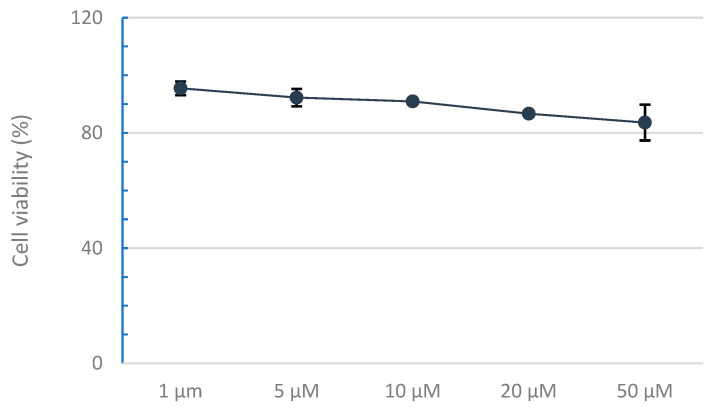
MTT assay results for **34** at different concentrations.

To assess cytotoxicity, MTT assays were performed using human embryonic kidney (HEK) cells. Most compounds showed no significant toxicity. Only **16** displayed reduced viability (57% ± 7%) at concentrations ≥ 50 μM. For **34**, cell viability ranged from 84% ± 6% at 50 μM to 95% ± 2% at 1 μM (Figure 7).

Next, we examined whether **34** could interfere with Aβo–PrP^C^ interactions. Using HEK cells, where PrP^C^ is endogenously expressed, **34** promoted a 12% inhibition of this interaction. Although modest, this result supports the potential of **34** as a lead compound for the development of novel strategies targeting Alzheimer’s disease. These findings highlight **34** as a particularly promising scaffold, combining metabolic stability, high solubility, low cytotoxicity, and measurable inhibition of Aβo–PrP^C^ binding.

## 3. Materials and Methods

### 3.1. Chemistry

HPLC-grade solvents and reagents were obtained from commercial suppliers and used without further purification. Tri-*O*-acetyl-d-galactal (**1**) was purchased from Sigma Aldrich. Flash column chromatography (CC) was performed using silica gel 60 Å (0.060–0.200 mm, Merck, Darmstadt, Germany), either manually or on a CombiFlash^®^ Rf200 (Teledyne Isco, Lincoln, NE, USA). Reactions were monitored by analytical thin layer chromatography (TLC), using silica gel 60 F-254 plates (Merck) with visualization under ultraviolet (UV) light (λ = 254 nm), and by spraying with a 10% H_2_SO_4_ solution in EtOH, followed by heating at 120 °C, or by LC–MS experiments, performed on a column XBridge C18 (3.5 μm, 2.1 × 50 mm at 1.2 mL/min and 50 °C; Waters Corporation, Milford, MA, USA); 10 mM ammonium bicarbonate (pH 9)/ACN, gradient 10 > 95% ACN in 1.5 min + 0.5 min hold. Preparative HPLC was performed in a Gilson apparatus using either Phenomenex gemini NX (Phenomenex, Torrance, CA, USA), C18, 5 μm 30 × 100 mm or Phenomenex gemini NX, C18, 10 μm 50 × 150 mm columns. NMR spectra for compound characterization were recorded on a Bruker AV III HD Nanobay spectrometer (Bruker Biospin, Rheinstetten, Germany) operating at 400.13 MHz and equipped with a room temperature 5 mm BBO Smartprobe with Z-gradients capable of ^19^F observation. Chemical shifts are expressed in δ (ppm) and the proton coupling constants *J* in hertz (Hz). NMR data were assigned using standard COSY, HMQC, and HMBC experiments. For the characterization of anomeric rings—phenyl-α-1-selenogalactosides, phenyl-α-1-thiogalactosides and phenyl-α-galactosides—protons and carbons are assigned as H″, C″; in other aromatic rings, protons and carbons are assigned as H′, C′. Melting points were measured using an SMP3 melting point apparatus (S Stuart Scientific, Bibby Scientific Ltd., Stone, Staffordshire, UK; room temperature < m.p. < 360 °C). Optical rotations were measured using an Anton Paar MCP 500 polarimeter (Anton Paar GmbH, Graz, Austria). High-resolution mass spectra (HRMS) were obtained on a QqTOF Impact II^TM^ mass spectrometer (Bruker Daltonics, Bremen, Germany), operating in the ESI positive mode. Samples were analyzed by Flow Injection Analysis (FIA) using an isocratic gradient 50 A:50 B of 0.1% formic acid in methanol (A) and ammonium acetate (2.5 mM) in water (B), at a flow rate of 10 µLmin^−1^ over 5 min. Internal calibration was achieved with an ammonium formate solution introduced to the ion source via a 20 µL loop using a six-port valve, at the ending of each analysis. The full scan mass spectra were acquired over a mass range of 100–900 *m*/*z* at a spectra rate of 1 Hz. Data was processed using Data Analysis 5.1 software.

#### 3.1.1. General Procedures **a3**, **b**, **c**, **d**, **e**, and **f**

**a3**: Compound **1** and Ph_2_Se_2_ (1 eq.) were dissolved in dry DCM (0.2 mmol/mL). Then, PhI(OAc)_2_ (1 eq.) and TMSN_3_ (2 eq.) were added. The mixture was stirred at room temperature, under a nitrogen atmosphere and monitored by LC–MS. After 1 h, when the starting material disappeared, the mixture was evaporated, and the crude submitted to column chromatography: hexane to ethyl acetate (0% to 25% (3:1)). The recovered material was recrystallized from isopropanol (propan-2-ol).

**b**: 2-azido-2-deoxyglycoside was dissolved in DCM (0.1 mmol/mL) and R_1_COCl (4 eq.) was added. Under a nitrogen environment, at room temperature and stirring, a solution of Ph_3_P (3 eq.) in DCM (0.33 mmol/mL) was added dropwise. The reaction was stirred at room temperature overnight. After completion, the reaction mixture was extracted with DCM and washed with water (4 × 10 mL) followed with saturated NaHCO_3_ (4 × 10 mL). The organic layer was combined and dried with MgSO_4_, filtered and evaporated under vacuum. The residue was purified by preparative HPLC, unless otherwise stated, and dried under vacuum.

**c**: 2-azido-2-deoxygycoside was dissolved in DCM (0.11 mmol/mL). Triethylamine (4 eq.) and 1,3-propanodithiol (4 eq.) were added. The reaction was stirred at room temperature overnight, in nitrogen atmosphere. After all the starting material disappeared, anhydride (4 eq.) was added. The reaction was stirred at room temperature for 1.5–4 h. The reaction mixture was washed with saturated NaHCO_3_ (3 × 10 mL) and water (3 × 10 mL). The organic layer was combined and dried with MgSO_4_, filtered and evaporated under vacuum. The residue was purified by preparative HPLC, unless otherwise stated, and dried under vacuum.

**d**: 2-azido-2-deoxygycoside was dissolved in THF/H_2_O (1 g to 28 mL/7 mL). Et_3_P (1.3 eq., 1M in THF) was added, and the reaction mixture was stirred, at room temperature, for 30 min, until all starting material disappeared. The reaction mixture was evaporated, and the crude was dissolved in dry DCM (0.0425 mmol/mL) and activated 4 Å molecular sieves were added. While gently stirring, triethylamine (3 eq.) was added. After, the anhydride or acyl chloride or isocyanate (3 eq.) was added dropwise, and the reaction mixture was stirred for 30–60 min. All reactions were followed by LCMS. Reaction mixture was washed with water and saturated NaHCO_3_ and extracted with DCM (3 × 15 mL). The organic layers were combined, dried with MgSO_4_, filtered and concentrated under vacuum. The residue was purified by preparative HPLC, unless otherwise stated, and dried under vacuum.

**e**: Compound **2** was dissolved in dioxane/toluene (3:1, 0.22 mmol/mL) and methanol (4 eq.) was added. After addition of I_2_ (1.5 eq.) and DDQ (1.5 eq.) the reaction was stirred, at room temperature, and followed by LCMS. After 4 h, when the starting material disappeared, the mixture was quenched with a solution of sodium thiosulfate, extracted with DCM, and washed with brine (3 × 10 mL). The organic layer was combined and dried with MgSO_4_, filtered and evaporated under vacuum. The residue was purified by preparative HPLC, unless otherwise stated, and dried under vacuum.

**f**: Acetylated compound was dissolved in CH_3_OH (e.g., 0.200 mg in 3.00 mL). NaOCH_3_ solution (1.2 M in methanol; 0.1 mL NaOCH_3_ solution to 100 mg of acetylated compound) was added. The reaction mixture was stirred until all starting material was consumed (10–30 min). Upon completion, the mixture was neutralized with Amberlite IR 120 (H-form), filtered, and evaporated. In most cases, no further purification was required.

#### 3.1.2. Compound Characterization

Phenyl 3,4,6-tri-*O*-acetyl-2-azido-2-deoxy-1-seleno-α-d-galactopyranoside (**2**)

**Procedure a3**: Yield: 81%; LC–MS (high-pH method): Rt = 1.26 min; *m*/*z* = 489.000 [M+OH]^−^; white solid; m.p. = 104.0–105.0 °C; **^1^H NMR** (400 MHz, CDCl_3_) δ 7.61 (d, 2H, H-2″, H-6″), 7.35–7.23 (m, 3H, H-3″, H-4″, H-5″), 6.01 (d, *J*_1,2_ = 5.4 Hz, 1H, H-1), 5.48 (d, *J*_4,3_ = 2.3 Hz, 1H, H-4), 5.12 (dd, *J*_3,2_ = 10.9, *J*_3,4_ = 3.2 Hz, 1H, H-3), 4.67 (brt, *J*_5,6a_ = *J*_5,6b_ = 6.5 Hz, 1H, H-5), 4.27 (dd, *J*_2,3_ = 10.8, *J*_2,1_ = 5.4 Hz, 1H, H-2), 4.09, 4.06 (part AX of ABX system, *J*_AB_ = 11.4, *J*_AX_ = 5.9 Hz, 1H, H-6a); 4.04, 4.01 (part BX of ABX system, *J*_BA_ = 11.4, *J*_BX_ = 7.1 Hz, 1H, H-6b), 2.16 (s, 3H, CH_3_), 2.07 (s, 3H, CH_3_), 1.98 (s, 3H, CH_3_). **^13^C NMR** (101 MHz, CDCl_3_) δ 170.3, 169.9, 169.6 (3 × CO), 134.7 (C-2″, C-6″), 129.2 (C-3″, C-5″), 128.2 (C-4″), 127.5 (C-1″), 84.1 (C-1), 71.2 (C-3), 68.9 (C-5), 67.1 (C-4), 61.5 (C-6), 58.7 (C-2), 20.6 (3 × CH_3_). HRMS (ESI) *m*/*z*: calcd for C_18_H_25_N_3_O_7_Se [M+NH_4_]^+^ 489.0884; found 489.0920. Data are in full agreement with those previously reported [5].

Phenyl 2-acetamido-3,4,6-tri-*O*-acetyl-2-deoxy-1-seleno-α-d-galactopyranoside (**3**)

**Procedure b**: Starting material: **2**. The reaction was followed by TLC (Rf = 0.41, EtOAc/Hex 1:1). Purification was performed by silica gel column chromatography (1:2 to 2:1 EtOAc/Hex). Yield: 62%; LC–MS (high-pH method): Rt = 1.00 min; *m*/*z* = 489.000 [M+H]^+^; yellow syrup. **Procedure d**: Starting material: **2**. Yield: 66%; yellow syrup; ${\left[\alpha \right]}_{D}^{20}$ = +136.1 (*c* 2.38 CH_2_Cl_2_); **^1^H NMR** (400 MHz, CDCl_3_) δ 7.64–7.48 (m, 2H, H-2″, H-6″), 7.35–7.19 (m, 3H, H-3″, H-4″, H-5″), 6.85 (d, *J*_*NH*,2_ = 7.8 Hz, 1H, NH), 6.22 (d, *J*_1,2_ = 5.1 Hz, 1H, H-1), 5.51 (dd, *J*_4,3_ = 3.2 Hz, *J*_4,5_ = 1.3 Hz, 1H, H-4), 5.19 (dd, *J*_3,2_ = 11.8 Hz, *J*_3,4_ = 3.2 Hz, 1H, H-3), 4.79–4.71 (m, 2H, H-2, H-5), 4.13, 4.11 (part AX of ABX system, *J*_AB_ = 11.3, *J*_AX_ = 6.1 Hz, 1H, H-6a); 4.04, 4.01 (part BX of ABX system, *J*_BA_ = 11.3, *J*_BX_ = 6.9 Hz, 1H, H-6b), 2.14 (s, 3H, CH_3_), 2.02 (s, 3H, CH_3_), 2.00 (s, 3H, CH_3_), 1.87 (s, 3H, CH_3_). ^**13**^**C NMR** (101 MHz, CDCl_3_) δ 169.2, 169.0, 168.6, 168.5 (4 × CO), 132.6 (C-2″, C-6″), 127.5 (C-3″, C-5″), 126.3 (C-4″), 126.0 (C-1″), 85.4 (C-1), 67.6 (C-5), 67.5 (C-3), 65.2 (C-4), 59.9 (C-6), 47.2 (C-2), 21.3, 19.0, 18.8, 18.7 (4 × CH_3_). HRMS (ESI) *m*/*z*: calcd for C_20_H_25_NO_8_Se [M+H]^+^ 488.0819; found 488.0840.

Phenyl 2-acetamido-2-deoxy-1-seleno-α-d-galactopyranoside (**4**)

**Procedure f**: Starting material: **3**. Yield: 98%; yellow syrup; ${\left[\alpha \right]}_{D}^{20}$ = +263.8 (*c* 0.80 MeOH); **^1^H NMR** (400 MHz, MeOD) δ 7.59–7.53 (m, 2H, H-2″, H-6″), 7.23 (m, 3H, H-3″, H-4″, H-5″), 6.06 (d, *J*_1,2_ = 5.0 Hz, 1H, H-1), 4.34 (dd, *J*_2,3_ = 11.2, *J*_2,1_ = 5.1 Hz, 1H, H-2), 4.20 (brt, *J*_5,6a_ = *J*_5,6b_ = 6.1 Hz, 1H, H-5), 3.94 (d, *J*_4,3_ = 2.4 Hz, 1H, H-4), 3.75–3.61 (m, 3H, H-3, H-6a, H-6b), 1.95 (s, 3H, CH_3_). ^**13**^**C NMR** (101 MHz, MeOD) δ 174.0 (CO), 135.7 (C-2″, C-6″), 130.7 (C-1″), 130.1 (C-3″, C-5″), 128.6 (C-4″), 89.4 (C-1), 75.2 (C-5), 70.3 (C-3), 69.9 (C-4), 62.3 (C-6), 53.0 (C-2), 22.6 (CH_3_). HRMS (ESI) *m*/*z*: calcd for C_14_H_19_NO_5_Se [M+H]^+^ 362.0502; found 362.0520.

Phenyl 3,4,6-tri-*O*-acetyl-2-deoxy-2-trifluoroacetamido-1-seleno-α-d-galactopyranoside (**5**)

**Procedure c**: Starting material: **2**. The reaction was followed by TLC: step one: Rf = 0.2 (EtOAc/Hex 1:2); step two: Rf = 0.46 (EtOAc/Hex 1:2). Purification was performed by silica gel column chromatography (1:5 to 1:4 EtOAc/ Hex). Yield: 90%; transparent syrup. **Procedure d**: Starting material: **2**. Yield: 83%; LC–MS (high-pH method): Rt = 1.21 min; *m*/*z* = 559.000 [M+NH_4_]^+^; transparent syrup; ${\left[\alpha \right]}_{D}^{20}$ = +188.4 (*c* 1.72 CH_2_Cl_2_); **^1^H NMR** (400 MHz, CDCl_3_) δ 7.65–7.53 (m, 2H, H-2″, H-6″), 7.47 (d, *J*_*NH*,2_ = 8.4 Hz, 1H, NH), 7.37–7.25 (m, 3H, H-3″, H-4″, H-5″), 6.06 (d, *J*_1,2_ = 5.0 Hz, 1H, H-1), 5.56 (dd, *J*_4,3_ = 3.3 Hz, *J*_4,5_ = 1.3 Hz, 1H, H-4), 5.23 (dd, *J*_3,2_ = 11.7 Hz, *J*_3,4_ = 3.3 Hz, 1H, H-3), 4.90–4.69 (m, 2H, H-2, H-5), 4.17, 4.14 (part AX of ABX system, J_AB_ = 11.3, J_AX_ = 6.3 Hz, 1H, H-6a); 4.11, 4.08 (part BX of ABX system, J_BA_ = 11.3, J_BX_ = 6.9 Hz, 1H, H-6b), 2.17 (s, 3H, CH_3_), 2.02 (s, 3H, CH_3_), 1.88 (s, 3H, CH_3_). **^13^C NMR** (101 MHz, CDCl_3_) δ 171.3, 170.3, 170.2 (3 × CO), 157.5 (q, *J* = 37.9 Hz, CO), 134.7 (C-2″, C-6″), 129.5 (C-3″, C-5″), 128.6 (C-4″), 127.3 (C-1″), 115.6 (q, *J* = 287.8 Hz, CF_3_), 86.9 (C-1), 69.8 (C-5), 69.2 (C-3), 66.8 (C-4), 61.4 (C-6), 49.6 (C-2), 20.6, 20.4, 20.4 (3 × CH_3_). HRMS (ESI) *m*/*z*: calcd for C_20_H_26_F_3_NO_8_Se [M+NH_4_]^+^ 559.0802; found 559.0847.

Phenyl 2-deoxy-2-trifluoroacetamido-1-seleno-α-d-galactopyranoside (**6**)

**Procedure f**: Starting material: **5**. Yield: 95%; LC–MS (high-pH method): Rt = 0.82 min; *m*/*z* = 433.000 [M+NH_4_]^+^; yellow syrup; ${\left[\alpha \right]}_{D}^{20}$ = +278.6° (*c* 0.63 MeOH); ^**1**^**H NMR** (400 MHz, MeOD) δ 7.63–7.53 (m, 2H, H-2″, H-6″), 7.34–7.21 (m, 3H, H-3″, H-4″, H-5″), 6.02 (d, *J*_1,2_ = 5.1 Hz, 1H, H-1), 4.42 (dd, *J*_2,3_ = 11.3, *J*_2,1_ = 5.1 Hz, 1H, H-2), 4.27 (brt, *J*_5,6*a*_ = *J*_5,6*b*_ = 6.1 Hz, 1H, H-5), 3.99 (dd, *J*_4,3_ = 3.2, *J*_4,5_ = 1.2 Hz, 1H, H-4), 3.85 (dd, *J*_3,2_ = 11.3, *J*_3,4_ = 3.1 Hz, 1H, H-3), 3.78, 3.75 (part AX of ABX system, *J_AB_* = 11.5 Hz, *J_AX_* = 5.6 Hz, 1H, H-6a); 3.72, 3.69 (part BX of ABX system, *J_BA_* = 11.5 Hz, *J_BX_* = 6.5 Hz, 1H, H-6b). **^13^C NMR** (101 MHz, MeOD) δ 159.5 (q, *J* = 37.6 Hz, CO), 135.8 (C-2″, C-6″), 130.4 (C-1″), 130.2 (C-3″, C-5″), 128.9 (C-4″), 117.4 (q, *J* = 286.7 Hz, CF_3_), 88.4 (C-1), 75.3 (C-5), 69.9 (C-4), 69.5 (C-3), 62.2 (C-6), 53.7 (C-2). HRMS (ESI) *m*/*z*: calcd for C_14_H_16_F_3_NO_5_Se [M+H]^+^ 416.0219; found 416.0244.

Phenyl 3,4,6-tri-*O*-acetyl-2-benzamido-2-deoxy-1-seleno-α-d-galactopyranoside (**7**)

**Procedure b**: Starting material: **2**. The reaction was followed by TLC (Rf = 0.45, EtOAc/Hex 3:5). Purification was performed by silica gel column chromatography (1:4 EtOAc/Hex). Yield: 69%. **Procedure d**: Starting material: **2**. Yield: 66%; LC–MS (high-pH method): Rt = 1.20 min; *m*/*z* = 550.000 [M+H]^+^; yellow syrup; ${\left[\alpha \right]}_{D}^{20}$ = +81.5 (*c* 0.92 CH_2_Cl_2_); **^1^H NMR** (400 MHz, CDCl_3_) δ 7.75 (d, 2H, H-2″, H-6″), 7.56–7.51 (m, 3H, H-4″, H-2′, H-6′), 7.46 (t, *J*_2″,3″_ = 7.4 Hz, 2H, H-3″, H-5″), 7.28–7.20 (m, 3H, H-3′, H-4′, H-6′), 6.49 (d, *J*_*NH*,2_ = 8.3 Hz, 1H, NH), 6.20 (d, *J*_1,2_ = 5.0 Hz, 1H, H-1), 5.51 (d, *J*_4,3_ = 2.1 Hz, 1H, H-4), 5.21 (dd, *J*_3,2_ = 11.6 Hz, *J*_3,4_ = 3.2 Hz, 1H, H-3), 4.96 (ddd, *J*_2,3_ = 11.6 Hz, *J*_2,*NH*_ = 8.3 Hz, *J*_2,1_ = 5.0 Hz, 1H, H-2), 4.70 (brt, *J*_5,6*a*_ = *J*_5,6*b*_ = 6.3 Hz, 1H, H-5), 4.21, 4.18 (part AX of ABX system, *J_AB_* = 11.4 Hz, *J_AX_* = 6.0 Hz, 1H, H-6a); 4.12, 4.09 (part BX of ABX system, *J_BA_* = 11.3 Hz, *J_BX_* = 7.0 Hz, 1H, H-6b), 2.19 (s, 3H, CH_3_), 2.02 (s, 3H, CH_3_), 2.01 (s, 3H, CH_3_). **^13^C NMR** (101 MHz, CDCl_3_) δ 171.7, 170.5, 170.3, 167.4 (4 × CO), 134.3 (C-2′, C-6′), 133.6 (C-1″), 132.2 (C-4″), 129.4 (C-3′, C-5′), 128.9 (C-3″, C-5″), 128.3 (C-4′), 128.1 (C-1′), 127.2 (C-2″, C-6″), 88.2 (C-1), 69.9 (C-5), 69.7 (C-3), 67.2 (C-4), 61.9 (C-6), 50.1 (C-2), 21.0, 20.9, 20.8 (3 × CH_3_). HRMS (ESI) *m*/*z*: calcd for C_25_H_27_NO_8_Se [M+H]^+^ 550.0977; found 550.1001.

Phenyl 2-benzamido-2-deoxy-1-seleno-α-d-galactopyranoside (**8**)

**Procedure f**: Starting material: **7**. Yield: 99%; LC–MS (high-pH method): Rt = 0.83 min; *m*/*z* = 424.000 [M+H]^+^; yellow syrup; ${\left[\alpha \right]}_{D}^{20}$ = +118.3° (*c* 0.54 MeOH); ^**1**^**H NMR** (400 MHz, MeOD) δ 7.83 (d, *J* = 7.6 Hz, 2H, H-2′, H-6′), 7.53 (t, *J* = 7.3 Hz, 3H, H-2″, H-6″, H-4′), 7.45 (t, *J* = 7.5 Hz, 2H, H-3′, H-5′), 7.20 (d, *J* = 7.6 Hz, 3H, H-3″, H-4″, H-5″), 6.21 (d, *J*_1,2_ = 5.1 Hz, 1H, H-1), 4.61 (dd, *J*_2,3_ = 11.1, *J*_2,1_ = 5.2 Hz, 1H, H-2), 4.30 (t, *J*_5,6a_ = *J*_5,6b_ = 5.9 Hz, 1H, H-5), 4.03 (d, *J*_4,3_ =2.6 Hz, 1H, H-4), 3.91 (dd, *J*_3,2_ = 11.4, *J*_3,4_ = 2.8 Hz, 1H, H-3), 3.80, 3.78 (part AX of ABX system, *J_AB_* = 11.4 Hz, *J_AX_* = 5.7 Hz, 1H, H-6a); 3.73, 3.71 (part BX of ABX system, *J_BA_* = 11.4 Hz, *J_BX_* = 6.5 Hz, 1H, H-6b). **^13^C NMR** (101 MHz, MeOD) δ 174.0 (CO), 136.3 (C-1′), 135.7 (C-2″, C-6″), 134.8 (C-1″), 132.8 (C-4″), 130.4 (C-3″, C-5″), 129.5 (C-3′, C-5′), 128.6 (C-4′), 128.5 (C-2′,C-6′), 89.7 (C-1), 75.3 (C-5), 70.0 (C-3), 69.9 (C-4), 62.3 (C-6), 53.6 (C-2). HRMS (ESI) *m*/*z*: calcd for C_19_H_21_NO_5_Se [M+H]^+^ 424.0659; found 424.0680.

Phenyl 3,4,6-tri-*O*-acetyl-2-deoxy-2-(4-methylbenzamido)-1-seleno-α-d-galactopyranoside (**9**)

**Procedure d**: Starting material: **2**. Yield: 42%; LC–MS (high-pH method): Rt = 1.26 min; *m*/*z* = 564.000 [M+H]^+^; yellow syrup; ${\left[\alpha \right]}_{D}^{20}$ = +67.7 (*c* 2.32 CH_2_Cl_2_); **^1^H NMR** (400 MHz, CDCl_3_) δ 7.67 (d, *J* = 7.9 Hz, 2H, H-2′, H-6′), 7.53 (brd, *J* = 7.5 Hz, 2H, H-3″, H-5″), 7.29–7.20 (m, 5H, H-3′, H-5′, H-2″, H-4″, H-6″), 6.55 (d, *J*_*NH*,2_ = 8.2 Hz, 1H, NH), 6.21 (d, *J*_1,2_ = 5.0 Hz, 1H, H-1), 5.53 (d, *J*_4,3_ = 3.2 Hz, 1H, H-4), 5.23 (dd, *J*_3,2_ = 11.6 Hz, *J*_3,4_ = 3.2 Hz, 1H, H-3), 4.96 (ddd, *J*_2,3_ = 11.6 Hz, *J*_2,*NH*_ = 8.2 Hz, *J*_2,1_ = 5.0 Hz, 1H, H-2), 4.73 (brt, *J*_5,6*a*_ = *J*_5,6*b*_ = 6.7 Hz, 1H, H-5), 4.20, 4.17 (part AX of ABX system, *J_AB_* = 11.4 Hz, *J_AX_* = 5.9 Hz, 1H, H-6a); 4.11, 4.08 (part BX of ABX system, *J_BA_* = 11.4 Hz, *J_BX_* = 7.0 Hz, 1H, H-6b), 2.40 (s, 3H, PhCH_3_), 2.19 (s, 3H, CH_3_), 2.00 (s, 3H, CH_3_), 1.99 (s, 3H, CH_3_). **^13^C NMR** (101 MHz, CDCl_3_) δ 171.5, 170.4, 170.3, 167.2 (4 × CO), 142.6 (C-4′), 134.3 (C-2′, C-6′), 130.7 (C-1′), 129.4 (C-2″, C-6″), 129.3 (C-3′, C-5′), 128.1 (C-4″), 127.9 (C-1″), 127.1 (C-3″, C-5″), 88.1 (C-1), 69.8 (C-5), 69.6 (C-3), 67.1 (C-4), 61.7 (C-6), 49.8 (C-2), 21.5 (PhCH_3_), 20.8, 20.7, 20.6 (3 × CH_3_). HRMS (ESI) *m*/*z*: calcd for C_26_H_29_NO_8_Se [M+H]^+^ 564.1133; found 564.1164.

Phenyl 2-deoxy-2-(4-methylbenzamido)-1-seleno-α-d-galactopyranoside (**10**)

**Procedure f**: Starting material: **9**. Yield: 98%; LC–MS (high-pH method): Rt = 0.90 min; *m*/*z* = 438.000 [M+H]^+^; yellow syrup; ${\left[\alpha \right]}_{D}^{20}$ = +75.2° (*c* 0.53 MeOH); ^**1**^**H NMR** (400 MHz, MeOD) δ 7.78 (d, *J* = 8.1 Hz, 2H, H-2′, H-6′), 7.61–7.55 (m, 2H, H-2″, H-6″), 7.31 (d, *J* = 7.9 Hz, 2H, H-3′, H-5′), 7.28–7.17 (m, 3H, H-3″, H-4″, H-5″), 6.22 (d, *J*_1,2_ = 5.1 Hz, 1H, H-1), 4.62 (dd, *J*_2,3_ = 11.3, *J*_2,1_ = 5.1 Hz, 1H, H-2), 4.31 (brt, *J*_5,6a_ = *J*_5,6b_ = 6.0 Hz, 1H, H-5), 4.05 (dd, *J*_4,5_ =1.1 Hz, *J*_4,3_ =3.2 Hz, 1H, H-4), 3.92 (dd, *J*_3,2_ = 11.3 Hz, *J*_3,4_ = 3.2 Hz, 1H, H-3), 3.81, 3.78 (part AX of ABX system, *J_AB_* = 11.5 Hz, *J_AX_* = 5.6 Hz, 1H, H-6a); 3.75, 3.72 (part BX of ABX system, *J_BA_* = 11.4 Hz, *J_BX_* = 6.5 Hz, 1H, H-6b). **^13^C NMR** (101 MHz, MeOD) δ 171.0 (CO), 143.5 (C-4′), 138.1 (C-1′), 135.7 (C-2″, C-6″), 135.6 (C-1″), 132.8 (C-4″), 130.1 (C-3′, C-5′), 130.0 (s, C-3″, C-5″), 128.6 (C-2′, C-6′), 89.8 (C-1), 75.3 (C-5), 70.2 (C-3), 70.0 (C-4), 62.3 (C-6), 53.6 (C-2), 21.5 (CH_3_). HRMS (ESI) *m*/*z*: calcd for C_20_H_23_NO_5_Se [M+H]^+^ 438.0815; found 438.0841.

Phenyl 3,4,6-tri-*O*-acetyl-2-deoxy-2-(4-fluorobenzamido)-1-seleno-α-d-galactopyranoside (**11**)

**Procedure d**: Starting material: **2**. Yield: 43%; LC–MS (high-pH method): Rt = 1.22 min; *m*/*z* = 568.000 [M+H]^+^; yellow syrup; ${\left[\alpha \right]}_{D}^{20}$ = +90.8 (*c* 2.07 CH_2_Cl_2_); **^1^H NMR** (400 MHz, CDCl_3_) δ 7.85–7.75 (m, 2H, H-2′, H-6′), 7.54 (d, *J* = 7.1 Hz, 2H, H-2″, H-6″), 7.31–7.19 (m, 3H, H-3″, H-4″, H-5″), 7.13 (t, *J* = 8.6 Hz, 2H, H-3′, H-5′), 6.73 (d, *J*_*NH*,2_ = 8.0 Hz, 1H, NH), 6.25 (d, *J*_1,2_ = 5.0 Hz, 1H, H-1), 5.55 (dd, *J*_4,3_ = 3.3 Hz, *J*_4,5_ = 1.3 Hz, 1H, H-4), 5.28 (dd, *J*_3,2_ = 11.5 Hz, *J*_3,4_ = 3.1 Hz, 1H, H-3), 4.95 (ddd, *J*_2,3_ = 11.7 Hz, *J*_2,*NH*_ = 8.0 Hz, *J*_2,1_ = 5.0 Hz, 1H, H-2), 4.77 (brt, *J*_5,6*a*_ = *J*_5,6*b*_ = 6.6 Hz, 1H, H-5), 4.20, 4.17 (part AX of ABX system, *J*_AB_ = 11.3 Hz, *J*_AX_ = 6.0 Hz, 1H, H-6a); 4.11, 4.08 (part BX of ABX system, *J*_BA_ = 11.1 Hz, *J*_BX_ = 7.0 Hz, 1H, H-6b), 2.19 (s, 3H, CH_3_), 1.99 (s, 3H, CH_3_), 1.95 (s, 3H, CH_3_). **^13^C NMR** (101 MHz, CDCl_3_) δ 171.7, 170.4, 170.3, 166.3 (4 × CO), 165.0 (d, *J* = 253.1 Hz, C-4′), 134.3 (C-2″, C-6″), 129.7 (d, *J* = 3.1 Hz, C-1′), 129.5 (d, *J* = 9.1 Hz, C-2′, C-6′), 129.3 (C-3″, C-5″), 128.2 (C-4″), 127.8 (C-1″), 115.8 (d, *J* = 22.0 Hz, C-3′, C-5′), 87.7 (C-1), 69.8 (C-5), 69.6 (C-3), 67.1 (C-4), 61.7 (C-6), 50.0 (C-2), 20.8, 20.7, 20.6 (3 × CH_3_). HRMS (ESI) *m*/*z*: calcd for C_25_H_26_FNO_8_Se [M+H]^+^ 568.0882; found 568.0919.

Phenyl 2-deoxy-2-(4-fluorobenzamido)-1-seleno-α-d-galactopyranoside (**12**)

**Procedure f**: Starting material: **11**. Yield: 94%; LC–MS (high-pH method): Rt = 0.87 min; *m*/*z* = 442.000 [M+H]^+^; yellow syrup; **^1^H NMR** (400 MHz, MeOD) δ 7.94–7.88 (m, 2H, H-2′, H-6′), 7.57–7.53 (m, 2H, H-2″, H-6″), 7.27–7.16 (m, 5H, H-3′, H-5′, H-3″, H-4″, H-5″), 6.21 (d, *J*_1,2_ = 5.1 Hz, 1H, H-1), 4.60 (dd, *J*_2,3_ = 11.3 Hz, *J*_2,1_ = 5.1 Hz, 1H, H-2), 4.29 (td, *J*_5,6a_ = *J*_5,6b_ = 6.1 Hz, *J*_5,4_ = 1.3 Hz, 1H, H-5), 4.03 (dd, *J*_4,3_ = 3.2 Hz, *J*_4,5_ = 1.2 Hz, 1H, H-4), 3.92 (dd, *J*_3,2_ = 11.3 Hz, *J*_3,4_ = 3.1 Hz, 1H, H-3), 3.80, 3.77 (part AX of ABX system, *J_AB_* = 11.4 Hz, *J_AX_* = 5.7 Hz, 1H, H-6a); 3.74, 3.71 (part BX of ABX system, *J_BA_* = 11.4 Hz, *J_BX_* = 6.5 Hz, 1H, H-6b). **^13^C NMR** (101 MHz, MeOD) δ 168.6 (CO), 164.9 (d, *J* = 250.4 Hz, C-4′), 134.3 (C-2″, C-6″), 130.5 (d, *J* = 3.1 Hz, C-1′), 129.8 (d, *J* = 9.1 Hz, C-2′, C-6′), 129.1 (C-1″), 128.8 (C-3″, C-5″), 127.4 (C-4″), 115.0 (d, *J* = 22.2 Hz, C-3′, C-5′), 88.1 (C-1), 73.9 (C-5), 68.7 (C-3), 68.5 (C-4), 60.9 (C-6), 52.3 (C-2). HRMS (ESI) *m*/*z*: calcd for C_19_H_20_FNO_5_Se [M+H]^+^ 442.0565; found 442.0593.

Phenyl 3,4,6-tri-*O*-acetyl-2-deoxy-2-[4-(trifluoromethyl)benzamido]-1-seleno-α-d-galactopyranoside (**13**)

**Procedure d**: Starting material: **2**. Yield: 56%; yellow syrup; ${\left[\alpha \right]}_{D}^{20}$ = +63.9 (*c* 0.83 CH_2_Cl_2_); **^1^H NMR** (400 MHz, CDCl_3_) δ 7.89 (d, *J* = 8.0 Hz, 2H, Ph), 7.75 (d, *J* = 8.3 Hz, 2H, Ph), 7.58–7.53 (m, 2H, Ph), 7.32–7.23 (m, 3H, Ph), 6.71 (d, *J*_NH,2_ = 8.1 Hz, 1H, NH), 6.25 (d, *J*_1,2_ = 5.0 Hz, 1H, H-1), 5.56 (d, *J*_4,3_ = 2.7 Hz, 1H, H-4), 5.27 (dd, *J*_3,2_ = 11.6 Hz, *J*_3,4_ = 3.3 Hz, 1H, H-3), 4.96 (ddd, *J*_2,3_ = 11.6 Hz, *J*_2,NH_ = 8.1 Hz, *J*_2,1_ = 5.0 Hz, 1H, H-2), 4.75 (brt, *J*_5,6a_ = *J*_5,6b_ = 6.6 Hz, 1H, H-5), 4.23, 4.20 (part AX of ABX system, *J*_AB_ = 11.4 Hz, *J*_AX_ = 5.8 Hz, 1H, H-6a); 4.15, 4.12 (part BX of ABX system, *J*_BA_ = 11.4 Hz, *J*_BX_ = 7.0 Hz, 1H, H-6b), 2.22 (s, 3H, CH_3_), 2.04 (s, 3H, CH_3_), 2.02 (s, 3H, CH_3_). **^13^C NMR** (101 MHz, CDCl_3_) δ 171.9, 170.5, 170.3, 166.1 (4 × CO), 134.3 (Ph), 133.9 (q, *J* = 33.3 Hz, C-4′), 129.5 (Ph), 128.4 (Ph), 127.9 (Ph), 127.7 (Ph), 126.0 (q, *J* = 3.8 Hz, C-3′, C-5′), 123.8 (q, *J* = 272.8 Hz, CF_3_), 87.7 (C-1), 70.0 (C-5), 69.7 (C-3), 67.1 (C-4), 61.8 (C-6), 50.4 (C-2), 21.0, 20.9, 20.8 (3 × CH_3_). HRMS (ESI) *m*/*z*: calcd for C_26_H_26_F_3_NO_8_Se [M+H]^+^ 618.0850; found 618.0891.

Phenyl 2-deoxy-2-[4-(trifluoromethyl)benzamido]-1-seleno-α-d-galactopyranoside (**14**)

**Procedure f**: Starting material: **13**. Yield: 97%; LC–MS (high-pH method): Rt = 1.00 min; *m*/*z* = 492.000 [M+H]^+^; yellow syrup; ${\left[\alpha \right]}_{D}^{20}$ = +157.6 (*c* 0.30 MeOH); ^**1**^**H NMR** (400 MHz, MeOD) δ 7.91 (d, *J* = 8.1 Hz, 2H, H-2′, H-6′), 7.69 (d, *J* = 8.2 Hz, 2H, H-3′, H-5′), 7.48–7.44 (m, 2H, H-3″, H-5″), 7.15–7.08 (m, 3H, H-2″, H-4″, H-6″), 6.12 (d, *J*_1,2_ = 5.1 Hz, 1H, H-1), 4.52 (dd, *J*_2,3_ = 11.3 Hz, *J*_2,1_ = 5.1 Hz, 1H, H-2), 4.19 (brt, *J*_5,6a_ = *J*_5,6b_ = 6.1 Hz, 1H, H-5), 3.94 (dd, *J*_4,3_ = 3.2 Hz, *J*_4,5_ = 1.3 Hz, 1H, H-4), 3.83 (dd, *J*_3,4_ = 3.2 Hz, *J*_3,2_ = 11.3 Hz, 1H, H-3), 3.70, 3.67 (part AX of ABX system, *J_AB_* = 11.4 Hz, *J_AX_* = 5.8 Hz, 1H, H-6a); 3.64, 3.61 (part BX of ABX system, *J_BA_* = 11.4 Hz, *J_BX_* = 6.5 Hz, 1H, H-6b). **^13^C NMR** (101 MHz, MeOD) δ 168.3 (CO), 138.1 (d, *J* = 1.1 Hz, C-1′), 134.2 (C-3″, C-5″), 132.7 (q, *J* = 32.5 Hz, C-4′), 129.2 (C-1″), 128.7 (C-2″, C-6″), 127.9 (C-2′, C-6′), 127.3 (C-4″), 125.1 (q, *J* = 3.8 Hz, C-3′, C-5′), 124.0 (q, *J* = 270.8 Hz, CF_3_), 88.0 (C-1), 73.9 (C-5), 68.7 (C-3), 68.6 (C-4), 60.9 (C-6), 52.4 (C-2). HRMS (ESI) *m*/*z*: calcd for C_20_H_20_F_3_NO_5_Se [M+H]^+^ 492.0533; found 492.0566.

Phenyl 3,4,6-tri-*O*-acetyl-2-deoxy-2-(3-fluoro-5-methylbenzamido)-1-seleno-α-d-galactopyranoside (**15**)

**Procedure d**: Starting material: **2**. Yield: 61%; LC–MS (high-pH method): Rt = 1.29 min; *m*/*z* = 582.000 [M+H]^+^; yellow syrup; ${\left[\alpha \right]}_{D}^{20}$ = +78.2 (*c* 2.02 CH_2_Cl_2_); **^1^H NMR** (400 MHz, CDCl_3_) δ 7.54 (d, J = 7.8 Hz, 2H, H-2″, H-6″), 7.34 (s, 1H, H-6′), 7.31–7.21 (m, 4H, H-2′, H-3″, H-4″, H-5″), 7.05 (d, *J* = 9.4 Hz, 1H, H-4′), 6.61 (d, *J*_*NH*,2_ = 8.1 Hz, 1H, NH), 6.22 (d, *J*_1,2_ = 5.1 Hz, 1H, H-1), 5.54 (dd, *J*_4,3_ = 3.4 Hz, *J*_4,5_ = 1.4 Hz, 1H, H-4), 5.25 (dd, *J*_3,2_ = 11.6 Hz, *J*_3,4_ = 3.2 Hz, 1H, H-3), 4.94 (ddd, *J*_2,3_ = 11.6 Hz, *J*_2,*NH*_ = 8.1 Hz, *J*_2,1_ = 5.1 Hz, 1H, H-2), 4.74 (brt, *J*_5,6*a*_ = *J*_5,6*b*_ = 6.5 Hz, 1H, H-5), 4.20, 4.17 (part AX of ABX system, *J*_AB_ = 11.3 Hz, *J*_AX_ = 6.0 Hz, 1H, H-6a); 4.11, 4.08 (part BX of ABX system, *J*_BA_ = 11.3 Hz, *J*_BX_ = 7.0 Hz, 1H, H-6b), 2.42 (s, 3H, Ph-CH_3_), 2.19 (s, 3H, CH_3_), 2.02 (s, 3H, CH_3_), 1.97 (s, 3H, CH_3_). **^13^C NMR** (101 MHz, CDCl_3_) δ 171.6, 170.4, 170.2 (3 × CO), 166.3 (d, *J* = 2.7 Hz, NHCO), 162.7 (d, *J* = 247.2 Hz, C-3′), 141.1 (d, *J* = 7.7 Hz, C-5′), 135.4 (d, *J* = 7.3 Hz, C-1′), 134.3 (C-2″, C-6″), 129.3 (C-5″, C-3″), 128.2 (C-4″), 127.8 (C-1″), 123.4 (d, *J* = 2.6 Hz, C-6′), 119.6 (d, *J* = 21.2 Hz, C-4′), 111.4 (d, *J* = 23.2 Hz, C-2′), 87.7 (C-1), 69.8 (C-3), 69.5 (C-5), 67.1 (C-4), 61.7 (C-6), 50.0 (C-2), 21.4 (d, *J* = 1.8 Hz, Ph-CH_3_), 20.8, 20.7, 20.6 (3 × CH_3_). HRMS (ESI) *m*/*z*: calcd for C_26_H_28_FNO_8_Se [M+H]^+^ 582.1039; found 582.1078.

Phenyl 2-deoxy-2-(3-fluoro-5-methylbenzamido)-1-seleno-α-d-galactopyranoside (**16**)

**Procedure f**: Starting material: **15**. Yield: 87%; LC–MS (high-pH method): Rt = 0.93 min; *m*/*z* = 456.000 [M+H]^+^; yellow syrup; ${\left[\alpha \right]}_{D}^{20}$ = +99.8 (*c* 0.53 MeOH); ^**1**^**H NMR** (400 MHz, MeOD) δ 7.46 (d, *J* = 6.9 Hz, 2H, H-2″, H-6″), 7.41 (s, 1H, H-2′), 7.27 (d, *J* = 9.4 Hz, 1H, H-6′), 7.12 (d, *J* = 7.4 Hz, 3H, H-3″, H-4″, H-5″), 7.03 (d, *J* = 9.5 Hz, 1H, H-4′), 6.10 (d, *J*_1,2_ = 5.1 Hz, 1H, H-1), 4.48 (dd, *J*_2,3_ = 11.2 Hz, *J*_2,1_ = 5.1 Hz, 1H, H-2), 4.18 (brt, *J*_5,6a_ = *J*_5,6b_ = 6.2 Hz, 1H, H-5), 3.93 (d, *J*_4,3_ = 3.2 Hz, 1H, H-4), 3.82 (dd, *J*_3,2_ = 11.2 Hz, *J*_3,4_ = 3.2 Hz, 1H, H-3), 3.69, 3.67 (part AX of ABX system, *J_AB_* = 11.5 Hz, *J_AX_* = 5.7 Hz, 1H, H-6a); 3.63, 3.60 (part BX of ABX system, *J_BA_* = 11.5 Hz, *J_BX_* = 6.4 Hz, 1H, H-6b), 2.33 (s, 3H, CH_3_). **^13^C NMR** (101 MHz, MeOD) δ 169.7 (d, *J* = 2.7 Hz, CO), 164.0 (d, *J* = 245.2 Hz, C-3′), 142.4 (d, *J* = 7.8 Hz, C-1′), 137.7 (d, *J* = 7.7 Hz, C-5′), 135.7 (C-2″, C-6″), 130.7 (C-1″), 130.1 (C-3″, C-5″), 128.6 (C-4″), 125.0 (d, *J* = 2.6 Hz, C-2′), 119.9 (d, *J* = 21.4 Hz, C-4′), 112.5 (d, *J* = 23.4 Hz, C-6′), 89.5 (C-1), 75.3 (C-5), 70.0 (C-3), 69.9 (C-4), 62.3 (C-6), 53.7 (C-2), 21.3 (d, *J* = 1.8 Hz, CH_3_). HRMS (ESI) *m*/*z*: calcd for C_20_H_22_FNO_5_Se [M+H]^+^ 456.0721; found 456.0749.

Phenyl 3,4,6-tri-*O*-acetyl-2-deoxy-2-(4-fluoro-5-methylbenzamido)-1-seleno-α-d-galactopyranoside (**17**)

**Procedure d**: Starting material: **2**. Yield: 73%; LC–MS (high-pH method): Rt = 1.28 min; *m*/*z* = 582.000 [M+H]^+^; yellow syrup; ${\left[\alpha \right]}_{D}^{20}$ = +110.8 (*c* 2.04 CH_2_Cl_2_); **^1^H NMR** (400 MHz, CDCl_3_) δ 7.66 (dd, *J* = 7.3, 2.3 Hz, 1H, H-2′), 7.58 (ddd, *J* = 7.7, 4.8, 2.4 Hz, 1H, H-6′), 7.54 (d, *J* = 6.6 Hz, 2H, H-2″, H-6″), 7.32–7.19 (m, 3H, H-3″, H-4″, H-5″), 7.05 (t, *J* = 8.8, 8.9 Hz, 1H, H-5′), 6.72 (d, *J*_NH,2_ = 8.0 Hz, 1H, NH), 6.26 (d, *J*_1,2_ = 5.0 Hz, 1H, H-1), 5.56 (dd, *J*_4,3_ = 3.3, *J*_4,5_ = 1.4 Hz, 1H, H-4), 5.28 (dd, *J*_3,2_ = 11.7 Hz, *J*_3,4_ = 3.2 Hz, 1H, H-3), 4.95 (ddd, *J*_2,3_ = 11.6, *J*_2,NH_ = 8.0, *J*_2,1_ = 5.0 Hz, 1H, H-2), 4.78 (brt, *J*_5,6a_ = *J*_5,6b_ = 6.4 Hz, 1H, H-5), 4.19, 4.17 (part AX of ABX system, *J*_AB_ = 11.4, *J*_AX_ = 6.0 Hz, 1H, H-6a); 4.10, 4.08 (part BX of ABX system, *J*_BA_ = 11.4, *J*_BX_ = 7.0 Hz, 1H, H-6b), 2.32 (d, *J*_CH3-F_ = 2.0 Hz, 3H, Ph-CH_3_), 2.19 (s, 3H, CH_3_), 1.99 (s, 3H, CH_3_), 1.95 (s, 3H, CH_3_). **^13^C NMR** (101 MHz, CDCl_3_) δ 171.6, 170.4, 170.3, 166.6 (4 × CO), 163.2 (d, *J* = 251.4 Hz, C-4′), 134.3 (C-2″, C-6″), 131.0 (d, *J* = 6.6 Hz, C-2′), 129.4 (d, *J* = 3.6 Hz, C-1′), 128.3 (C-3″, C-5″), 128.1 (C-4″), 127.8 (C-1″), 126.5 (d, *J* = 9.5 Hz, C-6′), 115.3 (d, *J* = 23.5 Hz, C-5′), 87.7 (C-1), 69.7 (C-5), 69.6 (C-3), 67.0 (C-4), 61.7 (C-6), 49.9 (C-2), 20.8, 20.7, 20.6 (3 × CH_3_), 14.6 (s, Ph-CH_3_). HRMS (ESI) *m*/*z*: calcd for C_26_H_28_FNO_8_Se [M+H]^+^ 582.1039; found 582.1067.

Phenyl 2-deoxy-2-(4-fluoro-5-methylbenzamido)-1-seleno-α-d-galactopyranoside (**18**)

**Procedure f**: Starting material: **17**. Yield: 93%; LC–MS (high-pH method): Rt = 0.94 min; *m*/*z* = 456.000 [M+H]^+^; yellow syrup; ${\left[\alpha \right]}_{D}^{20}$ = +97.8 (*c* 0.67 MeOH); **^1^H NMR** (400 MHz, MeOD) δ 7.79–7.75 (m, 1H, H-6′), 7.72 (ddd, *J* = 7.7, 4.9, 2.4 Hz, 1H, H-3′), 7.57–7.53 (m, 2H, H-2″, H-6″), 7.27–7.17 (m, 3H, H-3″, H-4″, H-5″), 7.11 (dd, *J* = 9.5, 8.5 Hz, 1H, H-2′), 6.21 (d, *J*_1,2_ = 5.1 Hz, 1H, H-1), 4.59 (dd, *J*_2,3_ = 11.3 Hz, *J*_2,1_ = 5.1 Hz, 1H, H-2), 4.29 (brt, *J*_5,6a_ = *J*_5,6b_ = 6.1 Hz, 1H, H-5), 4.04 (d, *J*_4,3_ = 3.2 Hz, 1H, H-4), 3.93 (dd, *J*_3,2_ = 11.3 Hz, *J*_3,4_ = 3.2 Hz, 1H, H-3), 3.80, 3.77 (part AX of ABX system, *J_AB_* = 11.4 Hz, *J_AX_* = 5.7 Hz, 1H, H-6a); 3.74, 3.71 (part BX of ABX system, *J_BA_* = 11.4 Hz, *J_BX_* = 6.5 Hz, 1H, H-6b), 2.32 (d, *J*_*CH*3,*F*_ = 2.0 Hz, 3H, CH_3_). **^13^C NMR** (101 MHz, MeOD) δ 169.2 (CO), 163.8 (d, *J* = 249.3 Hz, C-4′), 134.8 (C-2″, C-6″), 131.4 (d, *J* = 5.9 Hz, C-6′), 130.8 (d, *J* = 3.5 Hz, C-1′), 129.8 (C-1″), 129.2 (C-3″, C-5″), 127.8 (C-4″), 127.6 (d, *J* = 9.1 Hz, C-3′), 125.3 (d, *J* = 17.9 Hz, C-5′), 115.1 (d, *J* = 23.2 Hz, C-2′), 88.7 (C-1), 74.4 (C-5), 69.2 (C-3), 69.1 (C-4), 61.5 (C-6), 52.8 (C-2), 13.6 (d, *J* = 3.7 Hz, CH_3_). HRMS (ESI) *m*/*z*: calcd for C_20_H_22_FNO_5_Se [M+H]^+^ 456.0721; found 456.0743.

Phenyl 3,4,6-tri-*O*-acetyl-2-deoxy-2-[6-(trifluoromethyl)nicotinamido]-1-seleno-α-d-galactopyranoside (**19**)

**Procedure d**: Starting material: **2**. Yield: 49%; yellow syrup; **^1^H NMR** (400 MHz, CDCl_3_) δ 9.11 (d, *J* = 1.8 Hz, 1H, H-2′), 8.28 (dd, *J* = 8.1, 1.8 Hz, 1H, H-4′), 7.79 (d, *J* = 8.1 Hz, 1H, H-5′), 7.55 (d, *J* = 8.1 Hz, 2H, H-2″, H-6″), 7.33–7.20 (m, 4H, H-3″, H-4″, H-5″, NH), 6.30 (d, *J*_1,2_ = 5.0 Hz, 1H, H-1), 5.60 (dd, *J*_4,3_ = 3.3 Hz, *J*_4,5_ = 1.4 Hz, 1H, H-4), 5.37 (dd, *J*_3,2_ = 11.7, *J*_3,4_ = 3.3 Hz, 1H, H-3), 4.97 (ddd, *J*_2,3_ = 11.6 Hz, *J*_2,*NH*_ = 7.8 Hz, *J*_2,1_ = 5.0 Hz, 1H, H-2), 4.84 (brt, *J*_5,6*a*_ = *J*_5,6*b*_ = 6.6 Hz, 1H, H-5), 4.19, 4.17 (part AX of ABX system, *J*_AB_ = 11.3, *J*_AX_ = 6.2 Hz, 1H, H-6a); 4.11, 4.08 (part BX of ABX system, *J*_BA_ = 11.3, *J*_BX_ = 6.8 Hz, 1H, H-6b), 2.20 (s, 3H, CH_3_), 2.02 (s, 3H, CH_3_), 1.88 (s, 3H, CH_3_). ^**13**^**C NMR** (101 MHz, CDCl_3_) δ 170.1, 168.4, 168.4, 162.6 (4 × CO), 148.7 (q, *J* = 35.7 Hz, C-6′), 146.8 (C-2′), 134.9 (C-4′), 132.5 (C-2″, C-6″), 129.9 (C-3′), 127.5 (C-3″, C-5″), 126.5 (C-4″), 125.5 (C-1″), 119.2 (q, *J* = 275.1 Hz, CF_3_), 118.5 (q, *J* = 2.6 Hz, C-5′), 85.2 (C-1), 67.9 (C-5), 67.7 (C-3), 65.0 (C-4), 59.7 (C-6), 48.3 (C-2), 18.9, 18.8, 18.6, (3 × CH_3_). LC–MS (high-pH method): Rt = 1.25 min; *m*/*z* = 619.000 [M+H]^+^.

Phenyl 2-deoxy-2-[6-(trifluoromethyl)nicotinamido]-1-seleno-α-d-galactopyranoside (**20**)

**Procedure f**: Starting material: **19**. Yield: 93%; LC–MS (high-pH method): Rt = 0.90 min; *m*/*z* = 493.000 [M+H]^+^; yellow syrup; ${\left[\alpha \right]}_{D}^{20}$ = +107.9 (*c* 0.79 MeOH); **^1^H NMR** (400 MHz, MeOD) δ 9.00 (d, *J* = 2.1 Hz, 1H, H-2′), 8.31 (dd, *J* = 8.2, 2.2 Hz, 1H, H-4′), 7.84 (dd, *J* = 8.2, 0.9 Hz, 1H, H-5′), 7.52–7.41 (m, 2H, H-2″, H-6″), 7.18–7.05 (m, 3H, H-3″, H-4″, H-5″), 6.12 (d, *J*_1,2_ = 5.1 Hz, 1H, H-1), 4.53 (dd, *J*_2,3_ = 11.3 Hz, *J*_2,1_ = 5.1 Hz, 1H, H-2), 4.18 (brt, *J*_5,6a_ = *J*_5,6b_ = 6.1 Hz, 1H, H-5), 3.94 (dd, *J*_4,3_ = 3.2 Hz, *J*_4,5_ = 1.2 Hz, 1H, H-4), 3.83 (dd, *J*_3,2_ = 11.3 Hz, *J*_3,4_ = 3.1 Hz, 1H, H-3), 3.70, 3.67 (part AX of ABX system, *J_AB_* = 11.4 Hz, *J_AX_* = 5.7 Hz, 1H, H-6a); 3.64, 3.62 (part BX of ABX system, *J_BA_* = 11.5 Hz, *J_BX_* = 6.5 Hz, 1H, H-6b). **^13^C NMR** (101 MHz, MeOD) δ 166.7 (CO), 150.0 (q, *J* = 34.8 Hz, C-3′), 149.3 (C-2′), 137.8 (C-4′), 134.7 (C-2″, C-6″), 133.8 (C-6′), 129.7 (C-1″), 129.3 (C-3″, C-5″), 127.9 (C-4″), 124.6 (q, J = 272.9 Hz, CF_3_), 120.8 (q, *J* = 2.8 Hz, C-5′), 88.3 (C-1), 74.6 (C-5), 69.2 (C-3), 69.1 (C-4), 61.5 (C-6), 53.0 (C-2). HRMS (ESI) *m*/*z*: calcd for C_19_H_19_F_3_N_2_O_5_Se [M+H]^+^ 493.0485; found 493.0512.

Phenyl 3,4,6-tri-*O*-acetyl-2-deoxy-2-(3-naphthamido)-1-seleno-α-d-galactopyranoside (**21**)

**Procedure d**: Starting material: **2**. Yield: 80%; LC–MS (high-pH method): Rt = 1.31 min; *m*/*z* = 600.000 [M+H]^+^; yellow syrup; ${\left[\alpha \right]}_{D}^{20}$ = +45.7 (*c* 1.29 CH_2_Cl_2_); **^1^H NMR** (400 MHz, CDCl_3_) δ 8.32 (s, 1H, Ph), 8.01–7.77 (m, 4H, Ph), 7.63–7.48 (m, 4H, Ph), 7.32–7.17 (m, 3H, Ph), 7.00 (d, *J*_*NH*,2_ = 8.0 Hz, 1H, NH), 6.33 (d, *J*_1,2_ = 5.0 Hz, 1H, H-1), 5.60 (dd, *J*_4,3_ = 3.3 Hz, *J*_4,5_ = 1.4 Hz, 1H, H-4), 5.36 (dd, *J*_3,2_ = 11.7 Hz, *J*_3,4_ = 3.3 Hz, 1H, H-3), 5.05 (ddd, *J*_2,3_ = 11.7 Hz, *J*_2,*NH*_ = 8.0 Hz, *J*_2,1_ = 5.0 Hz, 1H, H-2), 4.82 (ddd, *J*_5,6*a*_ = 7.2 Hz, *J*_5,6*b*_ = 5.8 Hz, J_5,4_ = 1.4 Hz, 1H, H-5), 4.22, 4.19 (part AX of ABX system, *J*_AB_ = 11.4, *J*_AX_ = 6.0 Hz, 1H, H-6a); 4.13, 4.10 (part BX of ABX system, *J*_BA_ = 11.4, *J*_BX_ = 7.0 Hz, 1H, H-6b), 2.21 (s, 3H, CH_3_), 1.98 (s, 3H, CH_3_), 1.95 (s, 3H, CH_3_). **^13^C NMR** (101 MHz, CDCl_3_) δ 171.7, 170.4, 170.4, 167.6 (4 × CO), 134.9, 134.4, 132.6, 130.7, 129.3, 129.1, 128.6, 128.1, 127.9, 127.8, 126.9, 123.5 (aromatic C), 87.8 (C-1), 69.8 (C-5), 69.7 (C-3), 67.1 (C-4), 61.7 (C-6), 50.0 (C-2), 20.8, 20.8, 20.6 (3 × CH_3_). HRMS (ESI) *m*/*z*: calcd for C_29_H_29_NO_8_Se [M+H]^+^ 600.1134; found 600.1187.

Phenyl 2-deoxy-2-(3-naphthamido)-1-seleno-α-d-galactopyranoside (**22**)

**Procedure f**: Starting material: **21**. Yield: 93%; LC–MS (high-pH method): Rt = 0.98 min; *m*/*z* = 474.000 [M+H]^+^; yellow syrup; ${\left[\alpha \right]}_{D}^{20}$ = −3.0 (*c* 0.24 MeOH); **^1^H NMR** (400 MHz, MeOD) δ 8.43 (d, *J* = 1.5 Hz, 1H, H-10′), 8.04–7.88 (m, 4H, H-9′, H-7′, H-4′, H-2′), 7.64–7.53 (m, 4H, H-6′, H-5′, H-2″, H-6″), 7.28–7.14 (m, 3H, H-3″, H-4″, H-5″), 6.27 (d, *J*_1,2_ = 5.1 Hz, 1H, H-1), 4.67 (dd, *J*_2,3_ = 11.3 Hz, *J*_2,1_ = 5.1 Hz, 1H, H-2), 4.32 (brt, *J*_5,6a_ = *J*_5,6b_ = 6.0 Hz, 1H, H-5), 4.05 (dd, *J*_4,3_ = 3.2 Hz, *J*_4,5_ = 1.2 Hz, 1H, H-4), 3.97 (dd, *J*_3,2_ = 11.3 Hz, *J*_3,4_ = 3.2 Hz, 1H, H-3), 3.81, 3.78 (part AX of ABX system, *J_AB_* = 11.4 Hz, *J_AX_* = 5.7 Hz, 1H, H-6a); 3.75, 3.72 (part BX of ABX system, *J_BA_* = 11.4 Hz, *J_BX_* = 6.5 Hz, 1H, H-6b). **^13^C NMR** (101 MHz, MeOD) δ 171.1 (CO), 135.7, 130.0, 129.3, 129.0, 128.9, 128.8, 128.6, 127.9, 125.1 (aromatic C), 89.7 (C-1), 75.4 (C-5), 70.2 (C-3), 70.0 (C-4), 62.3 (C-6), 53.8 (C-2). HRMS (ESI) *m*/*z*: calcd for C_23_H_23_NO_5_Se [M+H]^+^ 474.0816; found 474.0830.

Phenyl 3,4,6-tri-*O*-acetyl-2-deoxy-2-(4-methoxybenzamido)-1-seleno-α-d-galactopyranoside (**23**)

**Procedure d**: Starting material: **2**. Yield: 41%; LC–MS (high-pH method): Rt = 1.19 min; *m*/*z* = 580.200 [M+H]^+^; yellow syrup; ${\left[\alpha \right]}_{D}^{20}$ = +35.6 (*c* 1.60 CH_2_Cl_2_); **^1^H NMR** (400 MHz, CDCl_3_) δ 7.73 (d, *J* = 8.9 Hz, 2H, H-2′, H-6′), 7.53 (dt, *J* = 6.7, 1.6 Hz, 2H, H-2″, H-6″), 7.31–7.19 (m, 3H, H-3″, H-4″, H-5″), 6.94 (d, *J* = 8.9 Hz, 2H, H-3′, H-5′), 6.48 (d, *J*_*NH*,2_ = 8.2 Hz, 1H, NH), 6.21 (d, *J*_1,2_ = 5.0 Hz, 1H, H-1), 5.52 (dd, *J*_4,3_ = 3.3 Hz, *J*_4,5_ = 1.3 Hz, 1H, H-4), 5.23 (dd, *J*_3,2_ = 11.6 Hz, *J*_3,4_ = 3.3 Hz, 1H, H-3), 4.95 (ddd, *J*_2,3_ = 11.6 Hz, *J*_2,*NH*_ = 8.2 Hz, *J*_2,1_ = 5.0 Hz, 1H, H-2), 4.71 (brt, *J*_5,6*a*_ = *J*_5,6*b*_ = 6.5 Hz, 1H, H-5), 4.20, 4.17 (part AX of ABX system, *J_AB_* = 11.4 Hz, *J_AX_* = 5.9 Hz, 1H, H-6a); 4.11, 4.08 (part BX of ABX system, *J_BA_* = 11.4 Hz, *J_BX_* = 7.1 Hz, 1H, H-6b), 3.86 (s, 3H, OCH_3_), 2.19 (s, 3H, CH_3_), 2.00 (s, 6H, 2 × CH_3_). ^**13**^**C NMR** (101 MHz, CDCl_3_) δ 170.8, 169.6, 169.4, 166.0 (4 × CO), 161.8 (C-4′), 133.4 (C-2″, C-6″), 128.5 (C-3″, C-5″), 128.1 (C-2′, C-6′), 127.3 (C-4″), 127.2 (C-1″), 125.0 (C-1′), 113.1 (C-3′, C-5′), 87.3 (C-1), 69.0 (C-5), 68.8 (C-3), 66.3 (C-4), 60.9 (C-6), 54.6 (OCH_3_), 49.1 (C-2), 20.0, 19.9, 19.8 (3 × CH_3_). HRMS (ESI) *m*/*z*: calcd for C_26_H_29_NO_9_Se [M+H]^+^ 580.1082; found 580.1112.

Phenyl 2-deoxy-2-(4-methoxybenzamido)-1-seleno-α-d-galactopyranoside (**24**)

**Procedure f**: Starting material: **23.** Yield: 97%; LC–MS (high-pH method): Rt = 0.84 min; *m*/*z* = 454.000 [M+H]^+^; yellow syrup; ${\left[\alpha \right]}_{D}^{20}$ = +51.2 (*c* 0.41 MeOH); **^1^H NMR** (400 MHz, MeOD) δ 7.88–7.80 (m, 2H, H-2′, H-6′), 7.59–7.52 (m, 2H, H-2″, H-6″), 7.27–7.17 (m, 3H, H-3″, H-4″, H-5″), 7.04–6.96 (m, 2H, H-3′, H-5′), 6.20 (d, *J*_1,2_ = 5.1 Hz, 1H, H-1), 4.59 (dd, *J*_2,3_ = 11.2 Hz, *J*_2,1_ = 5.1 Hz, 1H, H-2), 4.29 (brt, *J*_5,6a_ = *J*_5,6b_ = 6.1 Hz, 1H, H-5), 4.03 (d, *J*_4,3_ = 2.5 Hz, 1H, H-4), 3.90 (dd, *J*_3,2_ = 11.3 Hz, *J*_3,4_ = 3.1 Hz, 1H, H-3), 3.86 (s, 3H, OCH_3_), 3.79, 3.77 (part AX of ABX system, *J_AB_* = 11.5 Hz, *J_AX_* = 5.6 Hz, 1H, H-6a); 3.73, 3.70 (part BX of ABX system, *J_BA_* = 11.4 Hz, *J_BX_* = 6.4 Hz, 1H, H-6b). **^13^C NMR** (101 MHz, MeOD) δ 170.6 (CO), 164.1 (C-4′), 135.7 (C-2″, C-6″), 130.4 (C-2′, C-6′), 130.0 (C-3″, C-5″), 128.6 (C-4″), 114.7 (C-3′, C-5′), 89.9 (C-1), 75.3 (C-5), 70.2 (C-3), 70.0 (C-4), 62.3 (C-6), 55.9 (OCH_3_), 53.6 (C-2). HRMS (ESI) *m*/*z*: calcd for C_20_H_23_NO_6_Se [M+H]^+^ 454.0765; found 454.0788.

Phenyl 3,4,6-tri-*O*-acetyl-2-(4-cyanobenzamido)-2-deoxy-1-seleno-α-d-galactopyranoside (**25**)

**Procedure d**: Starting material: **2**. Yield: 46%; LC–MS (high-pH method): Rt = 1.17 min; *m*/*z* = 575.000 [M+H]^+^; yellow syrup; ${\left[\alpha \right]}_{D}^{20}$ = +59.2 (*c* 1.52 CH_2_Cl_2_); **^1^H NMR** (400 MHz, CDCl_3_) δ 7.86 (d, *J* = 8.2 Hz, 2H, H-2′, H-6′), 7.76 (d, *J* = 8.1 Hz, 2H, H-3′, H-5′), 7.53 (dt, *J* = 6.9, 1.6 Hz, 2H, H-2″, H-6″), 7.33–7.20 (m, 3H, H-3″, H-4″, H-5″), 6.79 (d, *J*_*NH*,2_ = 8.0 Hz, 1H, NH), 6.24 (d, *J*_1,2_ = 5.0 Hz, 1H, H-1), 5.55 (dd, *J*_4,3_ = 3.4 Hz, *J*_4,5_ = 1.4 Hz, 1H, H-4), 5.26 (dd, *J*_3,2_ = 11.6 Hz, *J*_3,4_ = 3.4 Hz, 1H, H-3), 4.92 (ddd, *J*_2,3_ = 11.6 Hz, *J*_2,*NH*_ = 8.0 Hz, *J*_2,1_ = 5.0 Hz, 1H, H-2), 4.74 (brt, *J*_5,6*a*_ = *J*_5,6*b*_ = 6.7 Hz, 1H, H-5), 4.20, 4.18 (part AX of ABX system, *J_AB_* = 11.4 Hz, *J_AX_* = 5.9 Hz, 1H, H-6a); 4.12, 4.09 (part BX of ABX system, *J_BA_* = 11.4 Hz, *J_BX_* = 7.0 Hz, 1H, H-6b), 2.20 (s, 3H, CH_3_), 2.01 (s, 3H, CH_3_), 1.98 (s, 3H, CH_3_). ^**13**^**C NMR** (101 MHz, CDCl_3_) δ 171.8, 170.4, 170.2, 165.5 (4 × CO), 137.3 (C-1′), 134.2 (C-2″, C-6″), 132.6 (C-3′, C-5′), 129.4 (C-3″, C-5″), 128.3 (C-4″), 127.8 (C-2′, C-6′), 127.7 (C-1″), 117.9 (C≡N), 115.6 (C-4′), 87.3 (C-1), 69.9 (C-5), 69.6 (C-3), 67.0 (C-4), 61.6 (C-6), 50.3 (C-2), 20.8, 20.7, 20.6 (3 × CH_3_). HRMS (ESI) *m*/*z*: calcd for C_26_H_26_N_2_O_8_Se [M+H]^+^ 575.0929; found 575.0962.

Phenyl 2-(4-cyanobenzamido)-2-deoxy-1-seleno-α-d-galactopyranoside (**26**)

**Procedure f**: Starting material: **25**. Yield: 98%; LC–MS (high-pH method): Rt = 0.80 min; *m*/*z* = 449.000 [M+H]^+^; yellow syrup; ${\left[\alpha \right]}_{D}^{20}$ = +50.9 (*c* 0.67 MeOH); ^1^H NMR (400 MHz, MeOD) δ 7.98 (d, *J* = 8.1 Hz, 2H, H-2′, H-6′), 7.85 (d, *J* = 8.1 Hz, 2H, H-3′, H-5′), 7.59–7.52 (m, 2H, H-2″, H-6″), 7.29–7.18 (m, 3H, H-3″, H-4″, H-5″), 6.21 (d, *J*_1,2_ = 5.1 Hz, 1H, H-1), 4.61 (dd, *J*_2,3_ = 11.2 Hz, *J*_2,1_ = 5.1 Hz, 1H, H-2), 4.28 (brt, *J*_5,6a_ = *J*_5,6b_ = 6.1 Hz, 1H, H-5), 4.03 (d, *J*_4,3_ = 3.2 Hz, 1H, H-4), 3.92 (dd, *J*_3,2_ = 11.2 Hz, *J*_3,4_ = 3.2 Hz, 1H, H-3), 3.80, 3.77 (part AX of ABX system, *J_AB_* = 11.4 Hz, *J_AX_* = 5.5 Hz, 1H, H-6a); 3.74, 3.71 (part BX of ABX system, *J_BA_* = 11.4 Hz, *J_BX_* = 6.5 Hz, 1H, H-6b). ^13^C NMR (101 MHz, MeOD) δ 169.3 (CO), 139.9 (C-1′), 135.6 (C-2″, C-6″), 133.5 (C-3′, C-5′), 130.6 (C-1″), 130.1 (C-3″, C-5″), 129.5 (C-2′, C-6′), 128.7 (C-4″), 119.1 (C≡N), 116.1 (C-4′), 89.3 (C-1), 75.4 (C-5), 70.1 (C-3), 69.9 (C-4), 62.3 (C-6), 53.8 (C-2). HRMS (ESI) *m*/*z*: calcd for C_20_H_20_N_2_O_5_Se [M+H]^+^ 449.0611; found 449.0641.

Phenyl 3,4,6-tri-*O*-acetyl-2-deoxy-2-(2-pyrazinamido)-1-seleno-α-d-galactopyranoside (**27**)

**Procedure d**: Starting material: **2**. Yield: 40%; LC–MS (high-pH method): Rt = 1.09 min; *m*/*z* = 552.000 [M+H]^+^; yellow syrup; **^1^H NMR** (400 MHz, CDCl_3_) δ 9.36 (d, *J*_3′,6′_ = 1.5 Hz, 1H, H-3′), 8.79 (d, *J*_4′,6′_ = 2.5 Hz, 1H, H-4′), 8.60 (dd, *J*_6′,4′_ = 2.5 Hz, *J*_6′,3′_ = 1.5 Hz, 1H, H-6′), 8.02 (d, *J*_*NH*,2_ = 8.9 Hz, 1H, NH), 7.54 (dt, J = 6.8, 1.6 Hz, 2H, H-2″, H-6″), 7.29–7.18 (m, 3H, H-3″, H-4″, H-5″), 6.12 (d, *J*_1,2_ = 5.0 Hz, 1H, H-1), 5.53 (dd, *J*_4,3_ = 3.3 Hz, *J*_4,5_ = 1.4 Hz, 1H, H-4), 5.22 (dd, *J*_3,2_ = 11.6 Hz, *J*_3,4_ = 3.2 Hz, 1H, H-3), 4.98 (ddd, *J*_2,3_ = 11.6 Hz, *J*_2,*NH*_ = 8.9 Hz, *J*_2,1_ = 5.0 Hz, 1H, H-2), 4.70 (brt, *J*_5,6*a*_ = *J*_5,6*b*_ = 6.5 Hz, 1H, H-5), 4.21, 4.19 (part AX of ABX system, *J_AB_* = 11.4 Hz, *J_AX_* = 6.0 Hz, 1H, H-6a); 4.15, 4.12 (part BX of ABX system, *J_BA_* = 11.4 Hz, *J_BX_* = 7.1 Hz, 1H, H-6b), 2.20 (s, 3H, CH_3_), 2.04 (s, 3H, CH_3_), 1.97 (s, 3H, CH_3_). ^**13**^**C NMR** (101 MHz, CDCl_3_) δ 170.7, 170.4, 170.2, 163.2 (4 × CO), 147.7 (C-4′), 144.4 (C-3′), 143.6 (C-1′), 142.9 (C-6′), 134.3 (C-2″, C-6″), 129.3 (C-3″, C-5″), 128.2 (C-4″), 128.0 (C-1″), 87.7 (C-1), 69.9 (C-5), 69.4 (C-3), 67.0 (C-4), 61.7 (C-6), 49.1 (C-2), 20.7 (3 × CH_3_). HRMS (ESI) *m*/*z*: calcd for C_23_H_25_N_3_O_8_Se [M+H]^+^ 552.0881; found 552.0918.

Phenyl 2-deoxy-2-(2-pyrazinamido)-1-seleno-α-d-galactopyranoside (**28**)

**Procedure f**: Starting material: **27**. Yield: 97%; LC–MS (high-pH method): Rt = 0.70 min; *m*/*z* = 426.000 [M+H]^+^; yellow syrup; ${\left[\alpha \right]}_{D}^{20}$ = +83.8 (*c* 0.37 MeOH); ^1^H NMR (400 MHz, MeOD) δ 9.21 (d, *J* = 1.5 Hz, 1H, H-3′), 8.81 (d, *J* = 2.5 Hz, 1H, H-6′), 8.72 (dd, *J* = 2.5, 1.5 Hz, 1H, H-4′), 7.55 (dt, *J* = 6.7, 1.7 Hz, 2H, H-2″, H-6″), 7.29–7.13 (m, 3H, H-3″, H-4″, H-5″), 6.13 (d, *J*_1,2_ = 4.9 Hz, 1H, H-1), 4.65 (dd, *J*_2,3_ = 11.1 Hz, *J*_2,1_ = 4.9 Hz, 1H, H-2), 4.26 (brt, *J*_5,6a_ = *J*_5,6b_ = 6.0 Hz, 1H, H-5), 4.05 (dd, *J*_4,3_ = 3.1 Hz, *J*_4,5_ = 1.3 Hz, 1H, H-4), 3.90 (dd, *J*_3,2_ = 11.1 Hz, *J*_3,4_ = 3.1 Hz, 1H, H-3), 3.81, 3.79 (part AX of ABX system, *J_AB_* = 11.5 Hz, *J_AX_* = 5.5 Hz, 1H, H-6a); 3.77, 3.74 (part BX of ABX system, *J_BA_* = 11.5 Hz, *J_BX_* = 6.7 Hz, 1H, H-6b). **^13^C NMR** (101 MHz, MeOD) δ 165.6 (CO), 148.8 (C-6′), 145.8 (C-1′), 144.8 (C-4′), 144.7 (C-3′), 135.6 (C-2″, C-6″), 130.5 (C-1″), 130.1 (C-3″, C-5″), 128.8 (C-4″), 89.9 (C-1), 75.9 (C-5), 70.8 (C-3), 70.0 (C-4), 62.4 (C-6), 53.1 (C-2). HRMS (ESI) *m*/*z*: calcd for C_17_H_19_N_3_O_5_Se [M+H]^+^ 426.0564; found 426.0591.

Phenyl 3,4,6-tri-*O*-acetyl-2-deoxy-2-(5-fluoropicolinamido)-1-seleno-α-d-galactopyranoside (**29**)

**Procedure d**: Starting material: **2**. Yield: 35%; LC–MS (high-pH method): Rt = 1.21 min; *m*/*z* = 569.200 [M+H]^+^; yellow syrup; **^1^H NMR** (400 MHz, CDCl_3_) δ 8.46 (d, *J*_3′,*F*_ = 2.8 Hz, 1H, H-3′), 8.19 (dd, *J*_6′,5′_ = 8.7 Hz, *J*_6′,*F*_ = 4.5 Hz, 1H, H-6′), 8.08 (d, *J*_*NH*,2_ = 9.0 Hz, 1H, NH), 7.58–7.51 (m, 3H, H-5′, H-3″, H-5″), 7.31–7.16 (m, 3H, H-2″, H-4″, H-6″), 6.11 (d, *J*_1,2_ = 5.0 Hz, 1H, H-1), 5.52 (dd, *J*_4,3_ = 3.3 Hz, *J*_4,5_ = 1.4 Hz, 1H, H-4), 5.21 (dd, *J*_3,2_ = 11.6 Hz, *J*_3,4_ = 3.3 Hz, 1H, H-3), 4.95 (ddd, *J*_2,3_ = 11.6 Hz, *J_2,_*_NH_ = 9.0 Hz, *J*_2,1_ = 5.0 Hz, 1H, H-2), 4.70 (brt, *J*_5,6*a*_ = *J*_5,6*b*_ = 6.4 Hz, 1H, H-5), 4.21, 4.18 (part AX of ABX system, *J_AB_* = 11.4 Hz, *J_AX_* = 5.9 Hz, 1H, H-6a); 4.14, 4.11 (part BX of ABX system, *J_BA_* = 11.4 Hz, *J_BX_* = 7.1 Hz, 1H, H-6b), 2.19 (s, 3H, CH_3_), 2.03 (s, 3H, CH_3_), 1.96 (s, 3H, CH_3_). **^13^C NMR** (101 MHz, CDCl_3_) δ 170.6, 170.4, 170.3, 163.5 (4 × CO), 161.4 (d, *J* = 261.5 Hz, C-5′ [CF]), 145.3 (d, *J* = 3.9 Hz, C-2′), 137.1 (d, *J* = 25.4 Hz, C-6′), 134.3 (C-2″, C-6″), 129.2 (C-3″, C-5″), 128.2 (C-1″), 128.1 (C-4″), 124.2 (d, *J* = 5.6 Hz, C-3′), 123.9 (d, *J* = 18.6 Hz, C-4′), 87.9 (C-1), 69.8 (C-5), 69.5 (C-3), 67.1 (C-4), 61.7 (C-6), 49.0 (C-2), 20.7 (3 × CH_3_).

Phenyl 2-deoxy-2-(5-fluoropicolinamido)-1-seleno-α-d-galactopyranoside (**30**)

**Procedure f**: Starting material: **29**. Yield: 78%; LC–MS (high-pH method): Rt = 0.84 min; *m*/*z* = 443.000 [M+H]^+^; yellow syrup; ${\left[\alpha \right]}_{D}^{20}$ = +90.2 (*c* 0.45 MeOH); **^1^H NMR** (400 MHz, MeOD) δ 8.57 (d, *J* = 2.9 Hz, 1H, H-6′), 8.14 (dd, *J* = 8.7, 4.5 Hz, 1H, H-3′), 7.75 (td, *J* = 8.5, 2.8 Hz, 1H, H-4′), 7.55 (dt, *J* = 6.7, 1.6 Hz, 2H, H-2″, H-6″), 7.28–7.13 (m, 3H, H-3″, H-4″, H-5″), 6.12 (d, *J*_1,2_ = 4.9 Hz, 1H, H-1), 4.61 (dd, *J*_2,3_ = 11.1 Hz, *J*_2,1_ = 4.9 Hz, 1H, H-2), 4.26 (brt, *J*_5,6a_ = *J*_5,6b_ = 6.0 Hz, 1H, H-5), 4.04 (dd, *J*_4,3_ = 3.1 Hz, *J*_4,5_ = 1.3 Hz, 1H, H-4), 3.86 (dd, *J*_3,2_ = 11.1 Hz, *J*_3,4_ = 3.1 Hz, 1H, H-3), 3.81, 3.78 (part AX of ABX system, *J_AB_* = 11.4 Hz, *J_AX_* = 5.6 Hz, 1H, H-6a); 3.76, 3.74 (part BX of ABX system, *J_BA_* = 11.5 Hz, *J_BX_* = 6.5 Hz, 1H, H-6b). **^13^C NMR** (101 MHz, MeOD) δ 165.9 (CO), 162.9 (d, *J* = 259.7 Hz, C-5′), 147.1 (d, *J* = 3.9 Hz, C-2′), 138.2 (d, *J* = 25.7 Hz, C-6′), 135.6 (C-2″, C-6″), 130.6 (C-1″), 130.1 (C-3″, C-5″), 128.7 (C-4″), 125.3 (d, *J* = 8.5 Hz, C-4′), 125.2 (d, *J* = 4.7 Hz, C-3′), 90.2 (C-1), 75.9 (C-5), 71.0 (C-3), 70.0 (C-4), 62.4 (C-6), 53.1 (C-2). LC–MS (high-pH method): Rt = 0.84 min; *m*/*z* = 443.000 [M+H]^+^. HRMS (ESI) *m*/*z*: calcd for C_18_H_19_FN_2_O_5_Se [M+H]^+^ 443.0517; found 443.0544.

Phenyl 3,4,6-tri-*O*-acetyl-2-deoxy-2-(5-pyrimidinamido)-1-seleno-α-d-galactopyranoside (**31**)

**Procedure d**: Starting material: **2**. Yield: 38%; LC–MS (high-pH method): Rt = 1.00 min; *m*/*z* = 552.000 [M+H]^+^; yellow syrup; **^1^H NMR** (400 MHz, CDCl_3_) δ 9.34 (s, 1H, H-4′), 9.10 (s, 2H, H-2′, H-6′), 7.63–7.46 (m, 2H, H-2″, H-6″), 7.35–7.18 (m, 3H, H-3″, H-4″, H-5″), 7.07 (d, *J*_*NH*,2_ = 7.9 Hz, 1H, NH), 6.26 (d, *J*_1,2_ = 5.0 Hz, 1H, H-1), 5.57 (dd, *J*_4,3_ = 3.3 Hz, *J*_4,5_ = 1.3 Hz, 1H, H-4), 5.32 (dd, *J*_3,2_ = 11.6 Hz, *J*_3,4_ = 3.3 Hz, 1H, H-3), 4.95 (ddd, *J*_2,3_ = 11.6 Hz, *J*_2,*NH*_ = 7.9 Hz, *J*_2,1_ = 5.0 Hz, 1H, H-2), 4.77 (brt, *J*_5,6*a*_ = *J*_5,6*b*_ = 6.5 Hz, 1H, H-5), 4.20, 4.17 (part AX of ABX system, *J_AB_* = 11.4 Hz, *J_AX_* = 6.1 Hz, 1H, H-6a); 4.12, 4.09 (part BX of ABX system, *J_BA_* = 11.4 Hz, *J_BX_* = 6.9 Hz, 1H, H-6b), 2.20 (s, 3H, CH_3_), 2.04 (s, 3H, CH_3_), 1.93 (s, 3H, CH_3_). **^13^C NMR** (101 MHz, CDCl_3_) δ 171.9, 170.4, 170.2, 163.7 (4 × CO), 160.9 (C-4′), 155.8 (C-2′, C-6′), 134.3 (C-2″, C-6″), 129.4 (C-3″, C-5″), 128.3 (C-4″), 127.5 (C-1″), 127.1 (C-5′), 87.1 (C-1), 69.9 (C-5), 69.5 (C-3), 66.9 (C-4), 61.5 (C-6), 50.1 (C-2), 20.9, 20.7, 20.5 (3 × CH_3_).

Phenyl 2-deoxy-2-(5-pyrimidinamido)-1-seleno-α-d-galactopyranoside (**32**)

**Procedure f**: Starting material: **31**. Yield: 94%; LC–MS (high-pH method): Rt = 0.63 min; *m*/*z* = 425.800 [M+H]^+^; yellow syrup; ${\left[\alpha \right]}_{D}^{20}$ = +125.8 (*c* 0.23 MeOH); **^1^H NMR** (400 MHz, MeOD) δ 9.29 (s, 1H, H-2′), 9.15 (s, 2H, H-4′, H-6′), 7.57 (dd, *J* = 7.3, 2.2 Hz, 2H, H-2″, H-6″), 7.28–7.17 (m, 3H, H-3″, H-4″, H-5″), 6.21 (d, *J*_1,2_ = 5.1 Hz, 1H, H-1), 4.62 (dd, *J*_2,3_ = 11.3 Hz, *J*_2,1_ = 5.1 Hz, 1H, H-2), 4.27 (brt, *J*_5,6a_ = *J*_5,6b_ = 6.1 Hz, 1H, H-5), 4.04 (d, *J*_4,3_ = 3.1 Hz, 1H, H-4), 3.93 (dd, *J*_3,2_ = 11.3 Hz, *J*_3,4_ = 3.1 Hz, 1H, H-3), 3.80, 3.77 (part AX of ABX system, *J_AB_* = 11.4 Hz, *J_AX_* = 5.7 Hz, 1H, H-6a); 3.74, 3.71 (part BX of ABX system, *J_BA_* = 11.4 Hz, *J_BX_* = 6.5 Hz, 1H, H-6b). **^13^C NMR** (101 MHz, MeOD) δ 166.5 (CO), 161.1 (C-2′), 157.3 (C-4′, C-6′), 135.6 (C-2″, C-6″), 130.1 (C-3″, C-5″), 129.8 (C-1″), 128.7 (C-4″), 89.1 (C-1), 75.5 (C-5), 70.1 (C-3), 69.9 (C-4), 62.3 (C-6), 53.8 (C-2). HRMS (ESI) *m*/*z*: calcd for C_17_H_19_N_3_O_5_Se [M+H]^+^ 426.0564; found 426.0595.

Phenyl 3,4,6-tri-*O*-acetyl-2-deoxy-2-(3-phenylureido)-1-seleno-α-d-galactopyranoside (**33**)

**Procedure d**: Starting material: **2**. Yield: 67%; LC–MS (high-pH method): Rt = 1.17 min; *m*/*z* = 565.000 [M+H]^+^; yellow syrup; ${\left[\alpha \right]}_{D}^{20}$ = +69.7 (*c* 2.21 CH_2_Cl_2_); **^1^H NMR** (400 MHz, CDCl_3_) δ 7.55 (brd, *J* = 8.0 Hz, 2H, H-2″, H-6″), 7.35–7.21 (m, 7H, H-2′, H-3′, H-5′, H-6′, H-3″, H-4″, H-5″), 7.19 (s, 1H, CONHPh), 7.07 (tt, *J* = 6.9, 1.6 Hz, 1H, H-4′), 6.18 (d, *J*_1,2_ = 5.0 Hz, 1H, H-1), 5.47 (d, *J*_*NH*,2_ = 8.5 Hz, 1H, NH), 5.46 (d, *J*_4,3_ = 3.3 Hz, 1H, H-4), 5.14 (dd, *J*_3,2_ = 11.6 Hz, *J*_3,4_ = 3.3 Hz, 1H, H-3), 4.73 (ddd, *J*_2,3_ = 11.6 Hz, *J*_2,*NH*_ = 8.5 Hz, *J*_2,1_ = 5.0 Hz, 1H, H-2), 4.67 (brt, *J*_5,6*a*_ = *J*_5,6*b*_ = 6.5 Hz, 1H, H-5), 4.14, 4.12 (part AX of ABX system, *J_AB_* = 11.4 Hz, *J_AX_* = 6.0 Hz, 1H, H-6a); 4.05, 4.03 (part BX of ABX system, *J_BA_* = 11.3 Hz, *J_BX_* = 7.0 Hz, 1H, H-6b), 2.10 (s, 3H, CH_3_), 2.01 (s, 3H, CH_3_), 1.94 (s, 3H, CH_3_). ^**13**^**C NMR** (101 MHz, CDCl_3_) δ 169.6, 168.5, 168.5, 153.0 (4 × CO), 136.4 (C-1′), 132.4 (C-2″, C-6″), 127.4 (C-3′, C-5′, C-3″, C-5″), 126.2 (C-4″), 126.0 (C-1″), 121.9 (C-4′), 118.5 (C-2′, C-6′), 87.0 (C-1), 67.8 (C-5), 67.7 (C-3), 65.2 (C-4), 59.8 (C-6), 47.9 (C-2), 19.0, 18.7, 18.7 (3 × CH_3_). HRMS (ESI) *m*/*z*: calcd for C_25_H_28_N_2_O_8_Se [M+H]^+^ 565.1086; found 565.1119.

Phenyl 2-deoxy-2-(3-phenylureido)-1-seleno-α-d-galactopyranoside (compound **34**)

**Procedure f**: Starting material: **33**. Yield: 90%; LC–MS (high-pH method): Rt = 0.83 min; *m*/*z* = 439.000 [M+H]^+^; yellow syrup; ${\left[\alpha \right]}_{D}^{20}$ = +144.5 (*c* 0.53 MeOH); **^1^H NMR** (400 MHz, MeOD) δ 7.67–7.59 (m, 2H, H-2′, H-6′), 7.38–7.31 (m, 2H, H-3′, H-5′), 7.30–7.20 (m, 5H, H-2″, H-3″, H-4″, H-5″), 6.98 (t, *J* = 7.3 Hz, 1H, H-4′), 6.11 (d, *J*_1,2_ = 5.0 Hz, 1H, H-1), 4.33 (dd, *J*_2,3_ = 11.1 Hz, *J*_2,1_ = 5.0 Hz, 1H, H-2), 4.23 (brt, *J*_5,6a_ = *J*_5,6b_ = 6.2 Hz, 1H, H-5), 3.99 (d, *J*_4,3_ = 2.7 Hz, 1H, H-4), 3.77, 3.74 (part AX of ABX system, *J_AB_* = 11.4 Hz, *J_AX_* = 5.7 Hz, 1H, H-6a); 3.71, 3.68 (part BX of ABX system, *J_BA_* = 11.4 Hz, *J_BX_* = 6.5 Hz, 1H, H-6b), 3.63 (dd, *J*_3,2_ = 11.1 Hz, *J*_3,4_ = 3.1 Hz, 1H, H-3). **^13^C NMR** (101 MHz, MeOD) δ 158.1 (CO), 135.8 (C-2″, C-6″), 130.6 (C-1″), 130.1 (C-3″, C-5″), 129.8 (C-2′, C-6′), 128.7 (C-4″), 125.1 (C-1′), 123.6 (C-4′), 120.2 (C-3′, C-5′), 91.3 (C-1), 75.4 (C-5), 71.5 (C-3), 70.0 (C-4), 62.3 (C-6), 53.0 (C-2). HRMS (ESI) *m*/*z*: calcd for C_19_H_22_N_2_O_5_Se [M+H]^+^ 439.0768; found 439.0791.

Phenyl 3,4,6-tri-*O*-acetyl-2-deoxy-2-[3-(pyridin-3-yl)ureido]-1-seleno-α-d-galactopyranoside (**35**)

**Procedure d**: Starting material: **2**. Yield: 36%; LC–MS (high-pH method): Rt = 1.02 min; *m*/*z* = 566.000 [M+H]^+^: yellow syrup; **^1^H NMR** (400 MHz, CDCl_3_) δ 8.40 (d, *J* = 2.6 Hz, 1H, H-2′), 8.27 (dd, *J* = 4.7, 1.5 Hz, 1H, H-6′), 8.03 (ddd, *J* = 8.3, 2.7, 1.5 Hz, 1H, H-4′), 7.55 (dd, *J* = 7.8, 1.7 Hz, 2H, H-2″, H-6″), 7.49 (s, 1H, CONH), 7.30–7.22 (m, 4H, H-5′, H-3″, H-4″, H-5″), 6.15 (d, *J*_1,2_ = 5.0 Hz, 1H, H-1), 5.47–5.43 (m, 2H, H-4, NH), 5.10 (dd, *J*_3,2_ = 11.5 Hz, *J*_3,4_ = 3.3 Hz, 1H, H-3), 4.70 (ddd, *J*_2,3_ = 11.5 Hz, *J*_2,*NH*_ = 8.6 Hz, *J*_2,1_ = 5.0 Hz, 1H, H-2), 4.65 (brt, *J*_5,6*a*_ = *J*_5,6*b*_ = 6.5 Hz, 1H, H-5), 4.17, 4.14 (part AX of ABX system, *J_AB_* = 11.4 Hz, *J_AX_* = 6.0 Hz, 1H, H-6a); 4.08, 4.05 (part BX of ABX system, *J_BA_* = 11.4 Hz, *J_BX_* = 7.0 Hz, 1H, H-6b), 2.17 (s, 3H, CH_3_), 2.04 (s, 3H, CH_3_), 1.97 (s, 3H, CH_3_). ^**13**^**C NMR** (101 MHz, CDCl_3_) δ 171.6, 170.4, 170.3, 154.4 (4 × CO), 144.0 (C-6′), 140.5 (C-2′), 135.9 (C-1′), 134.2 (C-2″, C-6″), 129.4 (C-3″, C-5″), 128.2 (C-4″), 127.9 (C-1″), 126.6 (C-4′), 123.9 (C-5′), 88.7 (C-1), 69.8 (C-5), 69.7 (C-3), 67.1 (C-4), 61.7 (C-6), 49.9 (C-2), 20.9, 20.7, 20.6 (3 × CH_3_).

Phenyl 2-deoxy-2-[3-(pyridin-3-yl)ureido]-1-seleno-α-d-galactopyranoside (**36**)

**Procedure f**: Starting material: **35**. Yield: 87%; LC–MS (high-pH method): Rt = 0.66 min; *m*/*z* = 440.000 [M+H]^+^; yellow syrup; ${\left[\alpha \right]}_{D}^{20}$ = +147.6 (*c* 0.52 MeOH); **^1^H NMR** (400 MHz, MeOD) δ 8.52 (d, *J* = 2.4 Hz, 1H, H-2′), 8.14 (dd, *J* = 4.8, 1.5 Hz, 1H, H-6′), 7.92 (ddd, *J* = 8.4, 2.6, 1.4 Hz, 1H, H-4′), 7.66–7.58 (m, 2H, H-2″, H-6″), 7.33 (ddd, *J* = 8.3, 4.8, 0.8 Hz, 1H, H-5′), 7.27–7.19 (m, 3H, H-3″, H-4″, H-5″), 6.11 (d, *J*_1,2_ = 4.9 Hz, 1H, H-1), 4.34 (dd, *J*_2,3_ = 11.1 Hz, *J*_2,1_ = 4.9 Hz, 1H, H-2), 4.23 (brt, *J*_5,6a_ = *J*_5,6b_ = 6.2 Hz, 1H, H-5), 4.00 (dd, *J*_4,3_ = 3.1 Hz, *J*_4,5_ = 1.3 Hz, 1H, H-4), 3.78, 3.75 (part AX of ABX system, *J_AB_* = 11.4 Hz, *J_AX_* = 5.6 Hz, 1H, H-6a); 3.72, 3.69 (part BX of ABX system, *J_BA_* = 11.4 Hz, *J_BX_* = 6.6 Hz, 1H, H-6b), 3.66 (dd, *J*_3,2_ = 11.1 Hz, *J*_3,4_ = 3.1 Hz, 1H, H-3). **^13^C NMR** (101 MHz, MeOD) δ 157.6 (CO), 143.4 (C-6′), 140.7 (C-2′), 138.5 (C-3′), 135.7 (C-2″, C-6″), 130.6 (C-1″), 130.1 (C-3″, C-5″), 128.7 (C-4″), 127.7 (C-4′), 125.3 (C-5′), 91.1 (C-1), 75.6 (C-5), 71.4 (C-3), 70.0 (C-4), 62.3 (C-6), 53.1 (C-2). HRMS (ESI) *m*/*z*: calcd for C_18_H_21_N_3_O_5_Se [M+H]^+^ 440.0720; found 440.0746.

Phenyl 3,4,6-tri-*O*-acetyl-2-deoxy-2-[3-(4-fluorophenyl)ureido]-1-seleno-α-d-galactopyranoside (**37**)

**Procedure d**: Starting material: **2**. Yield: 41%; LC–MS (high-pH method): Rt = 1.21 min; *m*/*z* = 583.000 [M+H]^+^; yellow syrup; ${\left[\alpha \right]}_{D}^{20}$ = +69.0 (*c* 0.71 CH_2_Cl_2_); **^1^H NMR** (400 MHz, CDCl_3_) δ 7.54 (brd, *J* = 6.9 Hz, 2H, H-2″, H-6″), 7.35–7.21 (m, 5H, H-2′, H-6′, H-3″, H-4″, H-5″), 7.01 (t, *J* = 8.5 Hz, 2H, H-3′, H-5′), 6.63 (s, 1H, CONH), 6.13 (d, *J*_1,2_ = 5.0 Hz, 1H, H-1), 5.45 (dd, *J*_4,3_ = 3.4 Hz, *J*_4,5_ = 1.4 Hz, 1H, H-4), 5.12–5.02 (m, 2H, NH, H-3), 4.68 (ddd, *J*_2,3_ = 11.7 Hz, *J*_2,*NH*_ = 8.6 Hz, *J*_2,1_ = 5.0 Hz, 1H, H-2), 4.63 (brt, *J*_5,6*a*_ = *J*_5,6*b*_ = 6.4 Hz, 1H, H-5), 4.16, 4.13 (part AX of ABX system, *J_AB_* = 11.4 Hz, *J_AX_* = 6.0 Hz, 1H, H-6a); 4.07, 4.04 (part BX of ABX system, *J_BA_* = 11.4 Hz, *J_BX_* = 7.0 Hz, 1H, H-6b), 2.15 (s, 3H, CH_3_), 2.03 (s, 3H, CH_3_), 1.98 (s, 3H, CH_3_). **^13^C NMR** (101 MHz, CDCl_3_) δ 171.4, 170.4, 170.3 (3 × CO), 159.5 (d, *J* = 243.6 Hz, C-4′), 154.7 (NHCONH), 134.1 (C-2″, C-6″), 133.9 (d, *J* = 2.7 Hz, C-1′), 129.4 (C-3″, C-5″), 128.1 (C-4″), 128.0 (C-1″), 122.9 (d, *J* = 7.5 Hz, C-2′, C-6′), 116.0 (d, *J* = 22.5 Hz, C-3′, C-5′), 88.9 (C-1), 69.8 (C-5), 69.6 (C-3), 67.1 (C-4), 61.7 (C-6), 50.1 (C-2), 20.9, 20.7, 20.6 (3 × CH_3_). HRMS (ESI) *m*/*z*: calcd for C_25_H_27_FN_2_O_8_Se [M+H]^+^ 583.0991; found 583.1021.

Phenyl 2-deoxy-2-[3-(4-fluorophenyl)ureido]-1-seleno-α-d-galactopyranoside (**38**)

**Procedure f**: Starting material: **37**. Yield: 92%; LC–MS (high-pH method): Rt = 0.88 min; *m*/*z* = 456.800 [M+H]^+^; yellow syrup; ${\left[\alpha \right]}_{D}^{20}$ = +75.2 (*c* 0.53 MeOH); **^1^H NMR** (400 MHz, MeOD) δ 7.65–7.58 (m, 2H, H-2″, H-6″), 7.48–7.37 (m, 1H, H-2′), 7.35–7.29 (m, 1H, H-6′), 7.28–7.18 (m, 3H, H-3″, H-4″, H-5″), 7.01 (ddd, *J* = 14.4, 9.8, 7.7 Hz, 2H, H-3′, H-5′), 6.10 (d, *J*_1,2_ = 4.9 Hz, 1H, H-1), 4.32 (dd, *J*_2,3_ = 11.1 Hz, *J*_2,1_ = 4.9 Hz, 1H, H-2), 4.23 (brt, *J*_5,6a_ = *J*_5,6b_ = 5.8 Hz, 1H, H-5), 3.99 (d, J_4,3_ = 2.7 Hz, 1H, H-4), 3.77, 3.74 (part AX of ABX system, *J_AB_* = 11.4 Hz, *J_AX_* = 5.7 Hz, 1H, H-6a); 3.71, 3.68 (part BX of ABX system, *J_BA_* = 11.4 Hz, *J_BX_* = 6.6 Hz, 1H, H-6b), 3.63 (dd, *J*_3,2_ = 11.1 Hz, *J*_3,4_ = 3.1 Hz, 1H, H-3). ^**13**^**C NMR** (101 MHz, MeOD) δ 160.9 (d, *J* = 240.3 Hz, C-4′), 155.4 (CO), 135.7 (C-2″, C-6″), 132.1 (d, *J* = 2.4 Hz, C-1′), 130.1 (C-3″, C-5″), 128.7 (C-4″), 122.4 (d, *J* = 7.7 Hz, C-2′), 122.0 (d, *J* = 7.7 Hz, C-6′), 116.3 (d, *J* = 22.6 Hz, C-3′, C-5′), 91.2 (C-1), 75.5 (C-5), 71.5 (C-3), 70.0 (C-4), 62.3 (C-6), 53.3 (C-2). HRMS (ESI) *m*/*z*: calcd for C_19_H_21_FN_2_O_5_Se [M+H]^+^ 457.0674; found 457.0691.

Phenyl 3,4,6-tri-*O*-acetyl-2-deoxy-2-(2,2,2-trifluoroethylureido)-1-seleno-α-d-galactopyranoside (**39**)

**Procedure d**: Starting material: **2**. Yield: 51%; LC–MS (high-pH method): Rt = 1.11 min; *m*/*z* = 571.000 [M+H]^+^; yellow syrup; **^1^H NMR** (400 MHz, CDCl_3_) δ 7.57 (dt, *J* = 6.5, 1.7 Hz, 2H, H-2″, H-6″), 7.36–7.20 (m, 3H, H-3″, H-4″, H-5″), 6.07 (d, *J*_1,2_ = 5.0 Hz, 1H, H-1), 5.45 (dd, *J*_4,3_ = 3.3 Hz, *J*_4,5_ = 1.3 Hz, 1H, H-4), 5.32 (t, *J*_*CONH*,*CH*2_ = 6.2 Hz, 1H, CONH), 5.22 (d, *J*_*NH*,2_ = 8.7 Hz, 1H, NH), 5.11 (dd, *J*_3,2_ = 11.5 Hz, *J*_3,4_ = 3.3 Hz, 1H, H-3), 4.74–4.58 (m, 2H, H-2, H-5), 4.14, 4.11 (part AX of ABX system, *J_AB_* = 11.3 Hz, *J_AX_* = 6.2 Hz, 1H, H-6a), 4.07–3.92 (m, 2H, H-6b, H-7a), 3.69 (ddd, *J* = 15.0, 9.0, 5.8 Hz, 1H, H-7b), 2.16 (s, 3H, CH_3_), 2.03 (s, 3H, CH_3_), 1.91 (s, 3H, CH_3_). **^13^C NMR** (101 MHz, CDCl_3_) δ 171.9, 170.6, 170.5, 156.4 (4 × CO), 134.5 (C-2″, C-6″), 129.5 (C-3″, C-5″), 128.3 (C-4″), 128.0 (C-1″), 124.5 (q, *J* = 278.7 Hz, CF_3_), 89.3 (C-1), 70.1 (C-5), 69.9 (C-3), 67.2 (C-4), 61.7 (C-6), 50.1 (C-2), 41.7 (q, *J* = 34.3 Hz, C-7), 20.9, 20.8, 20.6 (3 ×CH_3_).

Phenyl 2-deoxy-2-(2,2,2-trifluoroethylureido)-1-seleno-α-d-galactopyranoside (**40**)

**Procedure f**: Starting material: **39**. Yield: 97%; LC–MS (high-pH method): Rt = 0.75 min; *m*/*z* = 445.000 [M+H]^+^; yellow syrup; ${\left[\alpha \right]}_{D}^{20}$ = +184.1 (*c* 0.72 MeOH); ^**1**^**H NMR** (400 MHz, MeOD) δ 7.64–7.53 (m, 2H, H-2″, H-6″), 7.31–7.20 (m, 3H, H-3″, H-4″, H-5″), 6.04 (d, *J*_1,2_ = 4.9 Hz, 1H, H-1), 4.29–4.16 (m, 2H, H-2, H-5), 3.96 (dd, *J*_4,3_ = 3.2 Hz, *J*_4,5_ = 1.3 Hz, 1H, H-4), 3.82 (q, *J* = 9.4 Hz, 2H, NHCH_2_), 3.76, 3.73 (part AX of ABX system, *J_AB_* = 11.4 Hz, *J_AX_* = 5.7 Hz, 1H, H-6a), 3.70, 3.67 (part BX of ABX system, *J_BA_* = 11.4 Hz, *J_BX_* = 6.5 Hz, 1H, H-6b), 3.58 (dd, *J*_3,2_ = 11.1 Hz, *J*_3,4_ = 3.2 Hz, 1H, H-3). **^13^C NMR** (101 MHz, MeOD) δ 160.0 (CO), 135.8 (C-2″, C-6″), 130.7 (C-1″), 130.0 (C-3″, C-5″), 128.6 (C-4″), 126.2 (q, *J* = 278.4 Hz, CF_3_), 91.4 (C-1), 75.4 (C-5), 71.3 (C-3), 70.0 (C-4), 62.3 (C-6), 53.3 (C-2), 42.1 (q, *J* = 34.5 Hz, NHCH_2_). HRMS (ESI) *m*/*z*: calcd for C_15_H_19_F_3_N_2_O_5_Se [M+H]^+^ 445.0485; found 445.0503.

Phenyl 3,4,6-tri-*O*-acetyl-2-azido-2-deoxy-1-thio-α-d-galactopyranoside (**41**)

Compound **1** (1.609 mmol) and Ph_2_Se_2_ (2 eq.) were dissolved in dry DCM (10 mL). Then, PhI(OAc)_2_ (1.5 eq.) and TMSN_3_ (3 eq.) were added. The mixture was stirred at room temperature, under a nitrogen atmosphere and followed by LCMS. After 48 h, the mixture was evaporated, and the crude submitted to preparative HPLC. Yield: 9%; LC–MS (high-pH method): Rt = 1.23 min; *m*/*z* = 441.200 [M+OH]^−^; transparent syrup; ${\left[\alpha \right]}_{D}^{20}$ = +189.8 (*c* 0.98 CH_2_Cl_2_); **^1^H NMR** (400 MHz, CDCl_3_) δ 7.56–7.46 (m, 2H, H-2″, H-6″), 7.37–7.27 (m, 3H, H-3″, H-4″, H-5″), 5.70 (d, *J*_1,2_ = 5.5 Hz, 1H, H-1), 5.49 (dd, *J*_4,3_ = 3.4 Hz, *J*_4,5_ = 1.4 Hz, 1H, H-4), 5.18 (dd, *J*_3,2_ = 11.1 Hz, *J*_3,4_ = 3.4 Hz, 1H, H-3), 4.76 (td, *J*_5,6*a*_ = *J*_5,6*b*_ = 6.4 Hz, *J*_5,4_ = 1.4 Hz, 1H, H-5), 4.32 (dd, *J*_2,3_ = 11.1 Hz, *J*_2,1_ = 5.5 Hz, 1H, H-2), 4.17–4.05 (m, 2H, H-6a, H-6b), 2.16 (s, 3H, CH_3_), 2.07 (s, 3H, CH_3_), 1.99 (s, 3H, CH_3_). **^13^C NMR** (101 MHz, CDCl_3_) δ 170.3, 169.9, 169.5 (3 × CO), 132.5 (C-2″, C-6″), 132.4 (C-1″), 129.1 (C-3″, C-5″), 128.0 (C-4″), 86.9 (C-1), 70.1 (C-3), 67.5 (C-5), 67.4 (C-4), 61.7 (C-6), 58.1 (C-2), 20.6 (3 × CH_3_). HRMS (ESI) *m*/*z*: calcd for C_18_H_25_N_3_O_7_S [M+NH_4_]^+^ 441.1438; found 441.1466.

Phenyl 3,4,6-tri-*O*-acetyl-2-deoxy-2-[6-(trifluoromethyl)nicotinamido]-1-thio-α-d-galactopyranoside (**42**)

**Procedure d**: Starting material: **41**. Yield: 34%; LC–MS (high-pH method): Rt = 1.24 min; *m*/*z* = 569.000 [M-H]^−^; transparent syrup; ${\left[\alpha \right]}_{D}^{20}$ = +47.8 (*c* 0.69 CH_2_Cl_2_); **^1^H NMR** (400 MHz, CDCl_3_) δ 9.10 (d, *J*_2′,6′_ = 2.2 Hz, 1H, H-2′), 8.27 (dd, *J*_4′,5′_ = 8.2 Hz, *J*_4′,2′_ = 2.2 Hz, 1H, H-4′), 7.80 (d, *J*_5′,4′_ = 8.2 Hz, 1H, H-5′), 7.50–7.40 (m, 2H, H-2″, H-6″), 7.33–7.26 (m, 3H, H-3″, H-4″, H-5″), 6.90 (d, *J*_*NH*,2′_ = 8.3 Hz, 1H, NH), 5.94 (d, *J*_1,2_ = 5.3 Hz, 1H, H-1), 5.55 (dd, *J*_4,3_ = 3.4 Hz, *J*_4,5_ = 1.4 Hz, 1H, H-4), 5.34 (dd, *J*_3,2_ = 11.7 Hz, *J*_3,4_ = 3.4 Hz, 1H, H-3), 5.05 (ddd, *J*_2,3_ = 11.6 Hz, *J*_2,*NH*_ = 8.3 Hz, *J*_2,1_ = 5.3 Hz, 1H, H-2), 4.84 (brt, *J*_5,6*a*_ = *J*_5,6*b*_ = 6.5 Hz, 1H, H-5), 4.19, 4.17 (part AX of ABX system, J_AB_ = 11.4 Hz, J_AX_ = 5.9 Hz, 1H, H-6a); 4.14, 4.12 (part BX of ABX system, J_BA_ = 11.4 Hz, J_BX_ = 7.1 Hz, 1H, H-6b), 2.21 (s, 3H, CH_3_), 2.03 (s, 3H, CH_3_), 1.97 (s, 3H, CH_3_). **^13^C NMR** (101 MHz, CDCl_3_) δ 171.8, 170.4, 170.2, 164.2 (4 × CO), 150.8 (q, *J* = 35.3 Hz, C-6′), 148.6 (C-2′), 136.7 (C-4′), 132.3 (C-1″), 132.1 (C-2″, C-6″), 131.8 (C-3′), 129.3 (C-3″, C-5″), 128.2 (C-4″), 121.1 (q, *J* = 274.4 Hz, CF_3_), 120.5 (q, *J* = 2.8 Hz, C-5′), 88.2 (C-1), 68.8 (C-3), 68.1 (C-5), 67.3 (C-4), 61.8 (C-6), 49.8 (C-2), 20.8, 20.7, 20.6 (3 × CH_3_). HRMS (ESI) *m*/*z*: calcd for C_25_H_25_F_3_N_2_O_8_S [M+H]^+^ 571.1356; found 571.1404.

Phenyl 2-deoxy-2-[6-(trifluoromethyl)nicotinamido]-1-thio-α-d-galactopyranoside (**43**)

**Procedure f**: Starting material: **42**. Yield: 97%; LC–MS (high-pH method): Rt = 0.93 min; *m*/*z* = 445.000 [M+H]^+^; transparent syrup; ${\left[\alpha \right]}_{D}^{20}$ = +73.7 (*c* 0.19 MeOH); **^1^H NMR** (400 MHz, MeOD) δ 9.03 (d, *J*_2′,6′_ = 2.1 Hz, 1H, H-2′), 8.34 (dd, *J*_4′,5′_ = 8.0 Hz, *J*_4′,2′_ = 2.2 Hz, 1H, H-4′), 7.84 (dd, *J*_5′,4′_ = 8.2 Hz, *J*_5′,2′_ = 0.9 Hz, 1H, H-5′), 7.41–7.35 (m, 2H, H-2″, H-6″), 7.21–7.10 (m, 3H, H-3″, H-4″, H-5″), 5.79 (d, *J*_1,2_ = 5.4 Hz, 1H, H-1), 4.63 (dd, *J*_2,3_ = 11.4 Hz, *J*_2,1_ = 5.4 Hz, 1H, H-2), 4.28 (brt, *J*_5,6*a*_ = *J*_5,6*b*_ = 6.2 Hz, 1H, H-5), 3.94 (dd, *J*_4,3_ = 3.3 Hz, *J*_4,5_ = 1.2 Hz, 1H, H-4), 3.88 (dd, *J*_3,2_ = 11.4 Hz, *J*_3,4_ = 3.3 Hz, 1H, H-3), 3.70, 3.68 (part AX of ABX system, J_AB_ = 11.4 Hz, J_AX_ = 5.6 Hz, 1H, H-6a), 3.66, 3.63 (part BX of ABX system, J_BA_ = 11.4 Hz, J_BX_ = 6.5 Hz, 1H, H-6b). **^13^C NMR** (101 MHz, MeOD) δ 166.2 (CO), 149.4 (q, *J* = 35.0 Hz, C-6′), 148.8 (C-2′), 137.3 (C-4′), 134.4 (C-1″), 133.3 (C-3′), 132.0 (C-2″, C-6″), 128.6 (C-3″, C-5″), 127.1 (C-4″), 120.7 (q, *J* = 277.1 Hz, CF_3_), 120.2 (q, *J* = 2.7 Hz, C-5′), 88.4 (C-1), 72.3 (C-5), 68.8 (C-4), 67.9 (C-3), 61.1 (C-6), 51.8 (C-2). HRMS (ESI) *m*/*z*: calcd for C_19_H_19_F_3_N_2_O_5_S [M+H]^+^ 445.1040; found 445.1072.

Methyl 3,4,6-tri-*O*-acetyl-2-azido-2-deoxy-α-d-galactopyranoside (**44**)

**Procedure e**: The crude was purified by column chromatography (hexane/EtOAc, 0–50%). Yield: 92%; LC–MS (high-pH method): Rt = 0.96 min; *m*/*z* = 363.000 [M+OH]^−^; transparent syrup; ${\left[\alpha \right]}_{D}^{20}$ = +93.9 (*c* 1.31 CH_2_Cl_2_); **^1^H NMR** (400 MHz, CDCl_3_) δ 5.44 (dd, *J*_4,3_ = 3.3 Hz, *J*_4,5_ = 1.3 Hz, 1H, H-4), 5.35 (dd, *J*_3,2_ = 11.1 Hz, *J*_3,4_ = 3.3 Hz, 1H, H-3), 4.90 (d, *J*_1,2_ = 3.5 Hz, 1H, H-1), 4.19 (ddd, *J*_5,6*b*_ = 6.5, *J*_5,6*a*_ = 5.9 Hz, *J*_5,4_ = 1.4 Hz, 1H, H-5), 4.11 (d, *J*_6*a*,5_ = *J*_6*b*,5_= 6.1 Hz, 2H, H-6a, H-6b), 3.71 (dd, *J*_2,3_ = 11.1 Hz, *J*_2,1_ = 3.5 Hz, 1H, H-2), 3.46 (s, 3H, OCH_3_), 2.15 (s, 3H, CH_3_), 2.06 (s, 3H, CH_3_), 2.05 (s, 3H, CH_3_). **^13^C NMR** (101 MHz, CDCl_3_) δ 170.4, 170.0, 169.8 (3 × CO), 98.9 (C-1), 68.4 (C-3), 67.6 (C-4), 66.5 (C-5), 61.7 (C-6), 57.6 (C-2), 55.6 (OCH_3_), 20.7, 20.6, 20.6 (3 × CH_3_). HRMS (ESI) *m*/*z*: calcd for C_13_H_23_N_3_O_8_ [M+NH_4_]^+^ 363.1510; found 363.1536.

Phenyl 3,4,6-tri-*O*-acetyl-2-azido-2-deoxy-α-d-galactopyranoside (**45**)

**Procedure e**: Yield: 67%; LC–MS (high-pH method): Rt = 1.45 min; *m*/*z* = 425.000 [M+OH]^−^; transparent syrup; **^1^H NMR** (400 MHz, CDCl_3_) δ 7.33 (brt, *J* = 8.0 Hz, 2H, H-3″, H-5″), 7.14–7.05 (m, 3H, H-2″, H-4″, H-6″), 5.65 (d, *J*_1,2_ = 3.5 Hz, 1H, H-1), 5.60 (dd, *J*_3,2_ = 11.1 Hz, *J*_3,4_ = 3.3 Hz, 1H, H-3), 5.53 (dd, *J*_4,3_ = 3.3 Hz, *J*_4,5_ = 1.4 Hz, 1H, H-4), 4.36 (brt, *J*_5,6*a*_ = *J*_5,6*b*_ = 6.6 Hz, 1H, H-5), 4.14, 4.11 (part AX of ABX system, *J_AB_* = 10.5 Hz, *J_AX_* = 5.3 Hz, 1H, H-6a), 4.09, 4.07 (part BX of ABX system, *J_BA_* = 10.5 Hz, *J_BX_* = 6.3 Hz, 1H, H-6b), 3.81 (dd, *J*_2,3_ = 11.1 Hz, *J*_2,1_ = 3.5 Hz, 1H, H-2), 2.18 (s, 3H, CH_3_), 2.10 (s, 3H, CH_3_), 1.96 (s, 3H, CH_3_). **^13^C NMR** (101 MHz, CDCl_3_) δ 170.3, 170.0, 169.9 (3 × CO), 156.2 (C-1″), 129.7 (C-3″, C-5″), 123.3 (C-4″), 116.9 (C-2″, C-6″), 97.1 (C-1), 68.2 (C-4), 67.5 (C-5), 67.4 (C-3), 61.4 (C-6), 57.3 (C-2), 20.7, 20.6, 20.6 (3 × CH_3_). Data are in full agreement with those previously reported [27].

But-3-yn-1-yl 3,4,6-tri-*O*-acetyl-2-azido-2-deoxy-α-d-galactopyranoside (**46**)

**Procedure e**: Yield: 61%; LC–MS (high-pH method): Rt = 1.06 min; *m*/*z* = 401.000 [M+OH]^−^; transparent syrup; ${\left[\alpha \right]}_{D}^{20}$ = +118.0 (*c* 1.00 CH_2_Cl_2_); **^1^H NMR** (400 MHz, CDCl_3_) δ 5.46 (dd, *J*_4,3_ = 3.3 Hz, *J*_4,5_ = 1.4 Hz, 1H, H-4), 5.38 (dd, *J*_3,2_ = 11.1 Hz, *J*_3,4_ = 3.3 Hz, 1H, H-3), 5.05 (d, *J*_1,2_ = 3.5 Hz, 1H, H-1), 4.33 (brt, *J*_5,6a_ = *J*_5,6b_ = 6.5 Hz, 1H, H-5), 4.14, 4.11 (part AX of ABX system, *J_AB_* = 11.3 Hz, *J_AX_* = 6.3 Hz, 1H, H-6a), 4.09, 4.07 (part BX of ABX system, *J_BA_* = 11.3 Hz, *J_BX_* = 6.9 Hz, 1H, H-6b), 3.78 (m, 2H, H-1a″, H-1b″), 3.65 (dd, *J*_2,3_ = 11.1 Hz, *J*_2,1_ = 3.5 Hz, 1H, H-2), 2.56 (td, *J* = 6.8, 2.7 Hz, 2H, H-2a″, H-2b″), 2.15 (s, 3H, CH_3_), 2.06 (s, 6H, 2 × CH_3_), 2.02 (t, *J* = 2.6 Hz, 1H, H-4″). **^13^C NMR** (101 MHz, CDCl_3_) δ 170.4, 170.0, 169.8 (3 × CO), 98.3 (C-1), 80.5 (C-3″), 69.9 (C-4″), 68.2 (C-3), 67.6 (C-4), 67.0 (C-1″), 66.9 (C-5), 61.7 (C-6), 57.4 (C-2), 20.7, 20.6, 20.6 (3 × CH_3_), 19.8 (C-2″). HRMS (ESI) *m*/*z*: calcd for C_16_H_21_N_3_O_8_ [M+H]^+^ 384.1401; found 384.1422.

Methyl 2-acetamido-3,4,6-tri-*O*-acetyl-2-deoxy-α-d-galactopyranoside (**47**)

**Procedure d**: Starting material: **44**. The crude was purified by column chromatography (hexane/EtOAc, 0–80%). Yield: 77%; LC–MS (high-pH method): Rt = 0.64 min; *m*/*z* = 362.000 [M+H]^+^; transparent syrup; ${\left[\alpha \right]}_{D}^{20}$ = +183.1 (*c* 1.60 CH_2_Cl_2_); **^1^H NMR** (400 MHz, CDCl_3_) δ 6.01 (d, *J*_*N*,2_ = 9.5 Hz, 1H, NH), 5.37 (d, *J*_4,3_ = 3.4 Hz, 1H, H-4), 5.15 (dd, *J*_3,2_ = 11.4 Hz, *J*_3,4_ = 3.3 Hz, 1H, H-3), 4.80 (d, *J*_1,2_ = 3.6 Hz, 1H, H-1), 4.57 (ddd, *J*_2,3_ = 11.3 Hz, *J*_2,*NH*_ = 9.5 Hz, *J*_2,1_ = 3.6 Hz, 1H, H-2), 4.21–4.04 (m, 3H, H-6a, H-6b, H-5), 3.41 (s, 3H, OCH_3_), 2.16 (s, 3H, CH_3_(NHCO)), 2.05 (s, 3H, CH_3_), 1.99 (s, 3H, CH_3_), 1.98 (s, 3H, CH_3_). **^13^C NMR** (101 MHz, CDCl_3_) δ 170.7, 170.3, 170.2, 170.1 (4 × CO), 98.7 (C-1), 68.3 (C-3), 67.7 (C-4), 66.5 (C-5), 61.9 (C-6), 55.4 (OCH_3_), 47.7 (C-2), 23.1 (NHCOCH_3_), 20.6, 20.6, 20.5 (3 × CH_3_). HRMS (ESI) *m*/*z*: calcd for C_15_H_23_NO_9_ [M+H]^+^ 362.1446; found 362.1470.

Methyl 2-acetamido-2-deoxy-α-d-galactopyranoside (**48**)

**Procedure f**: Starting material: **47**. Yield: 93%; LC–MS (high-pH method): Rt = 0.15 min; *m*/*z* = 236.000 [M+H]^+^; transparent syrup; ${\left[\alpha \right]}_{D}^{20}$ = +188.2 (*c* 0.17 MeOH); **^1^H NMR** (400 MHz, MeOD) δ 4.59 (d, *J*_1,2_ = 3.7 Hz, 1H, H-1), 4.17 (dd, *J*_2,1_ = 3.7 Hz, *J*_2,3_ = 11.0 Hz, 1H, H-2), 3.78 (d, *J*_4,3_ = 3.3 Hz, 1H, H-4), 3.71–3.56 (m, 4H, H-3, H-5, H-6a, H-6b), 3.27 (s, 3H, OCH_3_), 1.89 (s, 3H, NCOCH_3_). **^13^C NMR** (101 MHz, MeOD) δ 172.6 (CO), 98.7 (C-1), 71.0 (C-3), 69.0 (C-4), 68.5 (C-5), 61.5 (C-6), 54.2 (OCH_3_), 50.2 (C-2), 21.3 (CH_3_). HRMS (ESI) *m*/*z*: calcd for C_9_H_17_NO_6_ [M+H]^+^ 236.1129; found 236.1147.

Methyl 3,4,6-tri-*O*-acetyl-2-benzamido-2-deoxy-α-d-galactopyranoside (**49**)

**Procedure d**: Starting material: **44**. Yield: 47%; ${\left[\alpha \right]}_{D}^{20}$ = +78.7 (*c* 1.50 CH_2_Cl_2_); LC–MS (high-pH method): Rt = 0.94 min; *m*/*z* = 424.000 [M+H]^+^; transparent syrup; **^1^H NMR** (400 MHz, CDCl_3_) δ 7.80–7.72 (m, 2H, H-2′, H-6′), 7.56–7.49 (m, 1H, H-4′), 7.47–7.41 (m, 2H, H-3′, H-5′), 6.46 (d, *J*_NH,2_ = 9.5 Hz, 1H, NH), 5.44 (d, *J*_4,3_ = 3.2 Hz, 1H, H-4), 5.35 (dd, *J*_3,2_ = 11.3 Hz, *J*_3,4_ = 3.3 Hz, 1H, H-3), 4.92 (d, *J*_1,2_ = 3.6 Hz, 1H, H-1), 4.81 (ddd, *J*_2,3_ = 11.2 Hz, *J*_2,NH_ = 9.5 Hz, *J*_2,1_ = 3.6 Hz, 1H, H-2), 4.25–4.07 (m, 3H, H-5, H-6a, H-6b), 3.42 (s, 3H, OCH_3_), 2.19 (s, 3H, CH_3_), 2.07 (s, 3H, CH_3_), 1.96 (s, 3H, CH_3_). **^13^C NMR** (101 MHz, CDCl_3_) δ 171.1, 170.4, 170.3, 167.2 (4 × CO), 133.8 (C-1′), 131.8 (C-4′), 128.6 (C-3′, C-5′), 127.0 (C-2′, C-6′), 98.7 (C-1), 68.4 (C-3), 67.5 (C-4), 66.7 (C-5), 62.0 (C-6), 55.5 (OCH_3_), 48.4 (C-2), 20.8, 20.7, 20.6 (3 × CH_3_). HRMS (ESI) *m*/*z*: calcd for C_20_H_25_NO_9_ [M+H]^+^ 424.1602; found 424.1629.

Methyl 2-benzamido-2-deoxy-α-d-galactopyranoside (**50**)

**Procedure f**: Starting material: **49**. Yield: 87%; LC–MS (high-pH method): Rt = 0.51 min; *m*/*z* = 298.000 [M+H]^+^; transparent syrup; ${\left[\alpha \right]}_{D}^{20}$ = +146.1 (*c* 0.56 MeOH); **^1^H NMR** (400 MHz, MeOD) δ 7.84 (brd, *J* = 8.0 Hz, 2H, H-2′, H-6′), 7.56 (brt, *J* = 7.5 Hz, 1H, H-4′), 7.48 (brt, *J* = 7.5 Hz, 2H, H-3′, H-5′), 4.87 (d, *J*_1,2_ = 3.7 Hz, 1H, H-1), 4.52 (dd, *J*_2,3_ = 10.2 Hz, *J*_2,1_ = 3.7 Hz, 1H, H-2), 3.97 (dd, *J*_3,2_ = 10.2 Hz, *J*_3,4_ = 3.1 Hz, 1H, H-3), 3.96 (d, *J*_4,3_ = 3.1 Hz, 1H, H-4), 3.88–3.72 (m, 3H, H-5, H-6a, H-6b), 3.42 (s, 3H, OCH_3_). **^13^C NMR** (101 MHz, MeOD) δ 169.6 (CO), 134.4 (C-1′), 131.3 (C-4′), 128.1 (C-3′, C-5′), 127.1 (C-2′, C-6′), 98.7 (C-1), 71.0 (C-5), 69.1 (C-3), 68.1 (C-4), 61.5 (C-6), 54.3 (OCH_3_), 50.9 (C-2). HRMS (ESI) *m*/*z*: calcd for C_14_H_19_NO_6_ [M+H]^+^ 298.1285; found 298.1310.

Phenyl 3,4,6-tri-*O*-acetyl-2-deoxy-2-[6-(trifluoromethyl)nicotinamido]-α-d-galactopyranoside (**51**)

**Procedure d**: Starting material: **45**. Yield: 58%; LC–MS (high-pH method): Rt = 1.27 min; *m*/*z* = 555.200 [M+H]^+^; transparent syrup; ${\left[\alpha \right]}_{D}^{20}$ = +23.8 (*c* 0.21 CH_2_Cl_2_); **^1^H NMR** (400 MHz, CDCl_3_) δ 9.05 (d, *J* = 2.2 Hz, 1H, H-2′), 8.24 (dd, *J* = 8.1, 2.2 Hz, 1H, H-6′), 7.78 (d, *J* = 8.1 Hz, 1H, H-5′), 7.31 (brt, *J* = 8.1 Hz, 2H, H-2″, H-6″), 7.12–6.99 (m, 3H, H-3″, H-4″, H-5″), 6.74 (d, *J*_*NH*,2_ = 9.1 Hz, 1H, NH), 5.76 (d, *J*_1,2_ = 3.6 Hz, 1H, H-1), 5.59 (dd, *J*_3,2_ = 11.3 Hz, *J*_3,4_ = 3.3 Hz, 1H, H-3), 5.52 (d, *J*_4,3_ = 3.1 Hz, 1H, H-4), 4.95 (ddd, *J*_2,3_ = 11.3 Hz, *J*_2,*NH*_ = 9.1 Hz, *J*_2,1_ = 3.6 Hz, 1H, H-2), 4.37 (brt, *J*_5,6*a*_ = *J*_5,6*b*_ = 6.6 Hz, 1H, H-5), 4.16, 4.14 (part AX of ABX system, J_AB_ = 11.3 Hz, J_AX_ = 5.9 Hz, 1H, H-6a), 4.09, 4.07 (part BX of ABX system, J_BA_ = 11.3 Hz, J_BX_ = 7.3 Hz, 1H, H-6b), 2.23 (s, 3H, CH_3_), 2.04 (s, 3H, CH_3_), 1.94 (s, 3H, CH_3_). **^13^C NMR** (101 MHz, CDCl_3_) δ 171.6, 170.3, 170.2, 164.3 (4 × CO), 156.0 (C-1″), 150.7 (q, *J* = 34.9 Hz, C-6′), 148.4 (C-2′), 136.7 (C-4′), 131.8 (C-3′), 129.8 (C-2″, C-6″), 123.4 (C-4″), 121.0 (q, *J* = 274.6 Hz, CF_3_), 120.5 (q, *J* = 3.0 Hz, C-5′), 116.7 (C-3″, C-5″), 96.3 (C-1), 68.3 (C-3), 67.8 (C-5), 67.2 (C-4), 61.6 (C-6), 49.2 (C-2), 20.8, 20.7, 20.6 (3 × CH_3_). HRMS (ESI) *m*/*z*: calcd for C_25_H_25_F_3_N_2_O_9_ [M+H]^+^ 555.1585; found 555.1633.

Phenyl 2-deoxy-2-[6-(trifluoromethyl)nicotinamido]-α-d-galactopyranoside (**52**)

**Procedure f**: Starting material: **51**. Yield: 93%; LC–MS (high-pH method): Rt = 0.88 min; *m*/*z* = 451.000 [M+Na]^+^; transparent syrup; ${\left[\alpha \right]}_{D}^{20}$ = +118.3 (*c* 0.11 MeOH); **^1^H NMR** (400 MHz, MeOD) δ 9.01 (d, *J* = 2.2 Hz, 1H, H-2′), 8.31 (dd, *J* = 8.1, 2.2 Hz, 1H, H-4′), 7.81 (d, *J* = 8.2 Hz, 1H, H-5′), 7.16 (tt, *J* = 7.4, 2.2 Hz, 2H, H-2″, H-6″), 7.04 (brd, *J* = 8.5 Hz, 2H, H-3″, H-5″), 6.90 (brt, 1H, H-4″), 5.56 (d, *J*_1,2_ = 3.7 Hz, 1H, H-1), 4.57 (dd, *J*_2,3_ = 11.1 Hz, *J*_2,1_ = 3.7 Hz, 1H, H-2), 4.12 (dd, *J*_3,2_ = 11.1 Hz, *J*_3,4_ = 3.2 Hz, 1H, H-3), 3.97–3.90 (m, 2H, H-4, H-5), 3.68, 3.66 (part AX of ABX system, J_AB_ = 11.6 Hz, J_AX_ = 5.8 Hz, 1H, H-6a), 3.64, 3.62 (part BX of ABX system, J_BA_ = 11.4 Hz, J_BX_ = 6.5 Hz, 1H, H-6b). **^13^C NMR** (101 MHz, MeOD) δ 167.8 (CO), 158.8 (C-1″), 150.6 (q, *J* = 34.9 Hz, C-6′), 150.2 (C-2′), 138.6 (C-4′), 134.7 (C-3′), 130.6 (C-2″, C-6″), 129.1 (q, *J* = 271.9 Hz, CF_3_), 123.7 (C-4″), 121.6 (q, *J* = 2.8 Hz, C-5′), 116.9 (C-3″, C-5″), 98.4 (C-1), 73.3 (C-5), 70.1 (C-4), 69.1 (C-3), 62.5 (C-6), 52.6 (C-2). HRMS (ESI) *m*/*z*: calcd for C_19_H_19_F_3_N_2_O_6_ [M+H]^+^ 429.1268; found 429.1293.

Phenyl 3,4,6-tri-*O*-acetyl-2-deoxy-2-(3-fluoro-5-methylbenzamido)-α-d-galactopyranoside (**53**)

**Procedure d**: Starting material: **45**. Yield: 71%; LC–MS (high-pH method): Rt = 1.25 min; *m*/*z* = 540.200 [M+Na]^+^; transparent syrup; ${\left[\alpha \right]}_{D}^{20}$ = +114.8 (*c* 1.82 CH_2_Cl_2_); **^1^H NMR** (400 MHz, CDCl_3_) δ 7.34–7.21 (m, 4H, H-2″, H-6″, H-2′, H-4′), 7.12–6.99 (m, 4H, H-3″, H-4″, H-5″, H-6′), 6.58 (d, *J*_NH,2_ = 9.3 Hz, 1H, NH), 5.73 (d, *J*_1,2_ = 3.6 Hz, 1H, H-1), 5.58 (dd, *J*_3,2_ = 11.3 Hz, *J*_3,4_ = 3.3 Hz, 1H, H-3), 5.52 (dd, *J*_4,3_ = 3.3 Hz, *J*_4,5_ = 1.3 Hz, 1H, H-4), 4.95 (ddd, *J*_2,3_ = 11.3 Hz, *J*_2,NH_ = 9.3 Hz, *J*_2,1_ = 3.6 Hz, 1H, H-2), 4.36 (brt, *J*_5,6a_ = *J*_5,6b_ = 6.6 Hz, 1H, H-5), 4.15, 4.12 (part AX of ABX system, J_AB_ = 11.5 Hz, J_AX_ = 5.9 Hz, 1H, H-6a), 4.09, 4.06 (part BX of ABX system, J_BA_ = 11.4 Hz, J_BX_ = 7.2 Hz, 1H, H-6b), 2.38 (s, 3H, Ph-CH_3_), 2.22 (s, 3H, CH_3_), 2.02 (s, 3H, CH_3_), 1.93 (s, 3H, CH_3_). **^13^C NMR** (101 MHz, CDCl_3_) δ 171.3, 170.4, 170.3 (3 × CO), 166.5 (d, *J* = 2.7 Hz, CONH), 162.7 (d, *J* = 247.2 Hz, C-3′), 156.1 (C-1″), 141.2 (d, *J* = 7.7 Hz, C-5′), 135.5 (d, *J* = 7.3 Hz, C-1′), 129.8 (C-2″, C-6″), 123.4 (d, *J* = 2.6 Hz, C-6′), 123.3 (C-4″), 119.5 (d, *J* = 21.1 Hz, C-4′), 116.9 (C-3″, C-5″), 111.4 (d, *J* = 23.1 Hz, C-2′), 96.6 (C-1), 68.3 (C-3), 67.7 (C-5), 67.3 (C-4), 61.8 (C-6), 48.7 (C-2), 21.3 (d, *J* = 1.6 Hz, Ph-CH_3_), 20.8, 20.7, 20.5 (3 × CH_3_). HRMS (ESI) *m*/*z*: calcd for C_26_H_28_FNO_9_ [M+H]^+^ 518.1821; found 518.1860.

Phenyl 2-deoxy-2-(3-fluoro-5-methylbenzamido)-α-d-galactopyranoside (**54**)

**Procedure f**: Starting material: **53**. Yield: 99%; LC–MS (high-pH method): Rt = 0.89 min; *m*/*z* = 392.000 [M+H]^+^; transparent syrup; ${\left[\alpha \right]}_{D}^{20}$ = +123.1 (*c* 0.70 MeOH); **^1^H NMR** (400 MHz, MeOD) δ 7.52 (s, 1H, H-2′), 7.45–7.35 (m, 1H, H-4′), 7.33–7.23 (m, 2H, H-3″, H-5″), 7.18–7.09 (m, 3H, H-2″, H-6″, H-6′), 7.01 (tt, *J* = 7.2, 1.1 Hz, 1H, H-4″), 5.65 (d, *J*_1,2_ = 3.7 Hz, 1H, H-1), 4.63 (dd, *J*_2,3_ = 11.1 Hz, *J*_2,1_ = 3.7 Hz, 1H, H-2), 4.23 (dd, *J*_3,2_ = 11.1 Hz, *J*_3,4_ = 3.2 Hz, 1H, H-3), 4.09–4.01 (m, 2H, H-4, H-5), 3.83–3.70 (m, 2H, H-6a, H-6b), 2.41 (s, 3H, Ph-CH_3_). **^13^C NMR** (101 MHz, MeOD) δ 168.5 (d, *J* = 2.9 Hz, CONH), 162.5 (d, *J* = 245.2 Hz, C-3′), 157.6 (C-1″), 140.9 (d, *J* = 7.7 Hz, C-1′), 136.3 (d, J = 7.9 Hz, C-5′), 129.1 (C-2″, C-6″), 123.6 (d, *J* = 2.6 Hz, C-2′), 122.2 (C-4″), 118.47 (d, *J* = 21.5 Hz, C-6′), 116.9 (C-3″, C-5″), 111.1 (d, *J* = 23.3 Hz, C-4′), 97.1 (C-1), 71.9 (C-5), 68.8 (C-4), 67.6 (C-3), 61.1 (C-6), 51.1 (C-2), 19.8 (Ph-CH_3_). HRMS (ESI) *m*/*z*: calcd for C_20_H_22_FNO_6_ [M+H]^+^ 392.1504; found 392.1529.

Phenyl 2-azido-2-deoxy-1-seleno-α-d-galactopyranoside (**55**)

**Procedure f**: Starting material: **2**. Yield: 98%; LC–MS (high-pH method): Rt = 0.77 min; *m*/*z* = 363.000 [M+OH]^−^; yellow syrup; ${\left[\alpha \right]}_{D}^{20}$ = +238.4 (*c* 2.37 MeOH); **^1^H NMR** (400 MHz, MeOD) δ 7.69–7.59 (m, 2H, H-2″, H-6″), 7.35–7.22 (m, 3H, H-3″, H-5″, H-4″), 5.94 (d, *J*_1,2_ = 5.3 Hz, 1H, H-1), 4.23 (ddd, *J*_5,6a_ = *J*_5,6b_ = 6.2 Hz, *J*_5,4_ = 1.3 Hz, 1H, H-5), 4.06 (dd, *J*_2,3_ = 10.4 Hz, *J*_2,1_ = 5.3 Hz, 1H, H-2), 3.96 (dd, *J*_4,3_ = 3.2 Hz, *J*_4,5_ = 1.3 Hz, 1H, H-4), 3.79–3.66 (m, 2H, H-3, H-6a), 3.60 (dd, *J*_6b,6a_ = 11.4 Hz, *J*_6b,5_ = 6.4 Hz, 1H, H-6b). **^13^C NMR** (101 MHz, MeOD) δ 136.0 (C-2″, C-6″), 130.1 (C-1″), 130.0 (C-3″, C-5″), 128.8 (C-4″), 87.4 (C-1), 74.9 (C-5), 72.5 (C-3), 70.2 (C-4), 63.1 (C-2), 62.0 (C-6). HRMS (ESI) *m*/*z*: calcd for C_12_H_15_N_3_O_4_SeNa [M+Na]^+^ 368.0121; found 368.0145.

Phenyl 3-*O*-acetyl-2-azido-4,6-*O*-benzylidene-2-deoxy-1-seleno-α-d-galactopyranoside (**56**)

Compound **55** (0.668 mmol) was dissolved in dry acetonitrile (7.4 mL). Then dimethoxymethylbenzene (2.5 eq), TsOH∙H_2_O (0.3 eq.) were added. The reaction mixture was stirred at 80 °C, for 20 min. After cooling down, the mixture was neutralized with triethylamine, evaporated and the crude was dissolved in DCM, 4 Å molecular sieves were added. Then, triethylamine (3.0 eq.) was added, followed by the addition of acetyl chloride (3.0 eq.) dropwise. The reaction mixture was stirred for 20 min, at room temperature. The mixture was washed with water (3 × 15 mL) and saturated NaHCO_3_ (3 × 15 mL) and extracted with DCM. The organic layer was combined and dried over MgSO_4,_ filtered and concentrated under reduced pressure. Compound purification was accomplished with preparative HPLC. Yield: 73%; LC–MS (high-pH method): Rt = 1.37 min; *m*/*z* = 493.000 [M+OH]^−^; yellow syrup; **^1^H NMR** (400 MHz, CDCl_3_) δ 7.62–7.53 (m, 2H, H-2″, H-6″), 7.52–7.45 (m, 2H, H-2′, H-6′), 7.42–7.32 (m, 3H, H-3′, H-4′, H-5′), 7.30–7.23 (m, 3H, H-3″, H-4″, H-5″), 6.08 (d, *J*_1,2_ = 5.2 Hz, 1H, H-1), 5.54 (s, 1H, PhCH), 5.06 (dd, *J*_3,2_ = 10.9 Hz, *J*_3,4_ = 3.3 Hz, 1H, H-3), 4.53–4.48 (m, 2H, H-2, H-4), 4.17–4.00 (m, 3H, H-5, H-6a, H-6b), 2.16 (s, 3H, CH_3_). **^13^C NMR** (101 MHz, CDCl_3_) δ 170.4 (CO), 137.4 (C-1′), 134.0 (C-2″, C-6″), 129.3 (C-3′, C-5′), 129.2 (C-4′), 128.4 (C-1″), 128.3 (C-3″, C-5″), 127.9 (C-4″), 126.2 (C-2′, C-6’), 100.9 (PhCH), 84.9 (C-1), 73.0 (C-4), 72.5 (C-3), 69.0 (C-6), 64.8 (C-5), 58.4 (C-2), 21.0 (CH_3_).

Methyl 3-*O*-acetyl-2-azido-4,6-*O*-benzylidene-2-deoxy-α/β-d-galactopyranoside (**57**)

**Procedure d**: Starting material: **56**. Crude was analyzed by NMR, affording α:β (1:2). Yield: 53% (α+β); **^1^H NMR** (400 MHz, CDCl_3_) (α+β) δ 7.54–7.46 (m, H-Ph), 7.44–7.31 (m, H-Ph), 7.28–7.25 (m, H-Ph), 5.54 (s, PhCH(α)), 5.52 (s, PhCH(β)), 5.30 (dd, *J*_3,2_ = 11.1 Hz, *J*_3,4_ = 3.2 Hz, H-3(α)), 4.96 (d, *J*_1,2_ = 3.4 Hz, H-1(α)), 4.73 (dd, *J*_3,2_ = 10.9 Hz, *J*_3,4_ = 3.5 Hz, H-3(β)), 4.47 (d, *J*_4,3_ = 3.2 Hz, H-4(α)), 4.38–4.32 (m, H-4(β), H-6aβ)), 4.31–4.26 (m, H-1(β), H-6a(α)), 4.11–4.04 (m, H-6b(α), H-6b(β)), 4.00 (dd, *J*_2,3_ = 11.1 Hz, *J*_2,1_ = 3.4 Hz, H-2(α)), 3.90 (dd, *J*_2,3_ = 10.8 Hz, *J*_2,1_ = 8.0 Hz, H-2(β)), 3.78–3.74 (m, H-5(α)), 3.61 (s, OCH_3_(β)), 3.50–3.47 (m, H-5(β)), 3.46 (s, OCH_3_(α)), 2.15 (s, COCH_3_(β)), 1.61 (s, COCH_3_(α)). **^13^C NMR** (α+β) (101 MHz, CDCl_3_) δ 170.7 (CO(β)), 170.7 (CO(α)), 137.6 (C-Ph(α)), 137.5 (C-Ph(β)), 129.3 (C-Ph(β)), 129.2 (C-Ph(α)), 128.4 (C-Ph(α)), 128.3 (C-Ph(β)), 126.4 (C-Ph(β)), 126.3 (C-Ph(α)), 103.1 (C-1(β)), 101.1 (PhCH(β)), 100.9 (PhCH(α)), 99.5 (C-1(α)), 73.6 (C-4(α)), 72.8 (C-4(β)), 72.5 (C-3(β)), 69.9 (C-3(α)), 69.3 (C-6(α)), 69.1 (C-6(β)), 66.4 (C-5(β)), 62.5 (C-5(α)), 60.4 (C-2(β)), 57.5 (C-2(α)), 57.3 (OCH_3_(β)), 55.8 (OCH_3_(α)), 21.2 (COCH_3_(α)), 21.1 (COCH_3_(β)). HRMS (ESI) *m*/*z*: calcd for C_16_H_19_N_3_O_6_ [M+H]+ 350.1347; found 350.1375. Data are in full agreement with those previously reported [28].

Methyl 2-acetamido-3,4,6-tri-*O*-acetyl-2-deoxy-β-d-galactopyranoside (**58**)

**Procedure d**: Starting material: **46**. The crude was purified by column chromatography (hexane/EtOAc, 0–50%). Yield: 44%; LC–MS (high-pH method): Rt = 0.59 min; *m*/*z* = 362.200 [M+H]^+^; transparent syrup; ${\left[\alpha \right]}_{D}^{20}$ = −6.9 (*c* 1.01 CH_2_Cl_2_); **^1^H NMR** (400 MHz, CDCl_3_) δ 5.60 (d, *J*_NH,2_ = 8.7 Hz, 1H, NH), 5.37 (dd, *J*_4,3_ = 3.4 Hz, *J*_4,5_ = 1.2 Hz, 1H, H-4), 5.29 (dd, *J*_3,2_ = 11.2 Hz, *J*_3,4_ = 3.4 Hz, 1H, H-3), 4.63 (d, *J*_1,2_ = 8.4 Hz, 1H, H-1), 4.21, 4.18 (part AX of ABX system, *J*_AB_ = 11.2 Hz, *J*_AX_ = 6.5 Hz, 1H, H-6a), 4.15, 4.12 (part BX of ABX system, *J*_BA_ = 11.3 Hz, *J*_BX_ = 6.8 Hz, 1H, H-6b), 4.04–3.89 (m, 2H, H-2, H-5), 3.52 (s, 3H, OCH_3_), 2.15 (s, 3H, CH_3_), 2.06 (s, 3H, CH_3_), 2.01 (s, 3H, CH_3_), 1.97 (s, 3H, CH_3_). **^13^C NMR** (101 MHz, CDCl_3_) δ 170.6, 170.5, 170.4, 170.3 (4 × CO), 101.8 (C-1), 70.6 (C-5), 69.9 (C-3), 66.8 (C-4), 61.5 (C-6), 56.8 (OCH_3_), 51.5 (C-2), 23.5 (NHCOCH_3_), 20.7, 20.7, 20.6 (3 × CH_3_). HRMS (ESI) *m*/*z*: calcd for C_15_H_23_NO_9_ [M+H]^+^ 362.1446; found 362.1470.

### 3.2. Preparation of Aβo

Aβ_1–42_ film preparation: 16 mg portions of lyophilized human Aβ_1–42_ protein fragment were removed from storage at −80 °C 30 min prior to use. Each Aβ portion was resuspended in hexafluoro-2-propanol (HFIP) to produce a 4.5 mg/mL Aβ solution, followed by incubation at room temperature for 30 min. The solutions were then aliquoted (110 µL in LoBind microcentrifuge tubes, Eppendorf, Hamburg, Germany), centrifuged at 14,500× *g* for 5 min, and the peptides dried under a nitrogen atmosphere to remove HFIP for 1 h. The dried peptide films were stored at −20 °C in a desiccator jar. Aβ oligomers (Aβo) preparation: Aβ_1–42_ peptide films were removed from the freezer 30 min prior to use and were equilibrated at 0 °C. 5 mL HiTrap™ desalting columns (GE Healthcare, Chicago, IL, USA) were carefully equilibrated with 20 mL of Neurobasal™ Medium 1x (Gibco by Life Technologies, Paisley, UK). Meanwhile, each peptide film was resuspended in 250 µL of DMSO to yield a final Aβ concentration of 10 mM. DMSO was removed by passing 6 peptide solutions through the HiTrap™ column (GE Healthcare, Chicago, IL, USA) and washing it twice with 1 mL of Neurobasal™ medium (Gibco by Life Technologies, Paisley, UK). The eluted peptide was collected into two LoBind microcentrifuge tubes (Eppendorf, Hamburg, Germany). Protein concentration was assessed by using the Bradford Protein Assay kit (Thermo Fisher Scientific, Waltham, MA, USA), and each tube was normalized with Neurobasal™ Medium (Gibco by Life Technologies, Paisley, UK) to a final concentration of 50 µM. The normalized Aβ_1–42_ monomers were then oligomerized for 1 h at 25 °C using a plate shaker. The oligomers were centrifuged at 14,500× *g* for 10 min, the supernatant was collected and analyzed in a DynaPro^®^ DLS instrument (Wyatt Technology, Santa Barbara, CA, USA), and the results analyzed using Dynamics V7 Software. The freshly prepared Aβo were kept at −80 °C until needed for STD NMR experiments.

### 3.3. Affinity-Selection Mass Spectrometry (ASMS) by Ultrafiltration-Centrifugation

Binding experiments were performed using Amicon Ultra-0.5 centrifugal filters (10 kDa MWCO, Millipore, Billerica, MA, USA). Filters were pre-conditioned with PBS (400 μL, pH 7.4) by centrifugation (15,000 rpm, 20 min, 4 °C). Compound–protein incubation mixtures were then prepared: (i) compound solution (1 μM, 344 μL) with PBS (16 μL) as the no-protein control (NP), and (ii) compound solution (1 μM, 344 μL) with protein (50 μM stock, 16 μL; final volume 360 μL; final protein concentration 2 μM) as the protein sample (P). Mixtures were homogenized by pipetting and vortexing, then centrifuged (15,000 rpm, 20 min, 4 °C). In parallel, 1 mL of compound solution (1 μM) was reserved as pre-spin LC–MS control (C). After removal of the filtrates, two sequential washes with PBS (360 μL each) were performed under identical conditions. Bound ligands were eluted by inverting the filters into clean receivers containing DMSO (80 μL) and centrifuging at 6000 rpm for 3 min at room temperature. Eluates (P and NP) and the pre-spin control (C) were collected in HPLC vials for LC–MS analysis. As assay validation, carbonic anhydrase with ethoxzolamide was used as positive control. A relative response above 5% was considered indicative of binding. Relative binding response (%) was calculated according to Equation (1).

(1) $$Rel.\;response\;(\%)=\frac{{Peak\;area}_{P}}{Peak\;{area}_{C}}\times 100\;\;\;\;$$

### 3.4. STD-NMR and ^19^F NMR Screening Assay Against Aβo

All samples were prepared in 550 µL of deuterated 10 mM phosphate buffer. Aβo and test compounds were added to reach final concentrations of 2 and 200 µM, respectively (1:100 molar ratio). The final percentage of DMSO was 2% in screening experiments, and 4% in the competition assays. Controls were prepared in the absence of Aβo, but maintaining the same relative volumes of buffer, Neurobasal Medium and DMSO. All experiments were performed on a Bruker Avance III 600 MHz spectrometer (Bruker Biospin, Rheinstetten, Germany) equipped with a quadruple resonance (QCI) cryoprobe. For the acquisition, a standard Bruker pulse sequence was used, with 2 s of saturation at 0 ppm (on-resonance) and −40 ppm (off-resonance) for on and off resonance. Double solvent suppression was achieved with a double pulsed field gradient spin echo (DPFGSE) element incorporating a selective 180 pulse designed to flip both the water (from buffer) and DMSO (from ligand stock) peaks. Typically, 128 scans were acquired prior to processing with a 1 Hz line broadening function. Data was processed in Topspin 3.6 software using the stdsplit AU program (Bruker Biospin, Rheinstetten, Germany). ^19^F CPMG experiments were performed using a standard pulse sequence with ^1^H decoupling during acquisition and a CPMG relaxation delay of 400 ms. Typically, 128 scans were acquired. Spectra were processed with 1 Hz exponential line broadening and peak areas extracted using a custom Python script for TopSpin 3.6 software.

### 3.5. Rapid Equilibrium Dialysis (RED) Experiments

RED assays were carried out using two-chamber dialysis devices from Thermo Fisher Scientific. The white chambers were filled with 500 μL of phosphate buffer (pH 7.4), while each red chamber received 288 μL of ligand solution (1 μM). To one of the red chambers, 12 μL of freshly prepared Aβ_1–42_ oligomers (50 μM) were added, yielding a final protein concentration of 2 μM (protein-containing sample, P). The corresponding control chamber received 12 μL of Neurobasal™ medium (no protein, NP) (Gibco by Life Technologies, Paisley, UK). The devices were shaken for 4 h at room temperature, after which aliquots were collected from the buffer chambers and analyzed by selective ion monitoring (SIM) LC–MS. Each experiment was performed in triplicate. Binding (%) was calculated according to Equation (2).

(2) $$Binding\;\left(\%\right)=\left[1-\frac{SIM(P)}{SIM(NP)}\right]\times 100\;\;\;\;\;$$

### 3.6. Microsomal Metabolism Assay

Microsomal metabolism studies were performed in a single-point format and analyzed by LC–MS (Advion Interchim Scientific platform). Microsomal stability of test compounds was evaluated in liver microsomes from human, mouse, rat, and dog (final protein concentration: 1.1 mg/mL), using an NADPH-regenerating system (1.3 mM NADPH, 3.3 mM glucose-6-phosphate, 0.4 U/mL glucose-6-phosphate dehydrogenase, 3.3 mM MgCl_2_, in phosphate buffer pH 7.4). Reactions were initiated by addition of the NADPH-regenerating system. Incubations were performed in 96-well plates (total volume 500 µL) at 37 °C with 4 µM test compound and 0.02% DMSO final concentration. After 0 and 30 min, aliquots were quenched with ice-cold acetonitrile containing internal standard, centrifuged, and the supernatant analyzed by LC–MS. Positive controls included verapamil and propranolol, which showed the expected high metabolic turnover. No significant compound degradation was observed in control incubations lacking NADPH. Experiments were performed in triplicate. The percentage of metabolized compound was calculated according to Equation (3), where $c_{t=0}$ represents the compound concentration at the start of incubation and $c_{t=30}$ the concentration after 30 min.

(3) $$\%\;Metabolized=\frac{c_{t=0}-c_{t=30}}{c_{t=0}}\times 100\;\;\;\;\;$$

### 3.7. Aqueous Solubility Assays

Aqueous solubility assays were performed at pH 7.4 using a nephelometric light-scattering method (NV-Aqueous pH 7.4 Solubility, Advion, New York, NY, USA). Test compounds were serially diluted (from 1 µM to 200 µM) in phosphate buffer (pH 7.4), in 96-well microtiter plates, and solubility was monitored with a Labsystems Nepheloskan Ascent (Thermo Fisher Scientific) under the following conditions: lamp voltage 10 V, photomultiplier tube voltage 300 V, final DMSO concentration 0.02%, and room temperature. Amitriptyline and terfenadine were included as positive controls. Solubility was expressed as the first insoluble concentration (µM), corresponding to the lowest concentration at which precipitation was detected, and the last soluble concentration (µM), the highest concentration at which compounds remained fully soluble. Based on the latter, solubility was classified as high (≥100 µM), moderate (50–100 µM), or low (<50 µM). Blank buffer measurements were subtracted prior to analysis. All experiments were carried out in triplicate, and results are reported as mean values.

### 3.8. Cell Culture

Human embryonic kidney (HEK) cells were grown in Dulbecco’s Modified Eagle Medium (DMEM; D5796, Sigma-Aldrich, St. Louis, MO, USA) supplemented with 10% fetal bovine serum (FBS; 10695023, Thermo Fisher Scientific), 1% penicillin-streptomycin (10452882, Thermo Fisher Scientific) and 4 mM L-glutamine (G7513, Sigma) and maintained at 37 °C in a 5% CO_2_ atmosphere.

### 3.9. Immunocytochemistry Analysis of Compound-Mediated Aβo–PrP^C^ Disruption

96-well plates were incubated with 50 µL of poly-L-ornithine hydrobromide 100 µg/mL for 40 min and washed with PBS 3 times. HEK cells were seeded onto the plates diluted in Phenol red-free DMEM medium with high glucose (Gibco) to achieve 2 × 10^4^ cells per 100 µL in each well. The cells were incubated at 37 °C with 5% CO_2_ overnight. Then, the medium was removed and 50 µL of conditioned medium with recombinant Aβo prepared by the Sheffield Institute for Translational Neuroscience (SiTraN) 1000 pc/mL was added to each well. These oligomers were derived from Chinese Hamster Ovary cells (7PA2 cells) stably transfected with cDNA encoding APP751, an amyloid precursor protein that contains the Val717Phe familial Alzheimer’s disease mutation, as previously described by Kittelberger et al. [29]. After a 2 h incubation period, the conditioning medium was removed, and the cells were washed once with PBS. Then, 100 µL of fresh Phenol red-free DMEM medium with high glucose were added to each well and the cells were incubated with the phenylselenogalactosides derivatives at 10 µM in 0.5% DMSO for 1 h. The medium was subsequently removed. The cells were washed with 100 µL of PBS containing Mg^2+^ and Ca^2+^, after which 100 µL of 4% PFA were added to each well. Once incubated for 15 min at room temperature, the PFA was removed, and the cells were washed once again with 100 µL of PBS containing Mg^2+^ and Ca^2+^. 100 µL of phosphate-buffered saline with Tween (PBS-T) with 5% of Donkey serum were added and the cells were incubated for 1 h at room temperature. Then, the blocking solution was removed and the primary antibody (anti-Aβo 6E10 antibody prepared in 50 µL of PBS-T with 5% of Donkey serum, 1:250 dilution) was added prior to overnight incubation at 4 °C. After removal of the primary antibody, the cells were washed 3 times with 50 µL of PBS-T for 5 min at room temperature. 50 µL of the secondary antibody (Alexa Fluor™ 594 anti-mouse antibody, prepared in PBS-T, 1:500 dilution) were subsequently added to each well and the cells were incubated another hour at room temperature. The liquid was aspirated, and the cells were washed twice with PBS-T and once with PBS (50 µL each wash). 50 µL of 4′,6-diamidino-2-phenylindole (DAPI, 100 ng/mL in PBS) were added to each well. Following 5 min of incubation, DAPI was removed and the cells washed with 100 µL of PBS 3 times. 100 µL of PBS were finally added and the imaging was carried out by The Wolfson Light Microscopy Facility, using ImageXpress Micro Widefield High Content Screening System, 20× magnification, and 30 pictures taken per well. Data analysis was executed using MetaXpress Software Multi-Wavelength Translocation Application Module (version 6.5, Molecular Devices, Sunnyvale, CA, USA). Results are presented as the average of two independent experiments performed in triplicate.

### 3.10. MTT Cytotoxicity Assay

HEK cells were seeded onto a 96-well plate at a density of 1 × 10^4^ cells per well and incubated at 37 °C with 5% CO_2_ overnight prior to the assay. Each compound was added in DMSO to reach final concentrations of 1 μM, 5 μM, 10 μM, 20 μM and 50 μM, in triplicate, adjusting the final DMSO concentration to 0.5%. After 24 h of incubation at 37 °C in 5% CO_2_, 10 μL of 3-(4,5-dimethylthiazol-2-yl)-2,5-diphenyltetrazolium bromide (MTT, 10 mg/mL in PBS) were added to each well, and cells were incubated for another 2 h. Then, the medium was removed and the blue formazan crystals dissolved in 60 μL of acidified isopropanol. The plates were shaken to promote full dissolution of the crystals, after which the optical density (OD) at 570 nm (with a 690 nm reference filter) was measured using a microplate reader. Cell survival (%) was calculated using Equation (4), and the results presented as the average of two experiments performed in triplicate.

(4) $$Cell\;Survival\;\left(\%\right)=\left[\frac{{OD}_{sample}-{OD}_{medium}}{{OD}_{cell\;control}-{OD}_{medium}}\right]\times 100\;\;\;\;\;$$

## 4. Conclusions

In this work, a modular and efficient synthetic strategy enabled the preparation of a structurally diverse library of phenyl 2-acetamidoselenogalactoside mimetics, through a systematic variation at the C2 position and at the anomeric heteroatom (Se, S, O). The resulting compounds were evaluated for their interactions with Aβ_1–42_ oligomers (Aβo) using complementary biophysical techniques, including STD-NMR, 19F-NMR and RED assays, supported by ADME and cytotoxicity studies. The results indicate that several compounds within this series interact with Aβo in a weak-binding regime, consistent with the transient and multivalent nature of ligand recognition by dynamic amyloid assemblies. Notably, multiple derivatives (e.g., **10**, **12**, **16**, **18**, **20**, **34**) exhibited clear STD-NMR responses, highlighting that interaction is not restricted to a single structure but rather depends on a combination of structural features. Structure–activity relationship analysis revealed that the presence of an aromatic substituent at C2 is required, potentially enhancing π-π interactions, but not sufficient for binding. Heteroaromatic substituents are also tolerated, although with variable behavior, with some derivatives exhibiting interaction (e.g., **20** and **36**), while others do not (e.g., **28** and **32**), indicating that subtle differences in electronic distribution and geometry influence the interaction process. The nature of the C2 functionalization also plays a key role, where amide and especially urea favor interactions, likely due to increased hydrogen-bonding capacity and conformational restriction.

The selenium atom, at the anomeric position, was also found to enhance interaction compared to sulphur and oxygen analogs, consistent with its higher polarizability and ability to support transient intermolecular contacts. Furthermore, deacetylated derivatives displayed improved aqueous solubility and maintained interaction, reinforcing their relevance as more suitable candidates for further development.

Within this context, compound **34** emerges as the most promising lead, combining favorable physicochemical properties, metabolic stability, low cytotoxicity, and consistent interaction with Aβo, as well as measurable interference with Aβo–PrP^C^ interactions. Overall, this study identifies phenyl 2-acetamidoselenogalactosides as a new class of glycomimetics capable of interacting with amyloid assemblies, supporting the growing interest in selenium-containing glycomimetics as modulators of amyloid aggregation processes [8].

## Figures and Tables

**Table 1:** Library of phenyl 2-acetamidoselenogalactoside mimetics synthesized.

Compound		Yield (%)	Compound		Yield (%)
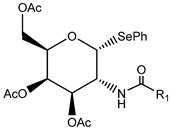			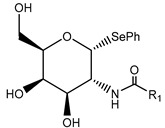		
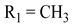	(**3**)	62 ^1^/66 ^2^	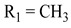	(**4**)	98
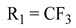	(**5**)	90 ^3^/83 ^2^	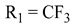	(**6**)	95
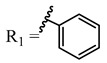	(**7**)	69 ^1^/66 ^2^	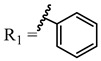	(**8**)	99
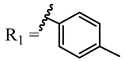	(**9**)	42	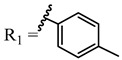	(**10**)	98
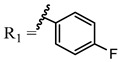	(**11**)	43	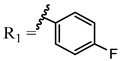	(**12**)	94
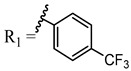	(**13**)	56	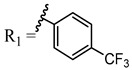	(**14**)	97
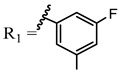	(**15**)	61	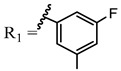	(**16**)	87
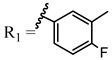	(**17**)	73	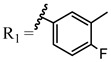	(**18**)	93
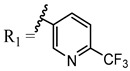	(**19**)	49	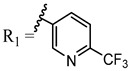	(**20**)	93
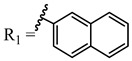	(**21**)	80	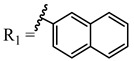	(**22**)	93
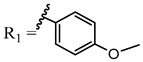	(**23**)	41	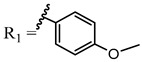	(**24**)	97
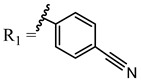	(**25**)	46	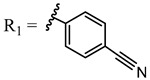	(**26**)	98
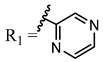	(**27**)	40	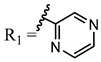	(**28**)	97
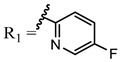	(**29**)	35	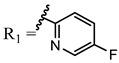	(**30**)	78
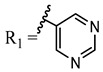	(**31**)	38	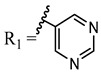	(**32**)	94
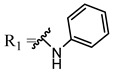	(**33**)	67	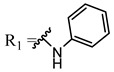	(**34**)	90
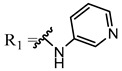	(**35**)	36	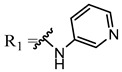	(**36**)	87
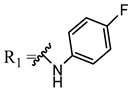	(**37**)	41	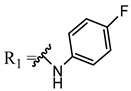	(**38**)	92
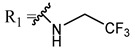	(**39**)	51	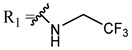	(**40**)	97

^1^ Scheme 1**b**; ^2^ Scheme 1**d**; ^3^ Scheme 1**c**.

**Table 2:** Library of phenyl 2-acetamidogalactoside mimetics synthesized.

Compound		Yield (%)	Compound		Yield (%)
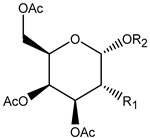			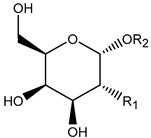		
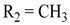	(**44**)	92 ^1^	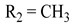	(**48**)	93 ^3^
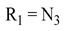			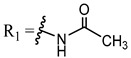		
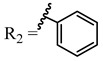	(**45**)	67 ^1^	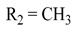	(**50**)	87 ^3^
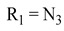			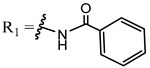		
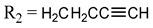	(**46**)	61 ^1^	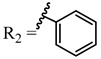	(**52**)	93 ^3^
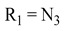			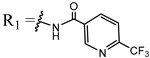		
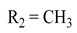	(**47**)	77 ^2^	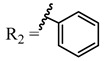	(**54**)	99 ^3^
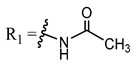			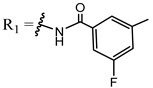		
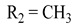	(**49**)	47 ^2^			
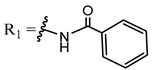					
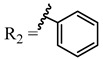	(**51**)	58 ^2^			
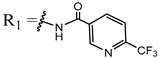					
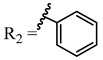	(**53**)	71 ^2^			
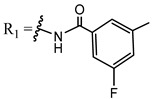					

^1^ Scheme 1**e**; ^2^ Scheme 1**d**; ^3^ Scheme 1**f**.

**Table 3:** STD-NMR screening of deacetylated phenylselenogalactosides against Aβo. (+) positive interaction; (−) negative interaction.

Compound		STD-NMR	Compound		STD-NMR
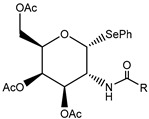		**results**	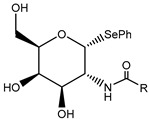		**results**
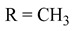	(**4**)	**−**	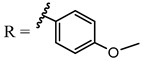	(**24**)	**+**
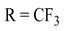	(**6**)	**−**	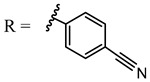	(**26**)	**+**
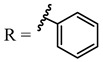	(**8**)	**−**	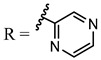	(**28**)	**−**
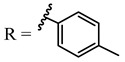	(**10**)	**+**	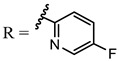	(**30**)	**+**
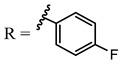	(**12**)	**+**	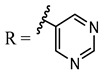	(**32**)	**−**
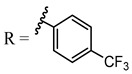	(**14**)	**−**	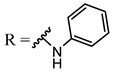	(**34**)	**+**
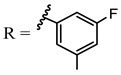	(**16**)	**+**	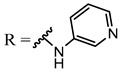	(**36**)	**+**
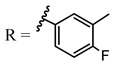	(**18**)	**+**	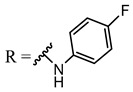	(**38**)	**+**
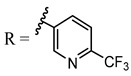	(**20**)	**+**	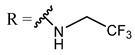	(**40**)	**−**
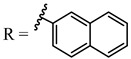	(**22**)	**−**			

**Table 4:** RED experiments: binding percentages with Aβo. Both white chambers of the two RED devices contained 500 µL of phosphate buffer (pH 7.4). In each red chamber, 300 μL of 1 μM ligand was added. One red chamber additionally received 12 μL of 50 μM Aβo (final concentration: 2 μM protein, P), while the other received 12 μL of neurobasal medium (no protein, NP). Samples were shaken for 4 h and analyzed by selective ion monitoring (SIM). Experiments were performed in triplicate. Binding percentage was calculated as [1 − SIM(P)/SIM(NP)] × 100.

Compound	Binding Percentage (%)
**20**	4.2 ± 9.5
**43**	0.6 ± 13.5
**52**	−2.1 ± 9.5

**Table 5:** Microsomal metabolism (% metabolized after incubation) and aqueous solubility (pH 7.4) of selected phenylselenogalactoside derivatives.

Compound	Human Microsomes ^1^	Mouse Microsomes ^1^	Rat Microsomes ^1^	Dog Microsomes ^1^	Solubility (µM)
**19**	100	100	95.4	100	21
**20**	5.7	9.5	49.2	9.3	>100
**11**	99.8	100	96.6	88.9	11
**12**	0.0	13.8	12.5	0.0	>100
**15**	100	100	100	100	11
**16**	6.8	25.8	21.1	15.5	41
**33**	100	100	98.6	100	21
**34**	3.8	14.9	12.9	0.0	>100

^1^ Percentage metabolized.

**Table 6:** Predicted physicochemical properties of selected phenylselenogalactoside derivatives (SwissADME [26]).

Compound	MW (g/mol)	logP (Consensus)	TPSA (Å^2^)	HBD	HBA	Lipinski (Violations)
**19**	617.43	1.66	130.12	1	12	1
**20**	491.32	0.54	111.91	4	9	0
**11**	566.43	1.59	117.23	1	9	1
**12**	440.32	0.49	99.02	4	6	0
**15**	580.46	1.87	117.23	1	9	1
**16**	454.35	0.77	99.02	4	6	0
**33**	563.46	1.14	129.26	2	8	1
**34**	437.35	0.02	111.05	5	5	0

## Data Availability

The original contributions presented in this study are included in the article. Further inquiries can be directed to the corresponding author.

## Abbreviations

The following abbreviations are used in this manuscript but are not explicitly defined in the main text:

ADME	Absorption, distribution, metabolism and excretion
DMSO	Dimethylsulfoxide
HBA	Hydrogen bond acceptor
HBD	Hydrogen bond doner
HPLC	High-performance liquid chromatography
LC–MS	Liquid chromatography–mass spectrometry
mGluR5	Metabotropic glutamate receptor 5
MW	Molecular weight
MWCO	Molecular weight cut-off
NMDA	*N*-Methyl-d-aspartate
SIM	Selective ion monitoring
STD-NMR	Saturation transfer difference nuclear magnetic resonance
TPSA	Topological polar surface area
